# Deltacoronavirus Modulates circRNA *cGLIS3* Metabolism to Evade Host Antiviral Response

**DOI:** 10.1002/advs.76822

**Published:** 2026-07-27

**Authors:** Liuyang Du, Liping Wang, Sujing Diao, Juan Li, Lei Zhao, Yan Yan, Haimin Li, Weiren Dong, Wei Wu, Jinyan Gu, Jiyong Zhou

**Affiliations:** ^1^ MOA Key Laboratory of Animal Virology, Zhejiang Provincial Engineering Research Center of Animal Biological Products Zhejiang University Center For Veterinary Sciences Hangzhou China; ^2^ State Key Laboratory For Diagnosis and Treatment of Infectious Diseases First Affiliated Hospital, Zhejiang University Hangzhou China

**Keywords:** antiviral innate immunity, circRNA metabolism, coronavirus, m^6^A modification, RNA binding protein

## Abstract

Circular RNAs (circRNAs) are dynamically remodeled during infection, yet how viruses exploit circRNA‐RNA binding protein (RBP) circuits remains poorly understood. Here, we report a *cGLIS3*‐IGF2BP2‐linked TNF‐α regulatory axis triggered by deltacoronavirus infection to inhibit antiviral innate immunity. The host m^6^A reader insulin‐like growth factor 2 mRNA‐binding protein 2 (IGF2BP2) and viral nucleocapsid (N) protein promote biogenesis of the m^6^A‐modified *cGLIS3* by strengthening the *GLIS3 exon 3* circulation. Interaction assay reveals that K‐Homology domain of IGF2BP2 or the linker region of viral protein N binds to *GLIS3* pre‐mRNA to achieve *cGLIS3* biogenesis, respectively. IGF2BP2 stabilizes the m^6^A‐modified *cGLIS3* against RNase L‐mediated degradation. *cGLIS3* attenuates IGF2BP2‐driven TNF‐α induction to facilitate deltacoronavirus replication by accelerating K48‐linked ubiquitin degradation of IGF2BP2. Together, our findings uncover a coronavirus‐elicited circRNA‐RBP crosstalk circuit to suppress innate immunity, establishing *cGLIS3* as a mechanistically defined regulator of virus‐host interactions.

## Graphical abstract

This study reveals that both deltacoronavirus nucleocapsid protein and host RNA binding protein IGF2BP2 promote circular RNA *GLIS3* (*cGLIS3*) biogenesis by binding to *GLIS3* pre‐mRNA. The m^6^A modification‐mediated *cGLIS3*‐IGF2BP2 interaction weakens RNase L‐mediated degradation of *cGLIS3* while facilitates a ubiquitin‐dependent degradation of IGF2BP2, thus downregulating TNF‐α expression, uncovering a coronavirus‐elicited circRNA‐RBP crosstalk circuit to suppress innate immunity.

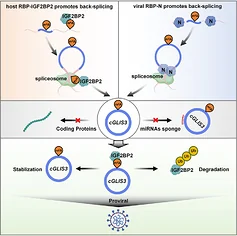

## 1 Introduction

Coronaviruses (CoVs) are enveloped, positive‐sense single‐stranded RNA viruses, including *Alphacoronavirus*, *Betacoronavirus*, *Gammacoronavirus*, and *Deltacoronavirus* [1]. Although deltacoronavirus has been identified in diverse avian species, deltacoronavirus in mammals has only been isolated in pigs [2, 3], named porcine deltacoronavirus (PDCoV), which causes acute diarrhea, vomiting, and death in suckling piglets and has spread rapidly in many countries [4, 5]. Recent studies have shown that PDCoV can infect not only pigs, but also calves, chickens, turkeys and mice [6, 7, 8]. Of note, the detection of PDCoV from the plasma of three Haitian children with acute febrile illness highlights its zoonotic potential and raises concerns about cross‐species transmission [9].

Circular RNAs (circRNAs) constitute a distinct class of noncoding RNAs formed through covalent back‐splicing, resulting in closed‐loop structures that lack polarity and poly(A) tails. They are abundantly expressed across tissues and species and have been implicated in diverse biological and pathological processes [10, 11, 12], including transcriptional or post‐transcriptional regulation [13, 14, 15], miRNA and RNA binding protein (RBP) sequestration [16, 17] and, in some cases, protein translation [18]. CircRNA biogenesis is governed by spliceosomal components, intronic complementary sequences (ICSs), and RBPs such as DHX9, FUS, QKI, and ZC3H14 [19, 20, 21, 22, 23]. Although viral infection reshapes circRNA landscapes and several circRNAs have been reported to modulate viral replication [24, 25, 26, 27], whether virus‐encoded RBPs, particularly the CoV nucleocapsid (N) protein, a multifunctional RBP with high RNA affinity [28, 29, 30] directly engage in circRNA formation remains unknown. Consequently, the major functional roles and upstream regulatory mechanisms of circRNAs during viral infection are still poorly defined.

N^6^‐methyladenosine (m^6^A), the most abundant internal modification in mammalian mRNAs [31, 32, 33], is written by the METTL3‐METTL14‐WTAP complex, erased by ALKBH5 and FTO, and deposited predominantly within RRACH motifs [34, 35, 36]. Distinct m^6^A “reader” families‐YTHDCs, YTHDFs, and IGF2BP1/2/3‐interpret this mark to influence RNA metabolism [37]. Insulin‐like growth factor 2 mRNA‐binding protein 2 (IGF2BP2), which contains two RNA recognition motifs (RRMs) and four K Homology (KH)‐domains [38], has emerged as a key m^6^A reader that binds selected circRNAs, including *circTNPO3* and *circNDUFB2*, to regulate tumor progression [39, 40]. However, how m^6^A‐modified circRNAs and IGF2BP2 interact to influence coronavirus replication remains essentially unexplored. Using PDCoV as a deltacoronavirus model, we identify the circular RNA *GLIS3* (*cGLIS3*) as a host factor that is robustly induced upon viral infection. We show that *cGLIS3* binds IGF2BP2 in an m^6^A‐dependent manner to counteract RNase L‐mediated RNA degradation and antiviral activity, thereby enhancing PDCoV replication through destabilization of IGF2BP2 and suppression of IGF2BP2‐driven TNF‐α expression. We simultaneously demonstrate that the PDCoV N protein cooperates with IGF2BP2 to regulate *cGLIS3* at both the transcriptional and splicing levels. Together, these findings uncover a multilayered mechanism by which a deltacoronavirus rewires host circRNA metabolism and identify *cGLIS3* as a candidate target for antiviral intervention.

## 2 Results

### 2.1 *cGLIS3* is a circRNA without Protein‐Coding Potential

Given our previous identification of a highly abundant circRNA (*cGLIS3*; ID: *ssc_circ_0024481*) in PDCoV‐infected cells [26], we further examined *cGLIS3* expression following exposure to different viruses and viral mimics. As shown in Figure 1A, except for PEDV, SADS‐CoV, and rabies virus, infection with all other RNA viruses tested and transfection with poly (I:C), markedly induced *cGLIS3* upregulation. However, similar changes were not observed in cells infected with DNA virus (herpes simplex virus, HSV) or transfected with poly (dA:dT), indicating that *cGLIS3* preferentially responds to RNA virus associated stimuli.

**FIGURE 1:**
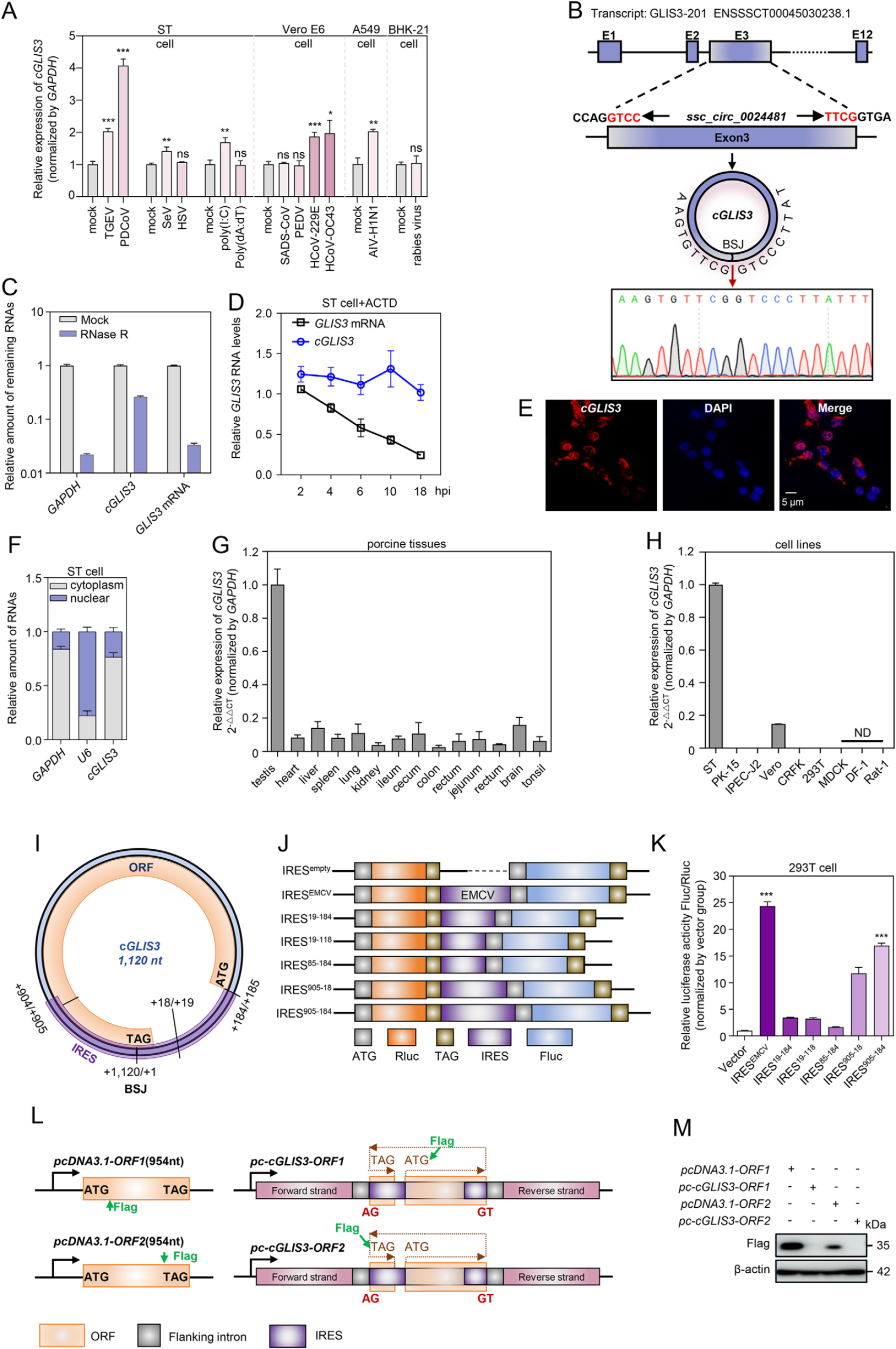
Characterization of porcine *cGLIS3*. (A) RT‐qPCR analysis of *cGLIS3* level in cells treated with indicated viruses or viral mimics for 12 h, respectively. (B) The genomic locus of *cGLIS3* in the *GLIS3* gene within chromosome 1. (C) Resistance of *cGLIS3* to RNase R digestion determined by RT‐qPCR. *GAPDH* and *GLIS3* mRNAs served as negative controls. Relative expression of *cGLIS3* was normalized to the mock‐treated control. (D) RT‐qPCR analysis of *cGLIS3* abundance in ST cells treated with 1 µg mL^−1^ ACTD at the indicated time point. (E) Intracellular localization of *cGLIS3* (red) was determined by Fluorescence in situ hybridization with junction‐specific probes. Scale bars, 5 µm. (F) RT‐qPCR analysis of *cGLIS3* abundance in the cytoplasm and nucleus of ST cells. *GAPDH* and *U6* mRNAs served as cytoplasmic and nuclear markers, respectively. (G and H) RT‐qPCR analysis of *cGLIS3* abundance in porcine tissues (G) and cell lines (H). (I) Schematic representation of the putative ORF, IRES and start/stop sites of *cGLIS3*. (J) Schematic illustration of the putative IRES reporter plasmids based on dual luciferase vector. IRES^EMCV^ served as a positive control. (K) Relative luciferase activity of Fluc/Rluc in HEK‐293T cells transfected with indicated vectors in (J). Firefly luciferase activity was normalized to Renilla activity. Fluc, firefly luciferase; Rluc, renilla luciferase. (L) Schematic representation of the reconstructed expression plasmids based on *pcDNA3.1*(+) or *pc*. A FLAG tag was added directly to downstream/upstream of the start/stop codon (ATG/TAG) of the putative ORF to establish *pcDNA3.1‐ORF1/2* and *pc‐cGLIS3‐ORF1/2*. (M) The expression of potential ORFs within *cGLIS3* was detected by Western blot. Data are presented as mean ± SD (*n* = 3; *p < 0.05, **p < 0.01, ***p < 0.001; ns, no significant; two‐tailed unpaired *t*‐test).

Subsequently, we characterized the molecular features of *cGLIS3*. Sanger sequencing of the back‐splicing junction (BSJ) confirmed the circular nature of *cGLIS3*, which is 1120 nucleotides (nt) in length (Figure 1B). RNase R resistance and stability assays demonstrated that *cGLIS3* is substantially more stable than linear *GLIS3* mRNA (Figure 1C,D). BSJ‐targeting fluorescence in situ hybridization (FISH) and nuclear/cytoplasmic fractionation revealed that *cGLIS3* predominantly localizes to the cytoplasm (Figure 1E,F and Figure S1A,B). Consistent with reports in human and mouse tissues [41, 42], we observed broad *cGLIS3* expression across 14 porcine tissues and multiple cell lines. RT‐qPCR analysis revealed widespread expression, with particularly high abundance in the testis and in ST cells (Figure 1G,H), supporting its conservation across mammalian species.

Although circRNAs are generally categorized as noncoding RNAs, accumulating evidence has revealed that a subset can be translated via embedded internal ribosome entry sites (IRESs) or m^6^A modifications [43, 44, 45]. To determine whether porcine *cGLIS3* possesses protein‐coding potential, we conducted in silico analyses, which predicted a putative open reading frame (ORF) with a size of 954 nucleotides spanning the BSJ as well as a putative IRES element (IRES^905‐184^) upstream of the start codon by IRESfinder (Figure 1I and Figure S1C) [46]. IRES^905‐184^ was further segmented into four fragments (IRES^19‐184^, IRES^19^ ^−118^, IRES^81^ ^−184^, IRES^905‐18^ and IRES^905‐184^, Figure 1J), and their activities were evaluated using a dual‐luciferase reporter assay (Firefly/Renilla), as previously described [43]. Of five putative IRES candidates evaluated, IRES^905‐184^ showed the strongest translational activity of Firefly luciferase (Figure 1K), suggesting a possible translation of ORF. We then constructed a *cGLIS3* overexpression plasmid, named *pc‐cGLIS3*, and a corresponding control vector *pc*, following our previously established protocol (Figure S1D) [26], and verified *cGLIS3* expression efficiency (Figure S1E). Two linear and two circular expression constructs encoding the predicted 317 amino acid (aa) product were generated and transfected into HEK‐293T cells (Figure 1L and Figure S1F). Western blotting detected a robust ∼35 kDa protein from both linear constructs but no detectable protein from either circular construct (Figure 1M), demonstrating that, unlike its murine counterpart (*mmu_circ_0000943*), porcine *cGLIS3* does not undergo translation.

Collectively, these data demonstrate that *cGLIS3* is a circRNA without protein‐coding potential and is conserved across mammalian species.

### 2.2 Coronavirus Replication Hijacks *cGLIS3* without miRNA Sponging Capacity

Numerous viruses hijack differentially expressed host circRNAs to enhance their replication [24, 25, 26]. PDCoV was selected as an RNA virus model to evaluate the function role of *cGLIS3*. Three siRNAs targeting the unique BSJ of *cGLIS3* were designed and evaluated in ST cells (Figure 2A). Among them, *si‐cGLIS3‐2* efficiently silenced *cGLIS3* without notably affecting linear *GLIS3* mRNA levels (Figure 2B). Loss of *cGLIS3* in ST cells led to a significant reduction in viral N protein expression, genomic RNA copies and infectious virus titers (Figure 2C–E). Because endogenous *cGLIS3* expression in ST cells is too high to allow reliable overexpression, we used IPEC‐J2 cells with a low endogenous c*GLIS3* abundance, in which *cGLIS3* can be successfully overexpressed to assess the effects of *cGLIS3* overexpression on PDCoV replication (Figure 2F,G). The overexpression of *cGLIS3* in IPEC‐J2 cells exhibited a promoting effect on N protein levels, viral RNA abundance, and infectious titers (Figure 2H–J). These data support a role of *cGLIS3* in promoting viral replication.

**FIGURE 2:**
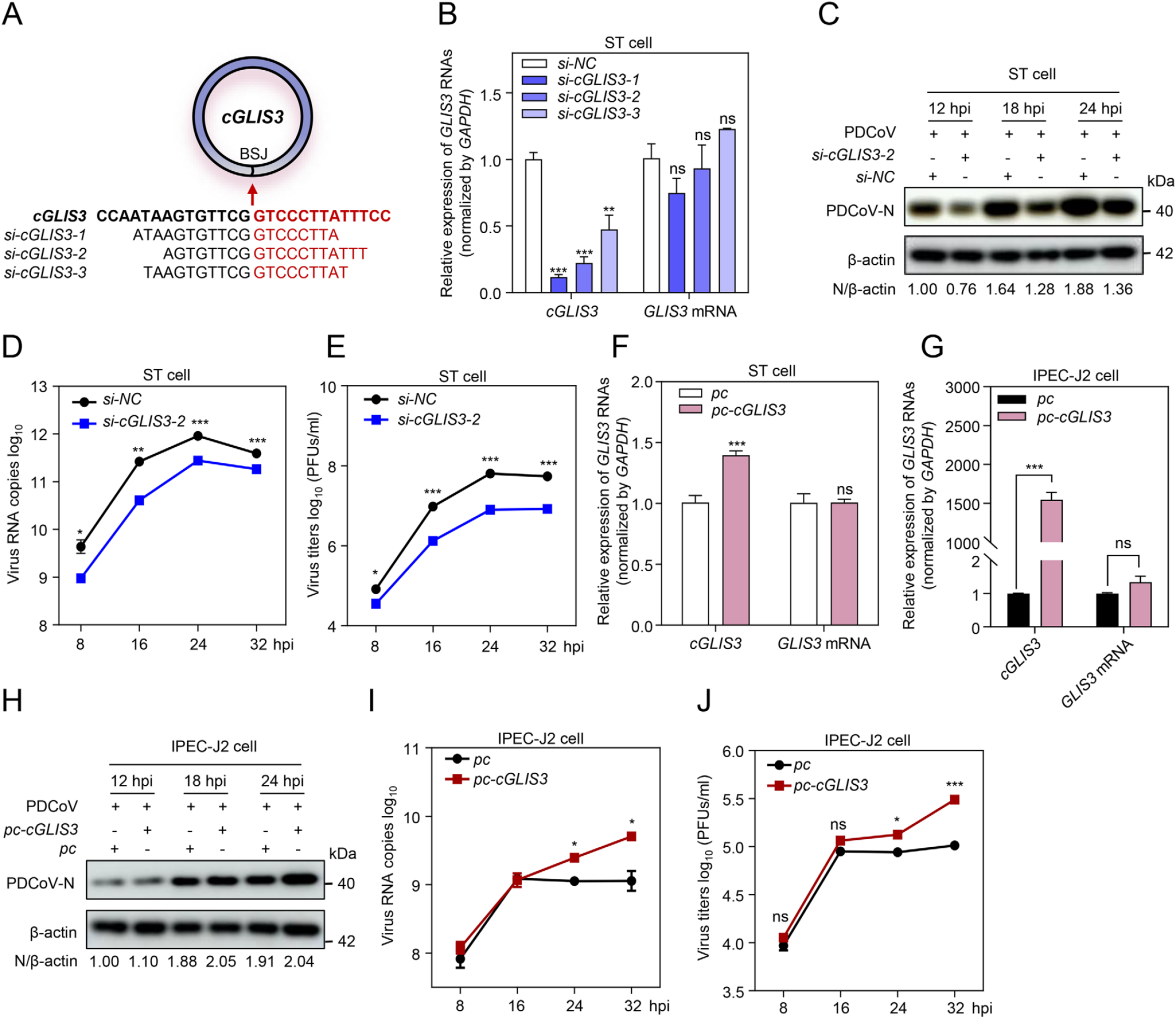
*cGLIS3* promotes PDCoV replication. (A) Schematic representation of the target sequences of siRNAs specific to the back‐splicing junction of *cGLIS3*. (B) RT‐qPCR measured the abundance of *cGLIS3* and *GLIS3* mRNA in ST cells transfected with *cGLIS3*‐siRNAs or *si‐NC* for 48 h. (C) Protein level of PDCoV N in *cGLIS3*‐silenced ST cells. (D and E) Replication kinetics of PDCoV in *cGLIS3*‐silenced cells. ST cells were transfected with *si‐cGLIS‐2* or *si‐NC* for 48 h and then infected with PDCoV at an MOI of 0.1 for 8, 16, 24, and 32 h. Cells were subjected to absolute RT‐qPCR of PDCoV *M* gene copies (D) and plaque assays (E). (F and G) RT‐qPCR analysis of *cGLIS3* and *GLIS3* mRNA abundances in ST (F) or IPEC‐J2 (G) cells transfected with *pc* or *pc‐cGLIS3*, respectively. (H) Protein level of PDCoV N in *cGLIS3*‐overexpressed IPEC‐J2 cells. (I and J) Replication kinetics of PDCoV replication in *cGLIS3*‐overexpressed cells. IPEC‐J2 cells were transfected with *pc* or *pc‐cGLIS3* for 24 h, and then infected with PDCoV at an MOI of 0.1 for another 8, 16, 24, and 32 h, respectively. Cells are then subjected to absolute RT‐qPCR of PDCoV *M* gene (I) and plaque assays (J). The protein bands shown in panel C and H were quantified using ImageJ software. Data are presented as mean ± SD (*n* = 3; *p < 0.05, **p < 0.01, ***p < 0.001; ns, no significant; two‐tailed unpaired *t*‐test).

Given prior evidence that many circRNAs harbor multiple miRNA binding sites [47], we explored whether *cGLIS3* exerts a miRNA adsorbing ability. RNA immunoprecipitation (RIP) and following RT‐qPCR results exhibited a significant enrichment of *cGLIS3* by FLAG‐tagged porcine AGO2 (*flag‐poAGO2*) relative to a *flag‐GFP* control (Figure S2A,B), suggesting the potential miRNA sponging function of *cGLIS3*. Next, to identify specific miRNAs potentially sponged by *cGLIS3*, we employed miRanda and RNAhybrid algorithms and shortlisted 12 candidate miRNAs (Figure S2C). For experimental validation, we cloned the full‐length *cGLIS3* sequence into the pmirGLO luciferase reporter (*cGLIS3‐FL*) and co‐transfected it with candidate miRNA mimics. Among these, *novel_878* and *novel_578* markedly reduced luciferase activity (Figure S2D). To assess binding specificity, we generated reporters with mutated binding sites (*cGLIS3‐FL‐878/578‐mut*), as well as reporters containing only the predicted target sites (*cGLIS3‐878*/*578*) and their corresponding mutants (*cGLIS3‐878*/*578‐mut*) (Figure S2E,F). Surprisingly, luciferase activity from both the full‐length mutant constructs and the isolated target site reporters remained significantly suppressed by the corresponding miRNA mimics (Figure S2G–J), indicating that *cGLIS3* does not specifically bind miRNAs.

Collectively, these data demonstrate that RNA virus replication exploits *cGLIS3* without miRNA binding ability.

### 2.3 The m^6^A‐Modified *cGLIS3* Interacts with the KH‐Domain of IGF2BP2

RBPs could bind to specific motifs in intron regions to modulate gene alternative splicing and circRNA generation [17, 19, 21], and circRNAs can also act as platforms or sponges for proteins [17, 48]. Thereby, we used the circAtlas database to predict potential RBP partners and regulators of *cGLIS3*. Among the predicted candidates, IGF2BP2 and EIF4A3 emerged with the highest number of predicted binding sites within the *cGLIS3* sequence and its flanking intronic regions (Figure 3A). RIP followed by RT‐qPCR confirmed that IGF2BP2, EIF4A3, and another two RBPs, muscleblind‐like 1 (MBNL1) and fused in sarcoma (FUS) physically associated with *cGLIS3* (Figure 3B,C and Figure S3A–C). Notably, *cGLIS3* showed the strongest enrichment to IGF2BP2. Consequently, IGF2BP2 was selected for further mechanistic investigation.

**FIGURE 3:**
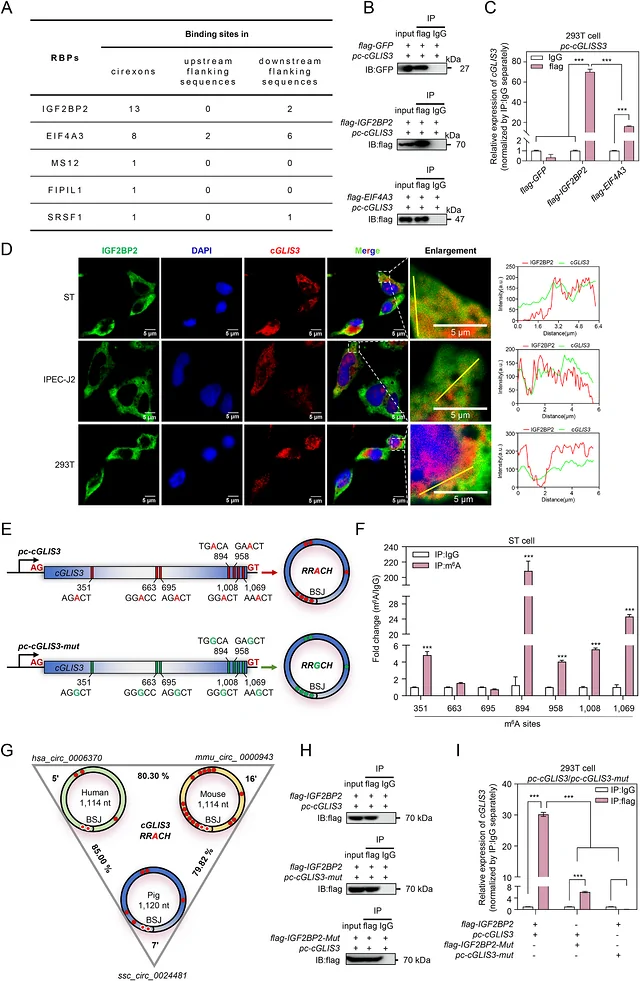
Porcine *cGLIS3* directly interacts with IGF2BP2. (A) The potential RBP sites in *cGLIS3* or its flanking sequences predicted by circAtlas. (B and C) RIP and RT‐qPCR experiments were performed to check *cGLIS3* association with IGF2BP2 or EIF4A3, respectively. After co‐transfection with *pc‐cGLIS3* and *flag‐IGF2BP2* or *flag‐EIF4A3* or *flag‐GFP* for 24 h, HEK‐293T cells were lysed for IP (B). IP complex was subjected to RT‐qPCR analysis (C). (D) Representative images and colocalization analysis of *cGLIS3* (red) and IGF2BP2 (green) in ST cells, IPEC‐J2 cells, or HEK‐293T cells. ST cells or IPEC‐J2 cells were transfected with *flag‐IGF2BP2* plasmid, and HEK‐293T were co‐transfected with *pc‐cGLIS3* and *flag‐IGF2BP2* plasmids. *cGLIS3* and IGF2BP2 were labeled by its junction‐specific probe (red) and a FITC‐conjugated second antibody (green), respectively. Fluorescence intensity profiles of the red and green fluorescent signals along two different colored cross‐section lines of an enlarged merged image are shown in the right panel. Scale bars, 5 µm. (E) Schematic representation of the putative m^6^A modification sites in *cGLIS3* sequence. The predicted site (red) in *pc‐cGLIS3* was mutated in *pc‐cGLIS3‐mut* as indicated in green. (F) m^6^A enrichment of *cGLIS3* putative sites in ST cells by MeRIP‐qPCR. Results are presented relative to those obtained with IgG. (G) Schematic representation of the m^6^A modification sites in porcine, human or mouse *cGLIS3*. (H and I) RIP and RT‐qPCR assays that KH domain of IGF2BP2 is essential for m^6^A‐mediated IGF2BP2‐*cGLIS3* interaction. After co‐transfection with *pc‐cGLIS3* (or *pc‐cGLIS3‐mut*) and *flag‐IGF2BP2* (or *flag‐IGF2BP2‐Mut*) for 24 h, HEK‐293T cells were lysed for IP and Western blot (H). IP complex was subjected to RT‐qPCR analysis (I). Data are presented as mean ± SD (*n* = 3; *p < 0.05, **p < 0.01, ***p < 0.001; ns, no significant; two‐tailed unpaired *t*‐test).

Next, RNA FISH combined with immunofluorescence staining was performed to assess the subcellular localization of IGF2BP2 and *cGLIS3* in different cells. The results showed that the green fluorescence signal (IGF2BP2) and the red fluorescence signal (*cGLIS3*) were extensively overlapped in the cytoplasm, as indicated by yellow signals in the merged images. Fluorescence intensity analysis of the magnified regions further confirmed the high degree of cytoplasmic co‐localization of *cGLIS3* and IGF2BP2 in different cells (Figure 3D). Similarly, RIP‐qPCR assay also proved this tight interaction in PDCoV infection conditions (Figure S3D,E), suggesting spatial proximity conducive to functional interaction. As IGF2BP2 is a well‐established m^6^A “reader” protein, the *cGLIS3* sequence was then examined to identify m^6^A consensus motifs (RRACH) by SRAMP (Figure S3A) [49]. Seven putative m^6^A sites were predicted (Figure 3E, top panel). To test their functional relevance, we constructed an m^6^A‐deficient *cGLIS3* overexpression vector *(pc‐cGLIS3‐mut*) by mutating RRACH motifs to RRGCH (Figure 3E, bottom panel and Figure S3F). Methylated RIP (MeRIP) assay revealed that five of the predicted sites in *cGLIS3*, particularly sites 894 and 1069, were significantly enriched, confirming the presence of functional m^6^A marks in *cGLIS3* (Figure 3F). To assess evolutionary conservation of these m^6^A‐modified sites, we aligned the *cGLIS3* sequences from pig, human, and mouse. Two highly conserved m^6^A‐modified sites are identified to be “GGACU” (pig site‐1008, human site‐1002, mouse site‐1019) and “AAACU” (pig site‐1069, human site‐1063, mouse site‐1063) (Figure 3G), suggesting that *cGLIS3* from different species of mammals has conserved m^6^A modification sites.

Considering that the KH‐domain of IGF2BP2 mediates its recognition of m^6^A‐modified targets [38], we engineered IGF2BP2 mutants with GxxG to GEEG substitutions across all four KH‐domains (IGF2BP2‐Mut), as well as a deletion mutant lacking the two RNA recognition motifs (IGF2BP2‐ΔRRM) (Figure S3G). RIP followed by RT‐qPCR showed that disrupting either the KH‐domains of IGF2BP2 or the m^6^A motifs of *cGLIS3* significantly abrogated their interaction (Figure 3H,I), indicating that the m^6^A‐modified *cGLIS3* binds to the KH‐domain of IGF2BP2.

Collectively, these data demonstrate that the m^6^A modification “reader” IGF2BP2 recognizes the m^6^A‐modified sites of *cGLIS3* by its KH‐domain.

### 2.4 The m^6^A‐Modified cGLIS3 Evades RNase L‐Mediated Degradation via cGLIS3‐IGF2BP2 Interaction

Given that IGF2BP2 is essential for mRNA stability [38, 50], we investigated whether IGF2BP2‐*cGLIS3* interaction affects *cGLIS3* abundance. Three siRNAs were designed to silence IGF2BP2, and *si‐IGF2BP2‐3* showed superior knockdown efficiency (Figure 4A). In ST cells, depletion of IGF2BP2 significantly reduced endogenous *cGLIS3* levels, whereas IGF2BP2 overexpression increased *cGLIS3* abundance (Figure 4B,C). Mutational analysis further demonstrated that disruption of KH‐domain, particularly in IGF2BP2‐Mut, markedly decreased *cGLIS3* levels, whereas deletion of the RRM elevated *cGLIS3* relative to wild‐type IGF2BP2 (Figure 4D). In HEK‐293T cells, exogenous *cGLIS3* levels were similarly reduced by either IGF2BP2‐Mut or *cGLIS3‐mut* (Figure 4E). Overexpression of porcine m^6^A methyltransferases METTL3, METTL14, or WTAP, especially METTL14 and WTAP, substantially increased *cGLIS3* abundance (Figure 4F) or stability (Figure 4G), but had no effect on the m^6^A‐deficient *cGLIS3* mutant (Figure 4F), demonstrating that *cGLIS3* stability requires both m^6^A modification and IGF2BP2 binding. Furthermore, following actinomycin D (ACTD) treatment, endogenous *cGLIS3* levels were higher in IGF2BP2 overexpressing cells rather than in IGF2BP2‐Mut overexpressing cells (Figure 4H), and both IGF2BP2‐Mut and *cGLIS3‐mut* accelerated *cGLIS3* decay (Figure 4I). These results demonstrate that IGF2BP2 stabilizes the m^6^A‐modified *cGLIS3* in an IGF2BP2 KH‐domain‐dependent manner.

**FIGURE 4:**
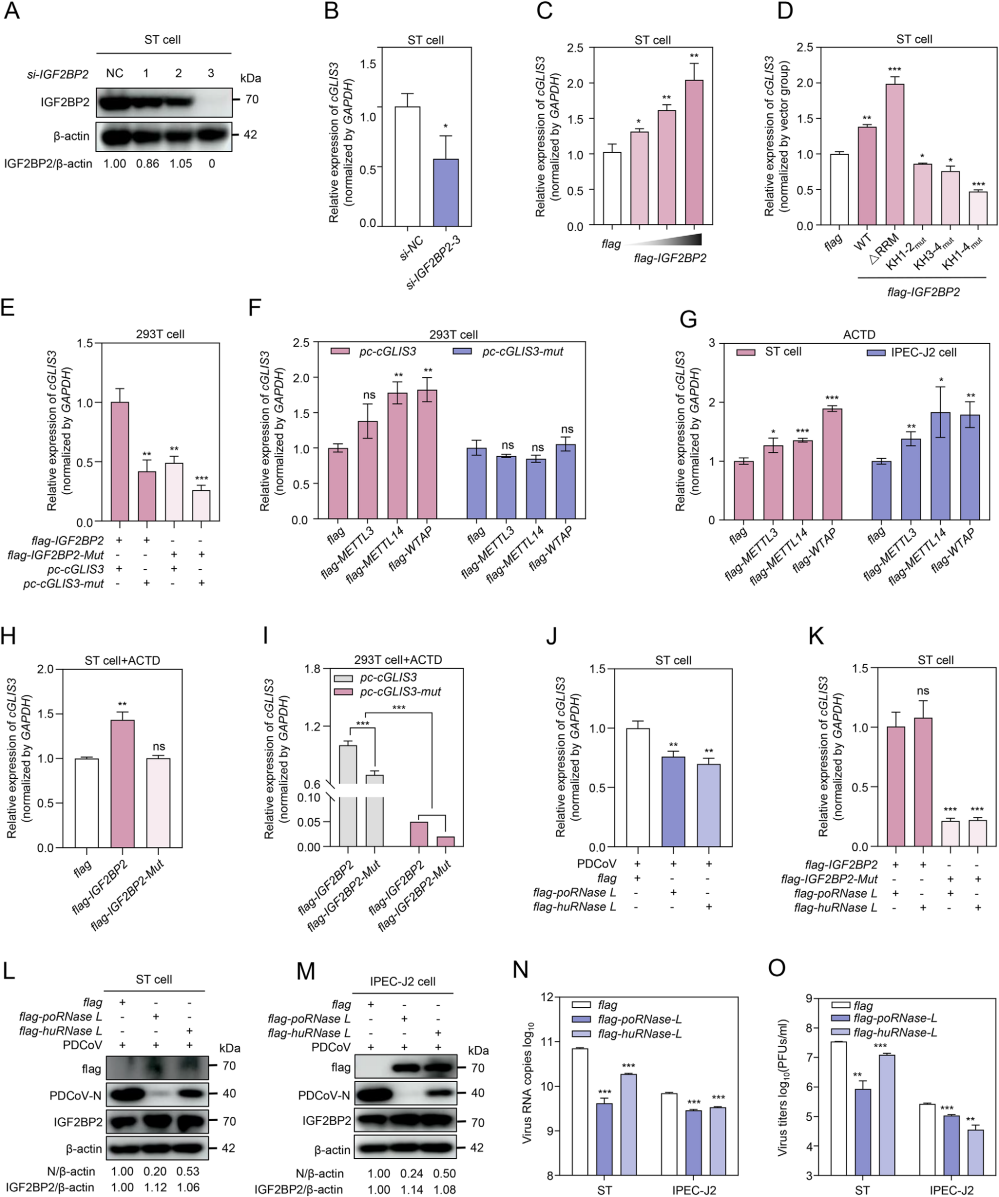
*cGLIS3*‐IGF2BP2 interaction restricts the degradation of *cGLIS3* by RNase L. (A) Immunoblot analysis of ST cells transfected with specific siRNAs targeting to *IGF2BP2* mRNA or *si‐NC*. (B and C) The abundance of *cGLIS3* in IGF2BP2‐silenced (B) or ‐overexpressed (C) ST cells examined by RT‐qPCR. (D) The *cGLIS3* level in ST cells transfected with different IGF2BP2 mutants examined by RT‐qPCR. (E) RT‐qPCR analysis of *cGLIS3* level in HEK‐293T cells co‐transfected with *pc‐cGLIS3* (or *pc‐cGLIS3‐mut*) and *flag‐IGF2BP2* (or *flag‐IGF2BP2‐Mut*), respectively. (F) The *cGLIS3* abundance in HEK‐293T cells co‐transfected with *pc‐cGLIS3* (or *pc‐cGLIS3‐mut*) and porcine methyltransferases (METTL3, METTL14 or WTAP), respectively. (G) The *cGLIS3* abundance in porcine cells transfected with porcine methyltransferases (METTL3, METTL14 or WTAP), respectively. After transfected with porcine methyltransferases (METTL3, METTL14 or WTAP) for 24 h, ST cell or IPEC‐J2 cell was treated with ACTD (1 mg mL^−1^) for another 12 h, respectively. (H) The *cGLIS3* level in IGF2BP2 (or IGF2BP2‐Mut)‐overexpressed ST cells with ACTD treatment (1 mg mL^−1^) for 12 h, respectively. (I) The *cGLIS3* level in HEK‐293T cells co‐transfected with *pc‐cGLIS3* (or *pc‐cGLIS3‐mut*) and *flag‐IGF2BP2* (or *flag‐IGF2BP2‐Mut*) followed by ACTD treatment (1 mg mL^−1^) for 12 h, respectively. (J) RT‐qPCR analysis of *cGLIS3* level in RNase L (pig or human originated)‐transfected ST cells with PDCoV infection at an MOI of 0.1 for 18 h. (K) RT‐qPCR analysis of *cGLIS3* level in ST cells co‐transfected with RNase L (pig or human originated) and IGF2BP2 (or IGF2BP2‐Mut) and infected with PDCoV at an MOI of 0.1 for 18 h. (L and M) Protein levels of IGF2BP2 and PDCoV N in RNase L‐overexpressed ST cells (L) or IPEC‐J2 cells (M) with PDCoV infection at an MOI of 0.1 for 18 h. (N and O) RNase L inhibits PDCoV replication. ST cells or IPEC‐J2 cells were transfected with RNase L, followed by the infection of PDCoV at an MOI of 0.1 for 18 h. Virus titers were detected by absolute RT‐qPCR (N) and plaque assays (O). The protein bands shown in panel A, L, and M were quantified using ImageJ software. Data are presented as mean ± SD (*n* = 3; *p < 0.05, **p < 0.01, ***p < 0.001; ns, no significant; two‐tailed unpaired *t*‐test).

RNase L activation during viral infection has been reported to drive global circRNA degradation [48]. Consistent with this, overexpression of either porcine or human RNase L markedly reduced *cGLIS3* abundance following PDCoV infection (Figure 4J). Notably, this degradation was rescued by wild‐type IGF2BP2 but not by KH‐ domain‐mutant IGF2BP2 (Figure 4K), suggesting that IGF2BP2 binding protects *cGLIS3* from RNase L‐mediated degradation. Given the well‐established antiviral activity of RNase L [51], we next assessed its impact on PDCoV replication. RNase L overexpression increased IGF2BP2 protein levels while significantly reducing PDCoV N protein, viral RNA, and viral titers in both ST and IPEC‐J2 cells (Figure 4L–O). These findings indicate that RNase L restricts PDCoV replication at least in part through *cGLIS3* degradation, whereas m^6^A‐dependent *cGLIS3*‐IGF2BP2 interaction enhances the stabilization of *cGLIS3* and attenuates the antiviral effect of RNase L.

### 2.5 IGF2BP2 Enhances the Biogenesis of *cGLIS3*

Given that IGF2BP2 increases *cGLIS3* level and has the binding sites in *cGLIS3* flanking intronic sequences, we next evaluated the effects of IGF2BP2 on c*GLIS3* biogenesis at the levels of transcription and splicing (Figure 5A). The dual‐luciferase assay revealed that IGF2BP2 significantly enhanced *GLIS3* promoter activity (Figure 5B), consistent with the effect on *cGLIS3* expression. Based on *GLIS3* promoter activity, we re‐predicted the precise *GLIS3* promoter using Promoter 2.0 [52] and four primers were designed by the given score and putative position to verify the binding of IGF2BP2 with *GLIS3* promoter (Figure 5C). Chromatin immunoprecipitation (ChIP)‐qPCR assay showed that the 507–709 nt segment was significantly enriched (Figure 5D), indicating that IGF2BP2 could bind to *cGLIS3* promoter directly. These demonstrate that *cGLIS3* is regulated at transcriptional level by IGF2BP2.

**FIGURE 5:**
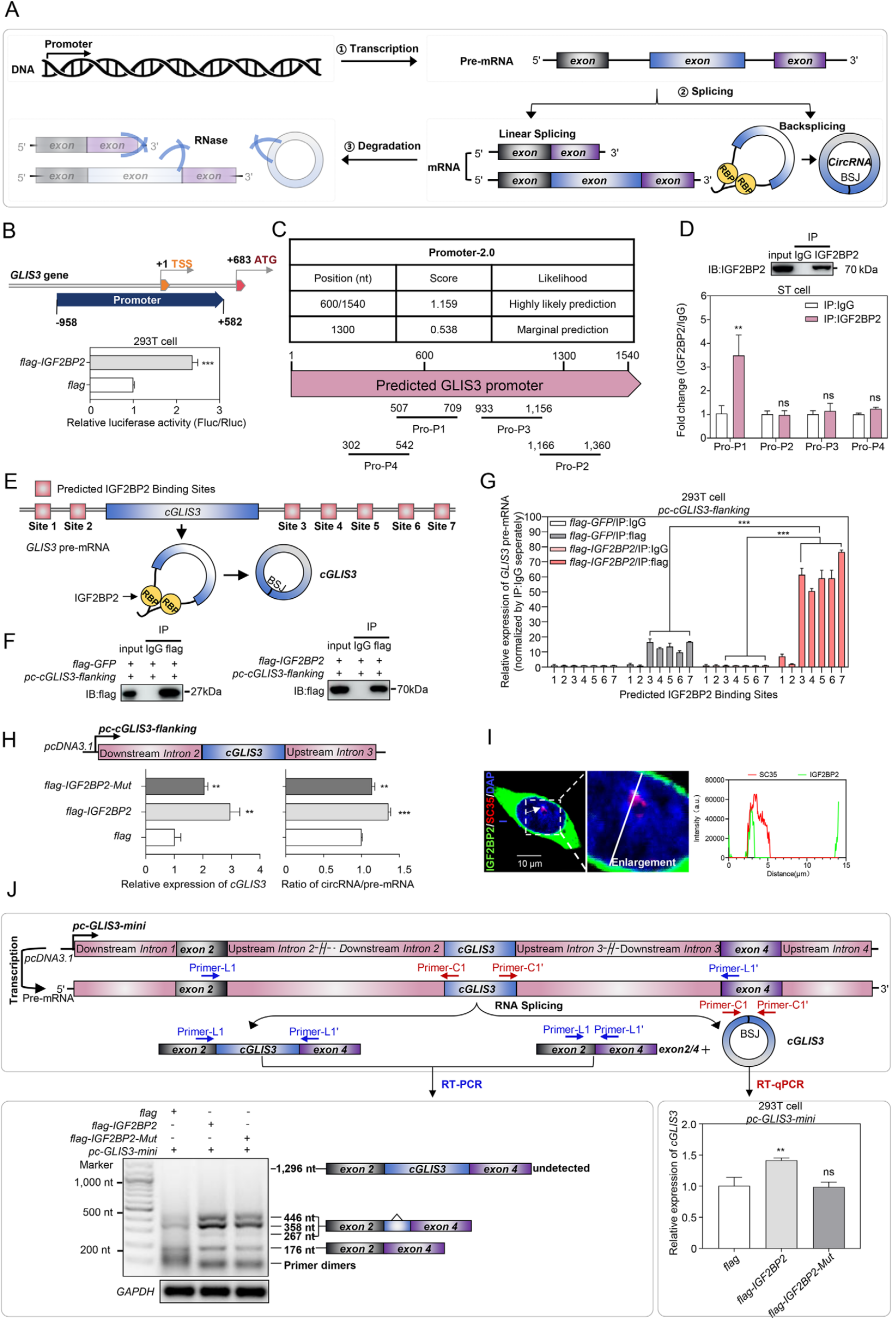
IGF2BP2 enhances the biogenesis of *cGLIS3*. (A) Schematic representation of IGF2BP2 potential stage in modulating *cGLIS3* metabolism. (B) A dual‐luciferase reporter assay determined the effect of IGF2BP2 on *GLIS3* promoter activity. (C) Schematic representation of the predicted region of *GLIS3* promoter and corresponding primer designs for ChIP. (D) The combination of IGF2BP2 and *GLIS3* promoter checked by ChIP. ST cells were lysed for IP and Western blot. IP complex was subjected to RT‐qPCR analysis. (E) Schematic of *GLIS3* pre‐mRNA that predicted binding sites of IGF2BP2 in *pc‐cGLIS3‐flanking*. (F and G) The combination of IGF2BP2 with *GLIS3* pre‐mRNA examined by RIP and RT‐qPCR. After co‐transfection with *flag‐IGF2BP2* (or *flag‐GFP*) and *pc‐cGLIS3‐flanking* for 24 h, HEK‐293T cells were lysed for IP and Western blot (F). IP complex was subjected to RT‐qPCR analysis (G). (H) The effect of IGF2BP2 on *cGLIS3* splicing. HEK‐293T cells were co‐transfected with *pc‐cGLIS3‐flanking* and indicated RBP plasmids, respectively. RT‐qPCR measured the level of *cGLIS3* (left) and the ratio of *cGLIS3*/*GLIS3* pre‐mRNA was analyzed (right). (I) Representative immunofluorescence co‐staining of IGF2BP2 (green) and a nuclear speckle marker SC35 (red). Fluorescence intensity profiles of the red and green fluorescent signals along two different colored cross‐section lines of an enlarged merged image are shown in the right panel. Scale bars, 10 µm. (J) Minigene assay to determine the effect of IGF2BP2 on *cGLIS3* splicing. HEK‐293T cells were co‐transfected with *pc‐cGLIS3‐mini* and indicated RBP plasmids, respectively. RT‐PCR detected the splicing isoforms (left) and RT‐qPCR analyzed the *cGLIS3* level (right). Data are presented as mean ± SD (*n* = 3; *p < 0.05, **p < 0.01, ***p < 0.001; ns, no significant; two‐tailed unpaired *t*‐test).

To further investigate IGF2BP2 roles in splicing, we generated a *cGLIS3* back‐splicing reporter (*pc‐cGLIS3‐flanking*) by inserting a 1,091 nt upstream intron, a 1,120 nt *cGLIS3* linear sequence, and a 1,207 nt downstream intron into the *pcDNA3.1(+)* vector (Figure S4A). The CMV promoter of this construct eliminated interference from the endogenous *GLIS3* promoter (Figure S4B). Predicted IGF2BP2 binding sites within the flanking introns were annotated in Figure 5E and Figure S4C, and RIP‐qPCR confirmed IGF2BP2 binding to sites 3–7 of *GLIS3* pre‐mRNA (Figure 5F,G). IGF2BP2 overexpression significantly increased *cGLIS3* levels and the circRNA/pre‐mRNA ratio by using *pc‐cGLIS3‐flanking* (Figure 5H), indicating that the binding of IGF2BP2 to flanking introns promotes *cGLIS3* back‐splicing. Consistently, a chimeric back‐splicing reporter (*pc‐circTNFAIP3‐flanking*) containing *cGLIS3* flanking introns and a parallel 310 nt *circTNFAIP3* linear sequence produced similar results (Figure S4D,E). Furthermore, IGF2BP2 co‐localized with SC35, a nuclear speckle marker (Figure 5I and Figure S4F), supporting their roles in splicing regulation.

To further validate these findings, we employed minigene back‐splicing reporters (*pc‐GLIS3‐mini*) containing *exon 3*, shortened flanking introns, and two flanking exons (Figure S4G). Based on this splicing model, three major splicing isoforms were anticipated: two linear isoforms (*exon 2/3/4*, 1,296 nt; *exon 2/4*, 176 nt) from canonical splicing and one back‐splicing isoform of *exon 3* (*cGLIS3*, 1,120 nt) (Figure 5J, upper panel). A higher abundance of *exon 2/4* and *cGLIS3* isoforms served as an indicator of increased back‐splicing efficiency. RT‐qPCR analysis demonstrated that IGF2BP2 significantly enhanced *cGLIS3* splicing in *pc‐GLIS3‐mini* construct, whereas KH‐domain‐mutant IGF2BP2 failed to exert this effect (Figure 5J, lower right panel). Subsequently, Minigene‐derived linear isoforms were analyzed via RT‐PCR using the indicated primers‐L1/L1′. DNA gel electrophoresis revealed increased *exon 2/4* isoforms in IGF2BP2‐overexpressing cells, but not in KH‐domain‐mutant IGF2BP2 cells, indicating that IGF2BP2 promotes back‐splicing and that IGF2BP2 KH‐domain is essential for IGF2BP2‐mediated splicing regulation. Interestingly, in addition to the expected *exon 2/4* isoforms, we detected three additional bands of varying sizes, confirmed by Sanger sequencing (Figure S4H). These bands corresponded to one type of noncanonical isoforms: *exon 2/4* containing partial *exon 3* fragment (267, 358 and 446 nt). Notably, the abundance of these isoforms correlated with *cGLIS3* levels, suggesting that they also reflect back‐splicing efficiency (Figure 5J, lower left panel). These minigene splicing models support our conclusion that IGF2BP2 binds to *GLIS3* pre‐mRNA to promote *exon 3* exclusion and enhances *cGLIS3* back‐splicing, with the KH‐domain being essential for IGF2BP2 function in this process.

Similarly, during PDCoV infection, *cGLIS3* abundance was increased in IGF2BP2 wild‐type but not KH‐domain‐mutant overexpressing cells, and the supplement of wild‐type IGF2BP2 rather than the KH‐domain‐mutant IGF2BP2 could significantly rescued *cGLIS3* abundance in IGF2BP2‐knockdown ST cells (Figures S4I,J), indicating that IGF2BP2 modulates the synthesis of *cGLIS3* during PDCoV infection.

Together, these findings establish that IGF2BP2 promotes *cGLIS3* biogenesis by enhancing its transcription, splicing and stability.

### 2.6 PDCoV N Protein Strengthens *cGLIS3* Biogenesis via Binding to *GLIS3* Pre‐mRNA

So far, the dysregulation mechanism of host‐derived circRNAs during viral infection remain largely undefined. We speculated that the increased *cGLIS3* level after PDCoV infection might be modulated by viral components. To test this possibility, we examined the effect of PDCoV‐encoded proteins (Pvps) on endogenous *cGLIS3* and *GLIS3* mRNA expression in ST cells. RT‐qPCR analysis revealed that overexpression of nsp3, nsp4, nsp12, and nsp15 significantly decreased both *cGLIS3* and *GLIS3* mRNA levels, whereas nsp7, nsp8, nsp16, N, M, NS7, and NS7a increased their abundance (Figure S5A,B), suggesting that multiple Pvps may contribute to *cGLIS3* biogenesis. Subsequent dual‐luciferase reporter assays demonstrated that viral proteins N, M, NS7, and NS7a rather than nsp3, nsp4, nsp12, and nsp15 enhanced *GLIS3* promoter activity (Figure 6A), indicating that these viral proteins participate in transcriptional regulation of *cGLIS3*. We then investigated the effects of Pvps on *cGLIS3* splicing in HEK‐293T cells using the *pc‐cGLIS3‐flanking* splicing reporter as a marker. The data illustrated in Figure 6B and Figure S5C showed that viral proteins N, M, S, E, nsp7, nsp8 and nsp10, rather than nsp2, nsp4, NS6, NS7, and NS7a, significantly increased *cGLIS3* levels and the ratio between *cGLIS3* and *GLIS3* pre‐mRNA, particularly PDCoV N protein exhibited the strongest splicing effect. This prompts us to further examine its role in *cGLIS3* back‐splicing.

**FIGURE 6:**
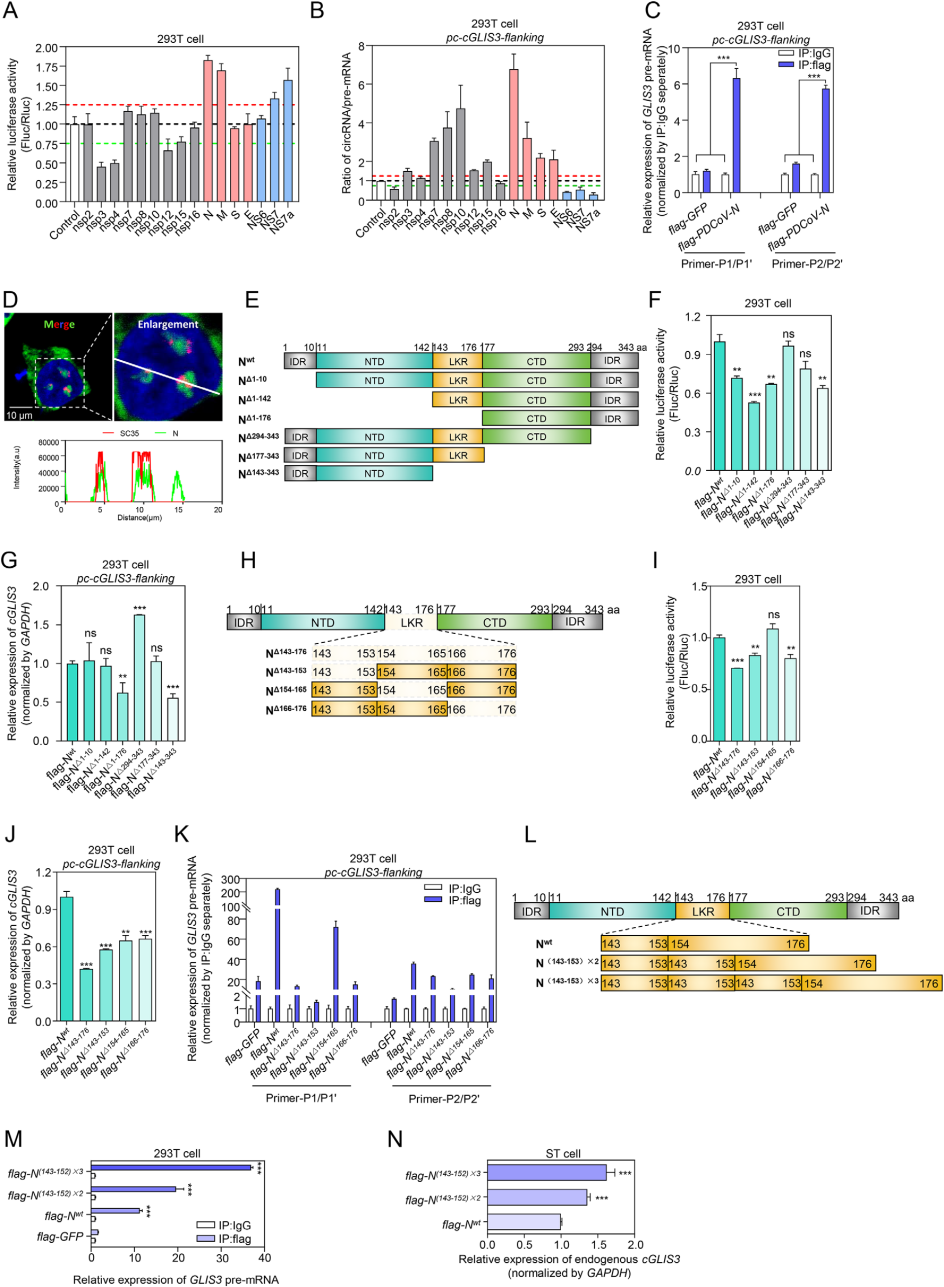
PDCoV nucleocapsid protein modulates *cGLIS3* biogenesis. (A) A dual‐luciferase reporter assay determines the effects of PDCoV viral proteins (Pvps) on *GLIS*3 promoter activity. (B) The ratio of *cGLIS3* and *GLIS3* pre‐mRNA in HEK‐293T cells co‐transfected with *pc‐cGLIS3‐flanking* and Pvps plasmids, respectively. (C) The combination of PDCoV N protein with *GLIS3* pre‐mRNA was examined using Primer‐P1/P1' and Primer‐P2/P2' by RIP and RT‐qPCR. (D) Representative immunofluorescence co‐staining of PDCoV N protein (green) and a nuclear speckle marker SC35 (red). Scale bars, 10 µm. (E) Schematic representation of structural domains for PDCoV N protein. Three intrinsically disordered regions (IDR), the N‐terminal domain (NTD) and C‐terminal domain (CTD) are shown. (F) A dual‐luciferase reporter assay determines the effects of different PDCoV N protein 1–343 aa truncations on *GLIS*3 promoter activity. (G) RT‐qPCR measured *cGLIS3* level in HEK‐293T cells co‐transfected with *pc‐cGLIS3‐flanking* and PDCoV N protein 1–343 aa truncations. (H) The schematic diagram of different truncations for LKR‐domain of PDCoV N protein. (I) A dual‐luciferase reporter assay determines the effects of LKR truncations for PDCoV N protein on *GLIS*3 promoter activity. Firefly luciferase activity in (A, F and I) was normalized to Renilla activity. (J) RT‐qPCR measured *cGLIS3* level in HEK‐293T cells co‐transfected with *pc‐cGLIS3‐flanking* and different LKR truncations of PDCoV N protein. (K) The interplay of *GLIS3* pre‐mRNA with different LKR truncations of PDCoV N protein was examined using Primer‐P1/P1' and Primer‐ P2/P2' by RIP and RT‐qPCR. (L) The schematic diagram of LKR insertion mutants for PDCoV N protein. (M) The combination of *GLIS3* pre‐mRNA with the LKR‐insertion mutants of PDCoV N protein was examined using Primer‐ P2/P2' by RIP and RT‐qPCR. HEK‐293T cells were lysed for IP after co‐transfected with *pc‐cGLIS3‐flanking* and PDCoV N various LKR truncations or *flag‐GFP* for 24 h, respectively. IP complex was subjected to RT‐qPCR analysis. (N) RT‐qPCR measured *cGLIS3* level in ST cells transfected with the LKR‐insertion mutants of PDCoV N protein. Data are presented as mean ± SD (*n* = 3; *p < 0.05, **p < 0.01, ***p < 0.001; ns, no significant; two‐tailed unpaired *t*‐test).

Next, using the *pc‐cGLIS3‐flanking* reporter as a marker, RIP‐qPCR assays revealed that PDCoV N protein significantly enriched *intron 2/exon 3* and *exon 3/intron 3* fragments with two sets of convergent primers P1/P1′ and P2/P2′ (Figure 6C and Figure S5D,E), indicating a direct binding of N protein to *GLIS3* pre‐mRNA. Moreover, PDCoV N co‐localized with SC35 (Figure 6D and Figure S5F), supporting a role of N protein in splicing regulation of *cGLIS3*.

To further identify the domains of PDCoV N protein critical for *cGLIS3* splicing regulation, we generated six truncation mutants (N^Δ1‐10^, N^Δ1‐142^, N^Δ1‐176^, N^Δ294‐343^, N^Δ177‐343^, and N^Δ143‐343^) based on its N‐terminal domain (NTD), C‐terminal domain (CTD) and three intrinsically disordered regions (IDRs). The NTD and CTD are connected by the linker region (LKR), an IDR enriched in serine and arginine residues [53] (Figure 6E and Figure S5G). Dual‐luciferase assays revealed that N^Δ1‐10^, N^Δ1‐142^, N^Δ1‐176^, and N^Δ143‐343^, but not N^Δ294‐343^ or N^Δ177‐343^, significantly reduced *GLIS3* promoter activity, indicating that the N‐terminal IDR, NTD, and LKR domains are required for transcriptional activation of *GLIS3* (Figure 6F). The *pc‐cGLIS3‐flanking* splicing reporter assay showed that N^Δ1‐176^ and N^Δ143‐343^ lacking the LKR‐domain impaired *cGLIS3* back‐splicing, demonstrating that the LKR‐domain is essential for splicing regulation (Figure 6G). Importantly, deletion of LKR (N^Δ143‐176^) did not affect nuclear localization or SC35 co‐localization (Figure S5H), indicating that the LKR of viral protein N is crucial for binding *GLIS3* pre‐mRNA and splicing, and is dispensable for nuclear speckle localization.

To further dissect the LKR‐domain, we constructed three additional truncations N^Δ143‐153^, N^Δ154‐165^ and N^Δ166‐176^ (Figure 6H and Figure S5I). The N^Δ143‐176^, N^Δ143‐153^ and N^Δ166‐176^ reduced *GLIS3* promoter activity (Figure 6I) and diminished endogenous *cGLIS3* and *GLIS3* mRNA levels (Figure S5J,K), suggesting that the 143–153 aa and 166–176 aa segments of the LKR‐domain involve in transcriptional regulation. At the splicing level, all truncations of the LKR significantly reduced *cGLIS3* levels (Figure 6J). RIP‐qPCR assays further showed that the loss of 143–153 aa segment nearly abolished N protein binding to *GLIS3* pre‐mRNA (Figure 6K and Figure S5L), indicating that the residues ^143^FNARGRPQERG^153^ of the LKR is indispensable for splicing regulation. To investigate whether the serial repeats of the residues ^143^FNARGRPQERG^153^ could enhance N protein binding and splicing activity, we engineered N protein variants containing two or three tandem copies of the 143–153 aa fragment within the LKR (Figure 6L). These concatemeric mutants exhibited enhanced ability of binding to *GLIS3* pre‐mRNA (Figure 6M and Figure S5M) and robustly increased endogenous *cGLIS3* levels (Figure 6N).

Together, these Findings Demonstrate That PDCoV N Protein Could Modulate *cGLIS3* Biogenesis, and the Residues ^143^FNARGRPQERG^153^ Within Its LKR‐domain Are Critical for this Activity, Which Provides Direct Evidence That Viral RBPs Can Functionally Mimic Host RBPs to Control circRNA Splicing

### 2.7 The m^6^A‐Modified *cGLIS3* Accelerates Ubiquitin‐Dependent Degradation of IGF2BP2

To elucidate the functional consequences of the *cGLIS3*‐IGF2BP2 interaction, we first examined the impact of *cGLIS3* on endogenous IGF2BP2. Knockdown of *cGLIS3* markedly increased IGF2BP2 protein abundance but did not alter *IGF2BP2* mRNA levels (Figure 7A,B), indicating a post‐transcriptional effect. Consistent with the knockdown results, the wild‐type *cGLIS3* overexpression significantly reduced IGF2BP2 protein levels without affecting its mRNA level, while overexpression of an m^6^A‐deficient *cGLIS3* mutant failed to reduce IGF2BP2 abundance at both protein and mRNA levels (Figure 7C–E). Moreover, the KH‐domain mutant of IGF2BP2 exhibited substantially higher protein levels than the wild‐type protein in the presence of either wild‐type or mutant *cGLIS3* (Figure 7F). These results indicate that m^6^A‐modified *cGLIS3* binds IGF2BP2 and promotes its destabilization.

**FIGURE 7:**
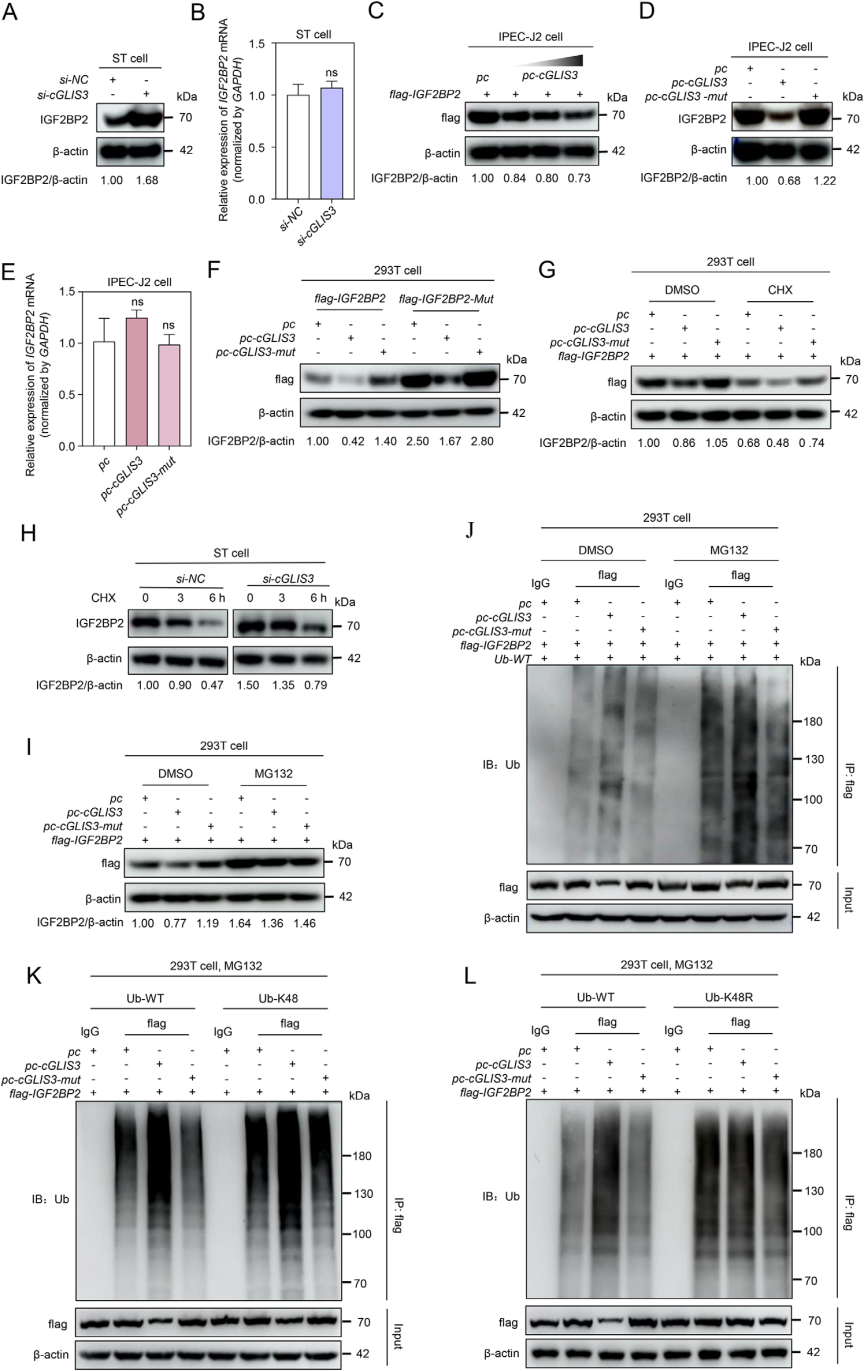
*cGLIS3* promotes ubiquitin‐mediated degradation of IGF2BP2. (A and B) The protein (A) or mRNA (B) level of IGF2BP2 in ST cells with *cGLIS3* silencing. (C and D) The protein level of IGF2BP2 in IPEC‐J2 cells with *cGLIS3* overexpression. (E) The mRNA level of IGF2BP2 in IPEC‐J2 cells transfected with *pc*, *pc‐cGLIS3* or *pc‐cGLIS3‐mut*, respectively. (F) Expression level of IGF2BP2 or IGF2BP2 KH‐mutant (IGF2BP2‐Mut) in HEK‐293T cells co‐transfected with *pc/pc‐cGLIS3/pc‐cGLIS3‐mut* and *flag‐IGF2BP2*/*flag‐IGF2BP2‐Mut*, respectively. (G) IGF2BP2 expression in *cGLIS3* (or *cGLIS3*‐mut) and IGF2BP2 co‐transfected HEK‐293T cells with CHX treatment (100 mg mL^−1^) for 9 h. (H) IGF2BP2 expression in *cGLIS3*‐silenced ST cells treated with CHX (100 mg mL^−1^) for 9 h. (I) IGF2BP2 concentration in *cGLIS3* (or *cGLIS3*‐mut) and IGF2BP2 co‐transfected HEK‐293T cells with MG132 treatment (20 µM) for 9 h. (J–L) Immunoprecipitation detected ubiquitination modification of IGF2BP2 in HEK‐293T cells co‐transfected with *pc/pc‐cGLIS3/pc‐cGLIS3‐mut*, *flag‐IGF2BP2*, and Ub‐wild‐type (WT, J) or Lys48 (K) or Lys48R (L) plasmids, respectively. The protein bands shown in panel A, C, D, F, G, H, and I were quantified using ImageJ software. Data are presented as mean ± SD (*n* = 3; *p < 0.05, **p < 0.01, ***p < 0.001; ns, no significant; two‐tailed unpaired *t*‐test).

We next investigated the mechanism underlying IGF2BP2 downregulation. Cycloheximide (CHX) chase assays revealed that *cGLIS3* overexpression, but not *cGLIS3* knockdown, markedly accelerated IGF2BP2 protein degradation (Figure 7G,H). CHX treatment did not rescue IGF2BP2 levels in *cGLIS3* overexpressing cells, suggesting that *cGLIS3* acts primarily by promoting degradation rather than by inhibiting translation. Notably, the proteasome inhibitor MG132 restored IGF2BP2 protein levels in *cGLIS3* overexpressing cells (Figure 7I), implicating the ubiquitin‐proteasome system in *cGLIS3‐*mediated IGF2BP2 degradation. Consistent with this model, Co‐IP assays showed that *cGLIS3* overexpression robustly enhanced IGF2BP2 ubiquitination, whereas the m^6^A‐deficient *cGLIS3* displayed a markedly reduced effect (Figure 7J and Figure S6A). K48‐ or K63‐linked ubiquitin chains are the two predominant linkage types in the ubiquitination‐proteasome pathway, and the K48‐linked ubiquitin is responsible for ubiquitination‐mediated degradation of IGF2BP2 [54]. Following ubiquitination assays revealed that *cGLIS3* rather than the m^6^A‐mutant *cGLIS3* significantly increased K48‐linked polyubiquitination of IGF2BP2 (Figure 7K). In contrast, both the wild‐type and the m^6^A‐mutant *cGLIS3* even the empty vector could effectively increase IGF2BP2 polyubiquitination after the transfection of K48R mutant (only K48 mutated to R, K48R; Figure 7L). Compared with the wild‐type ubiquitin (Ub‐WT)‐transfected cells, the reduction of IGF2BP2 expression triggered by *cGLIS3* overexpression was weakened in the K48R‐transfected cells (Figure S6B), and the increasing IGF2BP2 level triggered by *cGLIS3* knockdown was enhanced in the K48R‐transfected cells (Figure S6C), suggesting that the m^6^A‐modified *cGLIS3* facilitates IGF2BP2 K48‐linked polyubiquitination.

Collectively, these findings demonstrate that m^6^A‐modified *cGLIS3* facilitates the ubiquitin‐dependent degradation of IGF2BP2.

### 2.8 IGF2BP2 Inhibits PDCoV Replication Independent on M^6^A Modification of TNF‐α

To determine whether *cGLIS3*‐IGF2BP2 interaction modulates RNA virus replication, we overexpressed IGF2BP2 in ST cells followed by PDCoV infection. Notably, IGF2BP2 overexpression significantly reduced viral genomic RNA abundance, viral N protein level, and infectious viral titers (Figure 8A–C). Conversely, siRNA‐mediated knockdown of IGF2BP2 led to marked increase in all three viral replication metrics (Figure 8D–F), revealing an antiviral role of IGF2BP2.

**FIGURE 8:**
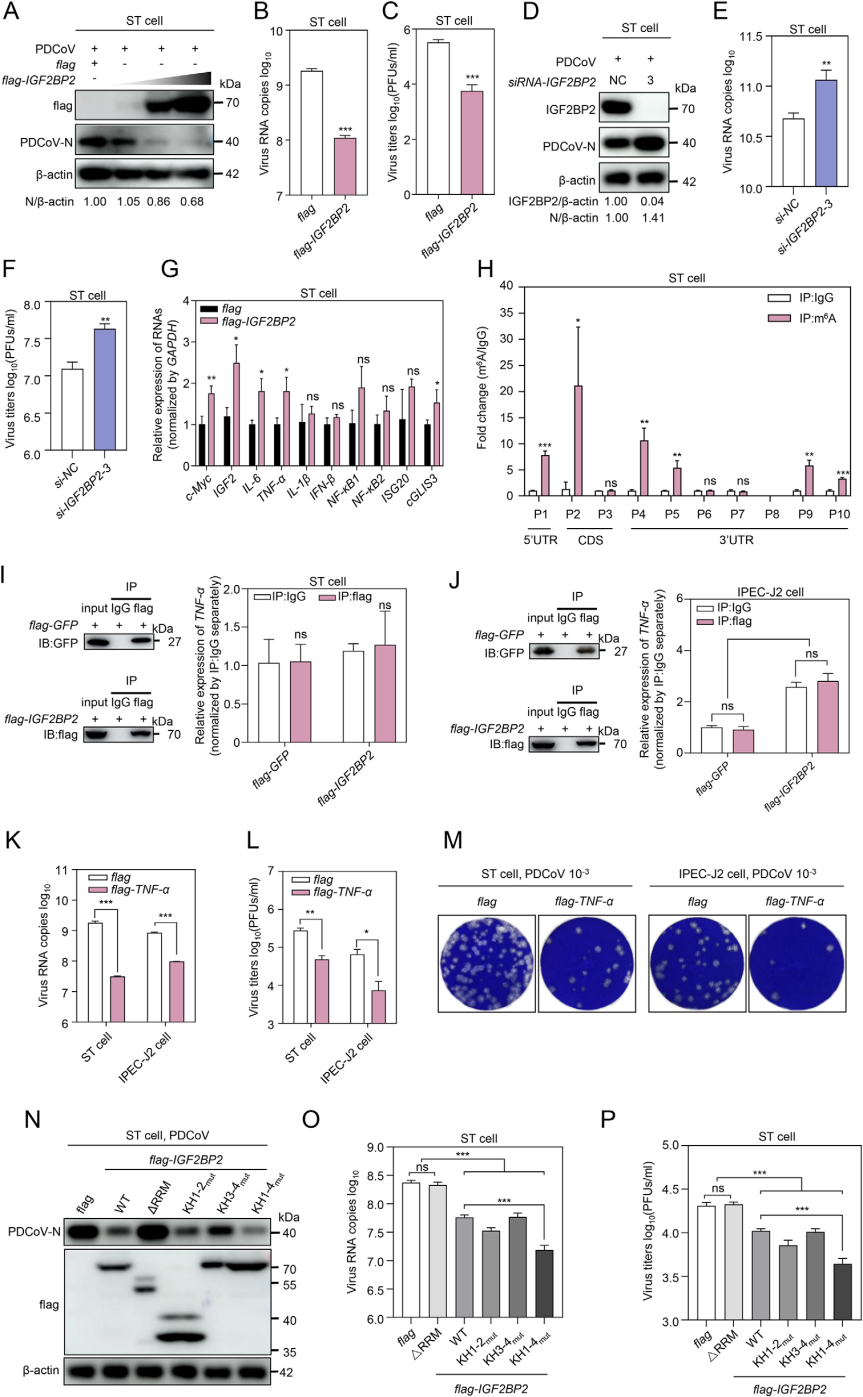
IGF2BP2 restrains PDCoV replication through upregulating TNF‐α expression independent on m^6^A modification. (A‐C) Overexpression of IGF2BP2 inhibited PDCoV replication. (D–F) Silencing IGF2BP2 enhanced PDCoV replication. IGF2BP2‐overexpressed or ‐silenced ST cells were infected by PDCoV at an MOI of 0.1 for 18 h. Virus replication was detected by western blot (A or D), absolute RT‐qPCR (B or E), and plaque assays (C or F), respectively. (G) IGF2BP2 overexpression upregulated the mRNA level of its regulated downstream cytokines and target genes. (H) m^6^A enrichment of *TNF‐α* mRNA putative sites in ST cells by MeRIP‐qPCR. Results are presented relative to those obtained with IgG. (I and J) RIP and RT‐qPCR experiments were performed to check *TNF‐α* mRNA association with IGF2BP2 in ST cells (I) or IPEC‐J2 cells (J). After transfection with *flag‐IGF2BP2* or *flag‐GFP* for 24 h, ST cells (I) or IPEC‐J2 cells (J) were lysed for IP and Western blot. IP complex was subjected to RT‐qPCR analysis. (K and L) TNF‐α inhibits PDCoV replication. ST cells or IPEC‐J2 cells with TNF‐α transfection were infected by PDCoV at an MOI of 0.1 for 18 h. Virus replication was detected by absolute RT‐qPCR (K) and plaque assays (L). (M) The representative plaque used for viral titer in (L). (N‐P) IGF2BP2 KH‐domain mutants inhibit PDCoV replication. ST cells were transfected with indicated IGF2BP2 mutants and infected by PDCoV at an MOI of 0.1 for another 18 h, separately. Virus replication was detected by western blot (N), absolute RT‐qPCR (O), and plaque assays (P). The protein bands shown in panel A and D were quantified using ImageJ software. Data are presented as mean ± SD (*n* = 3; *p < 0.05, **p < 0.01, ***p < 0.001; ns, no significant; two‐tailed unpaired *t*‐test).

Inhibition of METTL3 could attenuate inflammation via IGF2BP2‐dependent mechanisms [55]. We speculated whether IGF2BP2 antiviral roles involved in regulating the expression of proinflammatory cytokines. To test this possibility, we quantified the cytokine related transcripts following IGF2BP2 overexpression. RT‐qPCR analysis confirmed that mRNA levels of IL‐6 and TNF‐α were significantly upregulated, alongside known IGF2BP2 targets c‐MYC and IGF2 (Figure 8G) [38, 50, 56]. To ascertain whether IGF2BP2 affects *TNF‐α* mRNA stability in an m^6^A modification‐dependent manner, the DNA sequence of *TNF‐α* mRNA was analyzed for potential m^6^A modification motifs (Figure S7A). However, RT‐qPCR showed that IGF2BP2 had no effect on the stability of *TNF‐α* mRNA in porcine cells (Figure S7B). For further confirmation, a dual‐luciferase assay was performed (Figure S7C). Consistently, IGF2BP2 could not disturb the luciferase activity recombined with 5'UTR, CDS, or 3'UTR of *TNF‐α* mRNA (Figure S7D). MeRIP assay revealed that six of the predicted regions in *TNF‐α* mRNA, particularly region P1 (1–181 nt) in the 5'UTR, P2 (375–573 nt) in the CDS, and P4 (886–1068 nt) in the 3'UTR of *TNF‐α* mRNA were significantly enriched, confirming the presence of m^6^A marks in *TNF‐α* mRNA (Figure 8H and Figure S7E). RIP followed by RT‐qPCR confirmed that IGF2BP2 could not bind to *TNF‐α* mRNA (Figure 8I,J). Taken together, these data demonstrate that IGF2BP2 regulates *TNF‐α* expression independent on m^6^A modification.

Functional assays further demonstrated that TNF‐α inhibited PDCoV replication (Figure 8K–M), suggesting that IGF2BP2 exerts antiviral activity through upregulation of cytokine. To delineate the structural basis of IGF2BP2's antiviral activity, we employed the ΔRRM and KH‐mutant variants of IGF2BP2 to explore. The data exhibited in Figure 8N–P revealed that the RRM but not the KH mutant abolished the antiviral effects of IGF2BP2 on PDCoV replication, indicating that IGF2BP2's antiviral activity relies on its RRM‐dependent RNA binding function but not its KH‐domain binding to *cGLIS3*.

Collectively, these data indicated that IGF2BP2 upregulates TNF‐α expression independent of TNF‐α m^6^A modification to inhibits PDCoV replication.

### 2.9 *cGLIS3* Promotes Coronavirus Replication via Suppressing IGF2BP2‐Regulated TNF‐α Expression

Given that *cGLIS3‐*IGF2BP2 interaction promotes the K48‐linked ubiquitin degradation of IGF2BP2 and IGF2BP2 upregulates TNF‐α expression independent of TNF‐α m^6^A modification, we monitored the temporal dynamics of *cGLIS3*, *IGF2BP2* mRNA, and protein expression during PDCoV infection. Figure 9A showed that the level of *cGLIS3* or IGF2BP2 mRNA gradually increased, and the level of IGF2BP2 protein peaked at 18 hpi and declined sharply thereafter, coinciding with a surge in *cGLIS3* expression at later stages. This inverse correlation suggests *cGLIS3‐*driven degradation. Western blot analysis further confirmed that IGF2BP2 protein level was reduced in *cGLIS3* overexpressing IPEC‐J2 cells infected with PDCoV at 24 hpi, and elevated in *cGLIS3‐*silenced ST cells (Figure 9B,C). Consistently, knockdown of *cGLIS3* upregulated IL‐6 and TNF‐α mRNA levels, phenocopying the effect of IGF2BP2 overexpression (Figure 9D). Moreover, the wild‐type *cGLIS3* but not the m^6^A‐deficient *cGLIS3* could impair the upregulation of TNF‐α by IGF2BP2 (Figure 9E), further supporting the model that the m^6^A‐modified *cGLIS3* interaction with IGF2BP2 attenuates cytokine expression. To directly test whether *cGLIS3* antagonizes IGF2BP2's antiviral function, we overexpressed *cGLIS3* in IGF2BP2‐transfected cells. *cGLIS3* was able to restore virus replication in the presence of wild‐type IGF2BP2, but this effect was abolished when either *cGLIS3* lacked m^6^A sites or IGF2BP2 carried mutations in its KH‐domains (Figure 9F,G), confirming that *cGLIS3* promotes PDCoV replication by restraining the IGF2BP2‐mediated TNF‐α expression.

**FIGURE 9:**
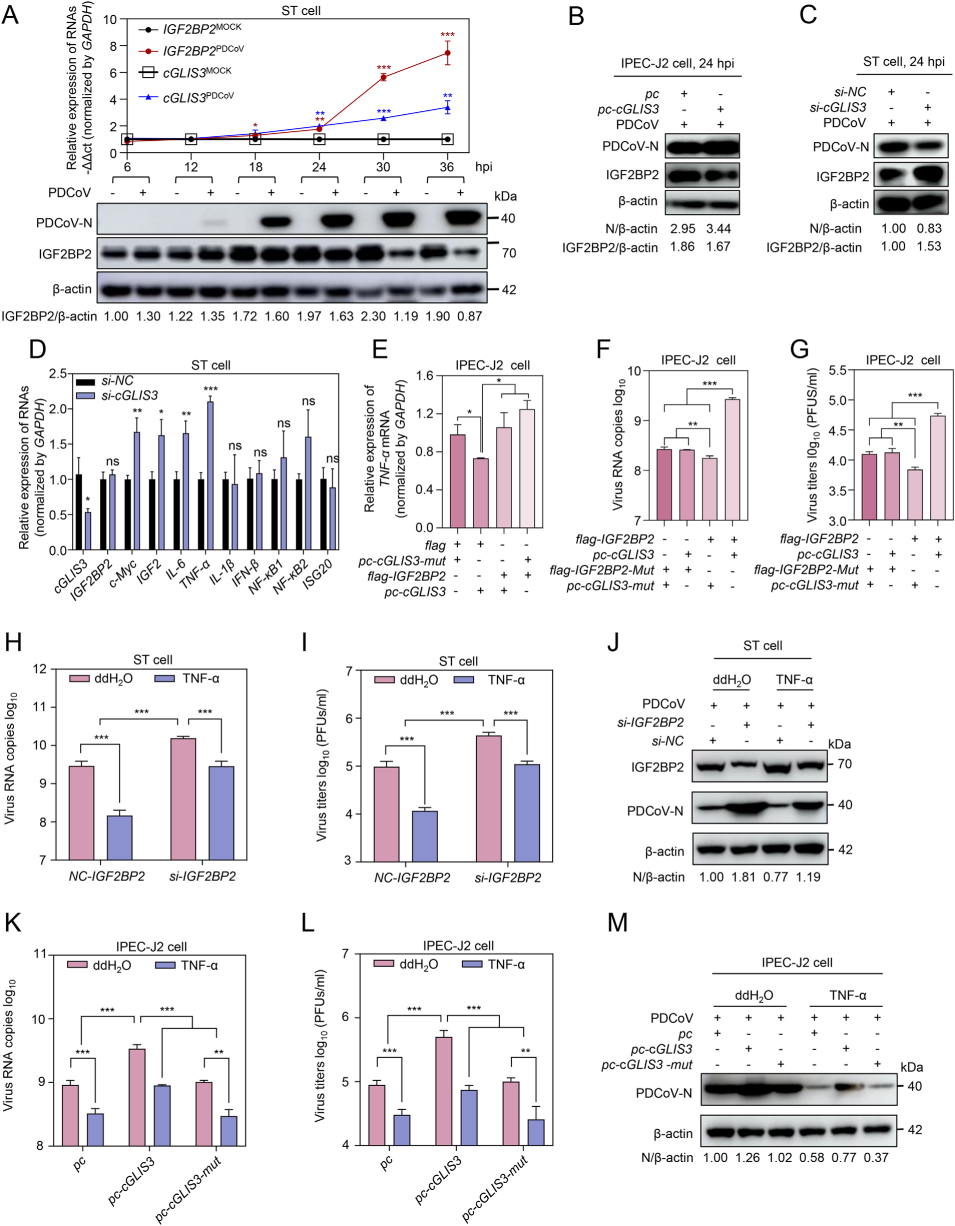
*cGLIS3*‐IGF2BP2 interaction facilitates PDCoV replication through restraining TNF‐α expression. (A) mRNA or protein level of IGF2BP2 and *cGLIS3* level with or without PDCoV infection in ST cells. (B and C) Protein levels of IGF2BP2 and PDCoV N in *cGLIS3*‐overexpressed IPEC‐J2 cells (B) or ‐silenced (C) ST cells infected by PDCoV at an MOI of 0.1 for another 24 h. (D) The mRNA level of potential downstream genes regulated by IGF2BP2 in ST cells with *cGLIS3* silencing examined by RT‐qPCR. (E) The mRNA level of *TNF‐α* in IPEC‐J2 cells co‐transfected with *pc‐cGLIS3* (or *pc‐cGLIS3‐mut*) and *flag‐IGF2BP2* (or *flag*) examined by RT‐qPCR. (F and G) *cGLIS3* overexpression rescues the suppression of IGF2BP2 in PDCoV replication. IPEC‐J2 cells were co‐transfected with *pc‐cGLIS3* (or *pc‐cGLIS3‐mut*) and *flag‐IGF2BP2* (or *flag‐IGF2BP2‐Mut*) and infected by PDCoV at an MOI of 0.1 for another 18 h. Virus titers were detected by absolute RT‐qPCR (F) and plaque assays (G). (H‐M) TNF‐α restored the suppression of PDCoV replication in IGF2BP2‐KD (H‐J) or *cGLIS3*‐overexpressed (K‐M) cells. The IGF2BP2‐silenced (or not) ST cells or *cGLIS3*‐overexpressed (or not) IPEC‐J2 cells were treated with TNF‐α (100 ng mL^−1^) for 12 h, and then infected with PDCoV at an MOI of 0.1 for another 15 h. Cells were then subjected to absolute RT‐qPCR of PDCoV *M* gene (H or K), plaque assays (I or L), and Western blot (J or M), respectively. The protein bands shown in panel A, B, C, J, and M were quantified using ImageJ software. Data are presented as mean ± SD (*n* = 3; *p < 0.05, **p < 0.01, ***p < 0.001; ns, no significant; two‐tailed unpaired *t*‐test).

To further understand the role of TNF in enhancing PDCoV replication through the *cGLIS3*‐IGF2BP2 interaction, we conducted the TNF‐α rescuing experiments. TNF‐α pre‐stimulation did not affect the cell viability (Figure S8A and E). However, TNF‐α pretreatment has a stronger antiviral activity in either ST cells (Figure S8B–D) or IPEC‐J2 cells (Figure S8F–H). Further TNF‐α rescue assays showed that the supplement of TNF‐α significantly restrained PDCoV replication in IGF2BP2‐deficient or *cGLIS3*‐overexpressed cells (Figure 9H–M), indicating *cGLIS3*‐IGF2BP2 interaction suppresses TNF‐α expression to facilitate PDCoV replication.

Collectively, these findings demonstrate that the m^6^A‐modified *cGLIS3* promotes PDCoV replication by binding to IGF2BP2 and establish a *cGLIS3‐*IGF2BP2 interaction‐regulated host response to coronavirus infection.

## 3 Discussion

Accumulating evidence indicates that viral infection perturbs host circRNA expression, [57, 58, 59, 60] yet mechanistic insight into how these circRNAs regulate viral replication has largely centered on miRNA sponging [25, 27]. In this study, using deltacoronavirus as an RNA virus model, we identify *cGLIS3* as a noncoding circRNA that modulates innate immunity via a m^6^A modification‐dependent *cGLIS3*‐IGF2BP2 interaction. Notably, deltacoronavirus enhances *cGLIS3* biogenesis to inhibit IGF2BP2‐induced innate immunity by a direct binding of viral N protein to *GLIS3* pre‐mRNA. These findings uncover a previously unrecognized circRNA‐centered regulatory mechanism engaged during coronavirus infection.

Host RBPs are known to shape circRNA biogenesis, primarily through post‐transcriptional regulation of splicing [20, 21, 22, 61]. Whether viral RBPs participate in this process has remained unclear. PDCoV N protein, a viral RBP, has been implicated in cytoplasmic antagonism of host antiviral pathways [62, 63]. Here, we demonstrate that N protein also enters the nucleus, binds directly to *GLIS3* pre‐mRNA, co‐localizes with the nuclear speckle marker SC35, and promotes *cGLIS3* production at both the transcriptional and post‐transcriptional levels (Figure 6 and Figure S5). These data provide direct evidence that a viral RBP can modulate host circRNA biogenesis, expanding our understanding of circRNA regulation beyond host‐driven processes.

So far, most studies of circRNA‐virus interaction focus on the role of miRNA sponges. Little is known about the role of circRNA‐RBP interaction in viral infection. Usually, circRNA interacts with RBP based on specific binding sites [17, 64], and m^6^A modification featured with “RRACH” motif is abundant in circRNAs [40, 65, 66]. Here, our experiments show that *cGLIS3* eludes RNase L‐mediated degradation and enhances ubiquitin‐dependent degradation of IGF2BP2 after *cGLIS3* interacting with m^6^A reader IGF2BP2, which facilitates deltacoronavirus replication via decreasing TNF‐α expression (Figure 4, Figure 7, and Figure 9). Interestingly, while SARS‐CoV‐2 or PDCoV infection is generally associated with strong induction of TNF‐α and IL‐6 [67, 68, 69, 70, 71, 72], our results uncover a circRNA‐dependent mechanism by which deltacoronavirus actively tunes TNF‐α output through the *cGLIS3*‐IGF2BP2 axis. This immune‐modulatory strategy likely enables the virus to balance antiviral signaling and replication efficiency.

IGF2BP2, as an m^6^A reader, plays a crucial role in *cGLIS3* metabolism from biogenesis to degradation based on its m^6^A recognition function. The KH‐domains of IGF2BP2 are responsible for the recognition and binding of mRNAs with m^6^A modification [38, 73]. Our observation demonstrates that the KH‐domain mutant of IGF2BP2 exhibits a significantly weakened effects on *cGLIS3* stabilization or biogenesis (Figure 4 and Figure 5). Compared with the role of IGF2BP2 in *cGLIS3* metabolism, YTHDFs or YTHDCs always plays a single role in circRNA stability [74], translation [45], circulation [75], or nucleocytoplasmic export [65], respectively. We attribute this difference to the difference of RNA recognition and binding domains between IGF2BP2 (KH) and YTHDFs or YTHDCs (YTH) as previous reported [38]. Notably, we notice that IGF2BP2 is predominately localized in the cytoplasm with a little distribution in the nucleus (Figure 5I and Figure S4F), suggesting that while nuclear IGF2BP2 is not abundant, it exerts essential and efficient roles in circRNA biogenesis. These findings enrich current understanding of host RBP regulation of circRNAs and highlight the importance of m^6^A in shaping circRNA metabolism.

Similar to deltacoronavirus N, N proteins from *Alphacoronavirus*, *Betacoronavirus and Gammacoronavirus* also localize to the nucleus [76] and contain extensive intrinsically disordered regions with RNA chaperone activity [77, 78], raising the possibility that other CoV N proteins likewise bind pre‐mRNAs to modulate circRNA biogenesis. Here, we find that LKR‐domain is essential for deltacoronavirus N protein to regulate *cGLIS3* back‐splicing and *GLIS3* transcription (Figure 6), which is consistent with previous studies that LKR‐domain of other CoV N protein containing an arginine‐serine (RS)‐rich region has a high affinity to RNA with a direct interaction [29, 30], and RS‐rich repeats have been reported to associate with alternative pre‐mRNA splicing and have a localization in nuclear spots [79, 80]. The RS‐rich region of CoV N proteins is always abundant with phosphorylation sites and is phosphorylated by SR protein kinases, which impacts RNA binding, packaging, viral assembly, and subcellular localization [81, 82, 83, 84]. Therefore, we raise an intriguing possibility that viral RBP with RS‐rich LKR‐domain and the nuclear localization may modulate the host RNA and circRNA biogenesis.

From the circRNA perspective, it is notable that the motifs “RRACH”, “GGACT” and “AAACT” are detected in human, mouse and porcine *cGLIS3* (Figure 3G). This observation implies that human or mouse *cGLIS3* may also bind to IGF2BP2 dependent on its m^6^A modification with unknown function except for reported miRNA sponging or protein encoding role [41, 42]. IGF2BP2 preferentially binds to the ‘UGGAC’ consensus sequence containing the ‘GGAC’ m^6^A core motif [38, 40], and all predicted sites in this study are mutant to disturb the interaction between IGF2BP2 and m^6^A‐modified *cGLIS3* (Figure 3E,H,I). Therefore, the accurate m^6^A modification motifs of *cGLIS3* recognized by IGF2BP2 remains further identification. Additionally, we notice that *cGLIS3* exhibits a predominate distribution in cytoplasm with a little intranuclear distribution (Figure 1E,F and Figure S1A,B) and interacts with IGF2BP2 (Figure 3), indicating the cytoplasmic localization‐mediated role may be the mainly function of *cGLIS3*. Besides, YTHDC1 promotes cytoplasmic export of m^6^A‐modified human *circNSUN2* [65], and IGF2BP1 binds directly to circular RNAs recruiting Ran‐GTP and exportin‐2 to export circRNAs [85]. These findings provide a probable direction for exploring the potential nucleocytoplasmic export of *cGLIS3* based on its m^6^A modification. Hopefully, future studies in biogenesis and functions of host circRNA would bring up clinical benefits controlling microbial infection.

### 3.1 Limitations of the Study

The discovery that the PDCoV N protein and its RS‐rich domain (aa 143–153) modulate *cGLIS3* back‐splicing substantially expands our understanding of host circRNA regulation during viral infection. However, the precise mechanisms by which this domain functions, how SR protein kinases act on this region, and whether such regulatory roles are conserved among other microbial RBPs remain to be elucidated. These questions will require further investigation supported by genome‐wide sequencing. In addition, although IGF2BP2 and *cGLIS3* exhibit opposing effects on the expression of proinflammatory cytokines, the downstream molecular mechanisms and the possibility that *cGLIS3* modulates this process through additional pathways warrant further study.

## 4 Materials and Methods

### 4.1 Cell Culture and Virus

ST (CRL‐1746), Vero E6 (CRL‐1586), A549 (CRM‐CCL‐185), BHK‐21, HEK‐293T (CRL‐11268) cells and intestinal porcine epithelial cells (IPEC‐J2) were maintained in our laboratory. ST, Vero E6, A549, or BHK‐21 cells were maintained in Dulbecco's modified Eagle's medium (DMEM; Gibco, USA) supplemented with 10% heat‐inactivated fetal bovine serum (FBS; Gibco, USA). HEK‐293T cells were cultured in DMEM supplemented with 8% heat‐inactivated FBS (Biological Industries, Israel). IPEC‐J2 cells were maintained in Roswell Park Memorial Institute 1640 medium (RPMI‐1640; Gibco, USA) supplemented with 8% heat‐inactivated FBS (Biological Industries, Israel). All cell lines were cultured at 37°C in a humidified incubator containing 5% CO_2_.

The PDCoV strain CH‐HA3‐2017, TGEV, PEDV, SADS‐CoV, HCoV‐OC43, HCoV‐229E, AIV PR8 strain, rabies virus HEP‐Flury strain, Sendai virus (SeV) or herpes simplex virus (HSV) was maintained in our laboratory, respectively. For PDCoV infection, DMEM containing 0.2 mg mL^−1^ tosylsulfonyl phenylalanyl chloromethyl ketone (TPCK)‐treated trypsin (Sigma, USA) was used. ST or IPEC‐J2 cells were infected with PDCoV at the indicated multiplicities of infection (MOIs) and harvested at the indicated time points. PDCoV virions were concentrated by ultracentrifugation at 50 000 × *g* for 2 h using a Ty 50.2 rotor (Beckman Coulter, CA, USA) through a 15% (w/v) sucrose cushion. Viral pellets were resuspended overnight in 200 µL NTE buffer (50 mM Tris‐HCl, 150 mM NaCl, 15 mM CaCl_2_, pH 6.5). Viral genomic RNA was isolated from purified virions.

### 4.2 RNA Extraction

Tissues including heart, liver, spleen, lung, kidney, brain, duodenum, jejunum, ileum, cecum, colon, rectum, and tonsils, were collected from 3‐month‐old healthy pigs. Total RNA was extracted using a total RNA isolation reagent (Vazyme, China) according to the manufacturer's instructions and used for RT‐PCR and RT‐qPCR analyses. Nuclear and cytoplasmic RNAs were isolated using NE‐PER Nuclear and Cytoplasmic Extraction Reagents (Thermo Scientific, USA). RNA concentration and purity were determined using a NanoDrop One spectrophotometer (Thermo Scientific, USA).

### 4.3 Fluorescence In Situ Hybridization

Cy3‐labeled probes targeting *cGLIS3* were synthesized by RiboBio (Guangzhou, China). RNA fluorescence in situ hybridization was performed using a commercial kit (RiboBio, China) according to the manufacturer's instructions. For co‐localization analyses, cells were fixed, permeabilized, prehybridized, and hybridized, followed by incubation with primary antibodies (1:300 dilution in 5% skim milk) overnight at 4°C. After washing, cells were incubated with secondary antibodies (1:200 dilution) for 1 h at 37°C in the dark. Nuclei were counterstained with DAPI for 10 min at room temperature. Images were acquired using an LSM780 confocal microscope (Zeiss, Germany).

### 4.4 RT‐PCR and RT‐qPCR

The cDNA synthesis was performed using HiScript II Q RT SuperMix with genomic DNA eraser (Vazyme, China). Quantitative PCR was conducted using 2×FastAmpli SYBR qPCR Premix (Biori, China). Relative RNA expression levels were calculated using the 2^−ΔΔCt^ method, with *GAPDH* serving as the internal control. PDCoV *M* gene detection used the probe 5′‐FAM‐CACACCAGTCGTTAAGCATGGCAAGCT‐BHQ‐3′. Primer sequences were listed in Table S1.

### 4.5 Digested with RNase R

Total RNA (2 µg) was incubated with or without RNase R (3 U/µg; Epicentre Biotechnologies, USA) at 37°C for 20 min. RNA was subsequently purified by phenol‐chloroform extraction.

### 4.6 Plasmid Construction

Porcine *GLIS3 exon 3* with a 400 nt upstream and a 50 nt downstream flanking sequences was cloned into the *pcDNA3.1*(+) vector. A 200 nt upstream sequence was duplicated and inserted downstream in reverse orientation to generate *pc‐cGLIS3*. The *pc‐cGLIS3‐mut* construct was generated using synthetic mutant sequences. *pmirGLO‐cGLIS3‐full* and *pmirGLO‐cGLIS3‐target* constructs were generated by inserting corresponding miRNA sequences into the *pmirGLO* vector. *Flag‐poAGO2, flag‐GFP*, METTL3, METTL14, WTAP, *IRES^EMCV^* and *Rluc/Fluc* reporter vectors were previously described and stored in our laboratory [26]. IGF2BP2 and EIF4A3 constructs were cloned into the *pcaggs‐flag‐N* vector, and mutant constructs were generated accordingly. *pcDNA3.1‐ORF1/2* plasmids were constructed by inserting the predicted ORF sequences with an additional flag tag (ORF‐flag‐N/C‐terminal) into *pcDNA3.1(+)* plasmid, respectively. All plasmids were confirmed by Sanger sequencing. Primers for plasmid constructions were listed in Table S2.

### 4.7 Transfection of Plasmids and siRNAs

miRNA mimics, siRNAs targeting the back‐spliced junction of *cGLIS3*, and their nontargeting negative controls were synthesized by GenePharma (Shanghai, China). Plasmid DNA, siRNAs, and miRNA mimics (GenePharma, China) were transfected using Lipofectamine 3000 (Invitrogen, USA) following the manufacturer's instructions. Sequences were listed in Table S3.

### 4.8 Plaque Assay

ST cells were seeded in 12‐well plates and infected with PDCoV for 1 h at 37°C. Subsequently, Cells were overlaid with a mixture of DMEM and low melting point agarose ‌SeaPlaque Agarose (Lonza, Switzerland). At 48 h post‐infection (hpi), cells were fixed with 10% neutral buffered formaldehyde at room temperature for 30 min and stained by 5% crystal violet solution for 2 h to visualize plaques, and plaques were used to calculate the virus titer.

### 4.9 Western Blot

Total proteins were extracted using lysis buffer consisting of 2% sodium dodecyl sulfate (SDS), 1% Triton X‐100, 50 mM Tris‐HCl, and 150 mM NaCl (pH 7.5), and separated by 10% SDS‐PAGE, followed by transfer to a nitrocellulose membrane. Membrane was blocked with 5% skim milk and incubated with primary antibodies at 4°C overnight, followed by HRP‐conjugated secondary antibodies. Signals were detected using enhanced chemiluminescence (ECL) reagents (Bio‐Rad, Hercules, CA, USA), and a Quantity One system (Bio‐Rad, USA) was applied for analysis. The primary antibodies were anti‐β‐actin (Hua‐bio, China), anti‐IGF2BP2 (ABclonal, China), anti‐Flag (Abcam, USA), and anti‐PDCoV N protein (Medgene Labs, USA) were purchased from the indicated companies.

### 4.10 RNA Immunoprecipitation

For miRNA sponging verification, HEK‐293T cells were transfected with pc‐*cGLIS3* and *flag‐poAGO2* or *flag‐GFP* plasmids for 36 h. For *cGLIS3*‐IGF2BP2 interaction, HEK‐293T cells were transfected with *pc‐cGLIS3* (or *pc‐cGLIS3‐mut*) and flag‐IGF2BP2 (or flag‐IGF2BP2‐Mut) for 36 h. Cells were then pelleted and lysed with NP‐40 lysis buffer containing protease and RNase inhibitors. The lysates were incubated with Flag antibody (Huabio, China)‐coated beads or control IgG with rotation at 4°C overnight, respectively. Then, the RIP complex was concentrated, and the precipitated RNA was extracted and analyzed by RT‐qPCR. Primers were listed in Table S1.

### 4.11 Luciferase Reporter Assay

HEK‐293T cells were seeded in 24‐well plates and co‐transfected with a mixture of reporter constructs and miRNA mimics using jet PRIME (Polyplus, France) according to the manufacturer's instructions. In the IRES activity assay, cells were transfected with the empty or IRES‐inserted vector. Luciferase activity was measured at 36 h post‐transfection using a dual luciferase reporter assay system (Beyotime Biotechnology, China). The firefly luciferase (Fluc) activity was normalized with Renilla luciferase (Rluc) activity.

### 4.12 m^6^A Methylated RNA Immunoprecipitation‐qPCR (MeRIP‐qPCR)

Total RNA was extracted, DNase‐treated, fragmented, and subjected to immunoprecipitation using an m^6^A‐specific antibody (BersinBio, China). Enriched RNAs were quantified by RT‐qPCR. Primers were listed in Table S1.

### 4.13 Immunofluorescence

Cells were fixed with 4% (m/v) paraformaldehyde for 30 min at room temperature. Then cells were permeabilized with pre‐cooling PBS containing 1% Triton X‐100 for 15 min, and incubated with the primary antibodies overnight at 4°C. Then, cells were incubated with secondary antibodies for 1 h at 37°C protecting from light. Nuclei, were counterstained with DAPI for 10 min at room temperature. Finally, images were acquired using an LSM780 laser scanning confocal microscope (Zeiss, Oberkochen, Germany).

### 4.14 Chromatin Immunoprecipitation

The chromatin immunoprecipitation (ChIP) assay was perform using a ChIP Assay Kit (Beyotime Biotechnology, China), including cross‐linking, chromatin fragmentation, immunoprecipitation, reverse cross‐linking, and DNA purification. Enriched DNAs were then quantified by qPCR. Primers were listed in Table S1.

### 4.15 Animal Experiments

The animal experiments were conducted in accordance with procedures approved by the Animal Ethical and Welfare Committee for Institutional Animal Care and Use Committee (IACUC) of Zhejiang University (Approval No. ZJU20250758). Tissues including heart, liver, spleen, lung, kidney, brain, duodenum, jejunum, ileum, cecum, colon, rectum, and tonsils, were collected from 3 3‐month‐old healthy pigs. A 20 mg aliquot was lysed using a total RNA isolation reagent for circRNA‐level analysis.

### 4.16 Statistical Analysis

Statistical analyses were performed using GraphPad Prism 9.5.0. Data are presented as mean ± standard deviation (SD) at three biological replicates. Statistical significance was assessed using unpaired two‐tailed Student's *t*‐test. The *p* values*<* 0.05 are deemed statistically significant difference (*p < 0.05; **p < 0.01; ***p < 0.001; ns, no significant).

## Author Contributions

**J.Z**. and **J.G**. conceived, supervised the study and designed the experiments. **L. D**., **L. W**., **S. D**., **J. L**., **L. Z**., **Y. Y**., **H. L**., **W. D**., and **W. W**. contributed to experimental design, performed the experiments, and analyzed the data. **D. L**., **W.W**., **J.G**., and **J.Z**. contributed to data analysis and manuscript writing. All authors reviewed and edited the manuscript.

## Conflicts of Interest

The authors declare no conflicts of interest.

## Supporting information




**Supporting File 1**: advs76822‐sup‐0001‐SuppMat.docx.

**Supporting File 2**: advs76822‐sup‐0002‐FigureS1‐S8.pptx.

**Supporting File 3**: advs76822‐sup‐0001‐TableS1‐S3.pptx.

## Data Availability

RNA‐seq data set of circRNA and miRNAs have been deposited at Gene Expression Omnibus (https://www.ncbi.nlm.nih.gov/geo/) under the accession numbers GSE147188 and GSE176550.

## References

[1] J. Cui , F. Li , and Z. L. Shi , “Origin and Evolution of Pathogenic Coronaviruses,” Nature Reviews Microbiology 17 (2019): 181–192. 10.1038/s41579-018-0118-9 PMC7097006 30531947

[2] P. C. Woo , S. K. Lau , C. S. Lam , et al., “Discovery of Seven Novel Mammalian and avian Coronaviruses in the Genus Deltacoronavirus Supports Bat Coronaviruses as the Gene Source of Alphacoronavirus and Betacoronavirus and avian Coronaviruses as the Gene Source of Gammacoronavirus and Deltacoronavirus,” Journal of Virology 86 (2012): 3995–4008. 10.1128/JVI.06540-11 PMC3302495 22278237

[3] S. K. P. Lau , E. Y. M. Wong , C. C. Tsang , et al., “Discovery and Sequence Analysis of Four Deltacoronaviruses From Birds in the Middle East Reveal Interspecies Jumping With Recombination as a Potential Mechanism for Avian‐to‐Avian and Avian‐to‐Mammalian Transmission,” Journal of Virology 92 (2018): e00265‐18. 10.1128/JVI.00265-18 PMC6052312 29769348

[4] C. Duan , “An Updated Review of Porcine Deltacoronavirus in Terms of Prevalence, Pathogenicity, Pathogenesis and Antiviral Strategy,” Frontiers in Veterinary Science 8 (2021): 811187. 10.3389/fvets.2021.811187 PMC8792470 35097055

[5] L. Wang , B. Byrum , and Y. Zhang , “Detection and Genetic Characterization of Deltacoronavirus in Pigs,” Emerging Infectious Diseases 20 (2014): 1227–1230. 10.3201/eid2007.140296 PMC4073853 24964136

[6] K. Jung , H. Hu , and L. J. Saif , “Calves Are Susceptible to Infection with the Newly Emerged Porcine Deltacoronavirus, but Not With the Swine Enteric Alphacoronavirus, Porcine Epidemic Diarrhea Virus,” Archives of Virology 162 (2017): 2357–2362. 10.1007/s00705-017-3351-z PMC7086908 28374120

[7] P. A. Boley , M. A. Alhamo , G. Lossie , et al., “Porcine Deltacoronavirus Infection and Transmission in Poultry, United States,” Emerging Infectious Diseases 26 (2020): 255–265. 10.3201/eid2602.190346 PMC6986833 31961296

[8] H. Zhang , Q. Ding , J. Yuan , F. Han , Z. Wei , and H. Hu , “Susceptibility to Mice and Potential Evolutionary Characteristics of Porcine Deltacoronavirus,” Journal of Medical Virology 94 (2022): 5723–5738. 10.1002/jmv.28048 35927214

[9] J. A. Lednicky , M. S. Tagliamonte , S. K. White , et al., “Independent Infections of Porcine Deltacoronavirus Among Haitian Children,” Nature 600 (2021): 133–137. 10.1038/s41586-021-04111-z PMC8636265 34789872

[10] S. Memczak , M. Jens , A. Elefsinioti , et al., “Circular RNAs Are a Large Class of Animal RNAs with Regulatory Potency,” Nature 495 (2013): 333–338. 10.1038/nature11928 23446348

[11] W. R. Jeck and N. E. Sharpless , “Detecting and Characterizing Circular RNAs,” Nature Biotechnology 32 (2014): 453–461. 10.1038/nbt.2890 PMC4121655 24811520

[12] A. Rybak‐Wolf , C. Stottmeister , P. Glazar , et al., “Circular RNAs in the Mammalian Brain Are Highly Abundant, Conserved, and Dynamically Expressed,” Molecular Cell 58 (2015): 870–885. 10.1016/j.molcel.2015.03.027 25921068

[13] X. Li , J. L. Zhang , Y. N. Lei , et al., “Linking Circular Intronic RNA Degradation and Function in Transcription by RNase H1,” Science China Life Sciences 64 (2021): 1795–1809. 10.1007/s11427-021-1993-6 34453665

[14] D. M. Liang , D. C. Tatomer , Z. Luo , et al., “The Output of Protein‐Coding Genes Shifts to Circular RNAs When the Pre‐mRNA Processing Machinery Is Limiting,” Molecular Cell 68 (2017): 940–954. 10.1016/j.molcel.2017.10.034 PMC5728686 29174924

[15] S. H. Boo , M. K. Shin , H. J. Hwang , et al., “Circular RNAs Trigger Nonsense‐Mediated mRNA Decay,” Molecular Cell 84 (2024): 4862–4877. 10.1016/j.molcel.2024.11.022 39667933

[16] T. B. Hansen , T. I. Jensen , B. H. Clausen , et al., “Natural RNA Circles Function as Efficient microRNA Sponges,” Nature 495 (2013): 384–388. 10.1038/nature11993 23446346

[17] R. Ashwal‐Fluss , M. Meyer , N. R. Pamudurti , et al., “circRNA Biogenesis Competes with Pre‐mRNA Splicing,” Molecular Cell 56 (2014): 55–66. 10.1016/j.molcel.2014.08.019 25242144

[18] C. K. Chen , R. Cheng , J. Demeter , et al., “Structured Elements Drive Extensive Circular RNA Translation,” Molecular Cell 81 (2021): 4300–4318. 10.1016/j.molcel.2021.07.042 PMC8567535 34437836

[19] T. Aktaş , İ. Avşar Ilık , D. Maticzka , et al., “DHX9 Suppresses RNA Processing Defects Originating From the Alu Invasion of the Human Genome,” Nature 544 (2017): 115–119. 10.1038/nature21715 28355180

[20] L. Errichelli , S. Dini Modigliani , P. Laneve , et al., “FUS Affects Circular RNA Expression in Murine Embryonic Stem Cell‐Derived Motor Neurons,” Nature Communications 8 (2017): 14741. 10.1038/ncomms14741 PMC5379105 28358055

[21] S. J. Conn , K. A. Pillman , J. Toubia , et al., “The RNA Binding Protein Quaking Regulates Formation of circRNAs,” Cell 160 (2015): 1125–1134. 10.1016/j.cell.2015.02.014 25768908

[22] Q. Li , G. Yang , B. Ren , et al., “ZC3H14 Facilitates Backsplicing by Binding to Exon‐Intron Boundary and 3' UTR,” Molecular Cell 84 (2024): 4314–4333. 10.1016/j.molcel.2024.10.001 39461343

[23] C. X. Liu and L. L. Chen , “Circular RNAs: Characterization, Cellular Roles, and Applications,” Cell 185 (2022): 2016–2034. 10.1016/j.cell.2022.04.021 35584701

[24] J. Min , Y. Li , X. Li , et al., “The circRNA circVAMP3 Restricts Influenza A Virus Replication by Interfering with NP and NS1 Proteins,” PLOS Pathogens 19 (2023): 1011577. 10.1371/journal.ppat.1011577 PMC10441791 37603540

[25] Z. Qu , F. Meng , J. Shi , et al., “A Novel Intronic Circular RNA Antagonizes Influenza Virus by Absorbing a microRNA That Degrades CREBBP and Accelerating IFN‐β Production,” MBio 12 (2021): 0101721. 10.1128/mBio.01017-21 PMC8406138 34281396

[26] L. Du , X. Wang , J. Liu , et al., “A Previously Undiscovered Circular RNA, circTNFAIP3, and Its Role in Coronavirus Replication,” MBio 12 (2021): 0298421. 10.1128/mBio.02984-21 PMC8593679 34781747

[27] H. Li , L. Du , J. Li , et al., “A Previously Unidentified circRNA Inhibits Virus Replication by Regulating the miR‐24‐3p/KEAP1 Axis,” PLOS Pathogens 20 (2024): 1012712. 10.1371/journal.ppat.1012712 PMC11651552 39689152

[28] R. McBride , M. van Zyl , and B. C. Fielding , “The Coronavirus Nucleocapsid Is a Multifunctional Protein,” Viruses 6 (2014): 2991–3018. 10.3390/v6082991 PMC4147684 25105276

[29] G. W. Nelson , S. A. Stohlman , and S. M. Tahara , “High Affinity Interaction Between Nucleocapsid Protein and Leader/Intergenic Sequence of Mouse hepatitis Virus RNA,” Journal of General Virology 81 (2000): 181–188. 10.1099/0022-1317-81-1-181 10640556

[30] C. K. Chang , Y. L. Hsu , Y. H. Chang , et al., “Multiple Nucleic Acid Binding Sites and Intrinsic Disorder of Severe Acute Respiratory Syndrome Coronavirus Nucleocapsid Protein: Implications for Ribonucleocapsid Protein Packaging,” Journal of Virology 83 (2009): 2255–2264. 10.1128/JVI.02001-08 PMC2643731 19052082

[31] R. Desrosiers , K. Friderici , and F. Rottman , “Identification of Methylated Nucleosides in Messenger RNA From Novikoff Hepatoma Cells,” Proceedings of the National Academy of Sciences 71 (1974): 3971–3975. 10.1073/pnas.71.10.3971 PMC434308 4372599

[32] K. D. Meyer , Y. Saletore , P. Zumbo , O. Elemento , C. E. Mason , and S. R. Jaffrey , “Comprehensive Analysis of mRNA Methylation Reveals Enrichment in 3′ UTRs and near Stop Codons,” Cell 149 (2012): 1635–1646. 10.1016/j.cell.2012.05.003 PMC3383396 22608085

[33] D. Dominissini , S. Moshitch‐Moshkovitz , S. Schwartz , et al., “Topology of the Human and Mouse m^6^A RNA Methylomes Revealed by m^6^A‐Seq,” Nature 485 (2012): 201–206. 10.1038/nature11112 22575960

[34] J. Liu , Y. Yue , D. Han , et al., “A METTL3–METTL14 Complex Mediates Mammalian Nuclear RNA *N* ^6^‐Adenosine Methylation,” Nature Chemical Biology 10 (2014): 93–95. 10.1038/nchembio.1432 PMC3911877 24316715

[35] G. Q. Zheng , J. A. Dahl , Y. M. Niu , et al., “ALKBH5 Is a Mammalian RNA Demethylase That Impacts RNA Metabolism and Mouse Fertility,” Molecular Cell 49 (2013): 18–29. 10.1016/j.molcel.2012.10.015 PMC3646334 23177736

[36] G. F. Jia , Y. Fu , X. Zhao , et al., “N6‐Methyladenosine in Nuclear RNA Is a Major Substrate of the Obesity‐Associated FTO,” Nature Chemical Biology 7 (2011): 885–887. 10.1038/nchembio.687 PMC3218240 22002720

[37] H. L. Shi , J. B. Wei , and C. He , “Where, When, and How: Context‐Dependent Functions of RNA Methylation Writers, Readers, and Erasers,” Molecular Cell 74 (2019): 640–650. 10.1016/j.molcel.2019.04.025 PMC6527355 31100245

[38] H. Huang , H. Weng , W. Sun , et al., “Recognition of RNA *N* ^6^‐Methyladenosine by IGF2BP Proteins Enhances mRNA Stability and Translation,” Nature Cell Biology 20 (2018): 285–295. 10.1038/s41556-018-0045-z PMC5826585 29476152

[39] X. J. Pan , B. Huang , Q. Ma , et al., “Circular RNA Circ‐TNPO_3_ Inhibits Clear Cell Renal Cell Carcinoma Metastasis by Binding to IGF_2_BP_2_ and Destabilizing SERPINH1 mRNA,” Clinical and Translational Medicine 12 (2022): 994. 10.1002/ctm2.994 PMC9309750 35876041

[40] B. T. Li , L. L. Zhu , C. L. Lu , et al., “circNDUFB2 Inhibits Non‐Small Cell Lung Cancer Progression via Destabilizing IGF2BPs and Activating Anti‐Tumor Immunity,” Nature Communications 12 (2021): 295. 10.1038/s41467-020-20527-z PMC7804955 33436560

[41] L. Xiong , Y. Gong , H. Liu , et al., “circGlis3 promotes beta‐cell dysfunction by binding to heterogeneous nuclear ribonucleoprotein F and encoding Glis3‐348aa protein,” iScience 27 (2024): 108680. 10.1016/j.isci.2023.108680 PMC10788204 38226164

[42] Y. Liu , Y. Yang , C. Xu , et al., “Circular RNA circGlis3 Protects Against Islet β‐Cell Dysfunction and Apoptosis in Obesity,” Nature Communications 14 (2023): 351. 10.1038/s41467-023-35998-z PMC9867769 36681689

[43] I. Legnini , G. Di Timoteo , F. Rossi , et al., “Circ‐ZNF609 Is a Circular RNA That Can Be Translated and Functions in Myogenesis,” Molecular Cell 66 (2017): 22–37. 10.1016/j.molcel.2017.02.017 PMC5387670 28344082

[44] N. R. Pamudurti , O. Bartok , M. Jens , et al., “Translation of CircRNAs,” Molecular Cell 66 (2017): 9–21. 10.1016/j.molcel.2017.02.021 PMC5387669 28344080

[45] Y. Yang , X. Fan , M. Mao , et al., “Extensive Translation of Circular RNAs Driven by *N* ^6^‐Methyladenosine,” Cell Research 27 (2017): 626–641. 10.1038/cr.2017.31 PMC5520850 28281539

[46] J. Zhao , J. Wu , T. Y. Xu , Q. C. Yang , J. H. He , and X. F. Song , “IRESfinder: Identifying RNA Internal Ribosome Entry Site in Eukaryotic Cell Using Framed k‐mer Features,” Journal of Genetics and Genomics 45 (2018): 403–406. 10.1016/j.jgg.2018.07.006 30054216

[47] Q. Zheng , C. Bao , W. Guo , et al., “Circular RNA Profiling Reveals an Abundant circHIPK3 That Regulates Cell Growth by Sponging Multiple miRNAs,” Nature communications 7 (2016): 11215. 10.1038/ncomms11215 PMC4823868 27050392

[48] C. X. Liu , X. Li , F. Nan , et al., “Structure and Degradation of Circular RNAs Regulate PKR Activation in Innate Immunity,” Cell 177 (2019): 865–880. 10.1016/j.cell.2019.03.046 31031002

[49] Y. Zhou , P. Zeng , Y. H. Li , Z. D. Zhang , and Q. H. Cui , “SRAMP: Prediction of Mammalian *N* ^6^ ‐Methyladenosine (m^6^A) Sites Based on Sequence‐Derived Features,” Nucleic Acids Research 44 (2016): 91. 10.1093/nar/gkw104 PMC4889921 26896799

[50] H. Weng , F. Huang , Z. Yu , et al., “The m^6^A Reader IGF2BP2 Regulates Glutamine Metabolism and Represents a Therapeutic Target in Acute Myeloid Leukemia,” Cancer Cell 40 (2022): 1566–1582. 10.1016/j.ccell.2022.10.004 PMC9772162 36306790

[51] H. Huang , E. Zeqiraj , B. H. Dong , et al., “Dimeric Structure of Pseudokinase RNase L Bound to 2‐5A Reveals a Basis for Interferon‐Induced Antiviral Activity,” Molecular Cell 53 (2014): 221–234. 10.1016/j.molcel.2013.12.025 PMC3974923 24462203

[52] S. Knudsen , “Promoter2.0: For the Recognition of PolII Promoter Sequences,” Bioinformatics 15 (1999): 356–361. 10.1093/bioinformatics/15.5.356 10366655

[53] K. R. Hurst , C. A. Koetzner , and P. S. Masters , “Identification of in Vivo‐Interacting Domains of the Murine Coronavirus Nucleocapsid Protein,” Journal of Virology 83 (2009): 7221–7234. 10.1128/JVI.00440-09 PMC2704785 19420077

[54] T. Xia , J. Y. Zhao , Z. Y. Zhang , et al., “The Deubiquitinase USP9X and E3 Ligase WWP1 Orchestrate IGF2BP2 Ubiquitination Homeostasis to Drive TNBC Progression and Cisplatin Sensitivity,” Cell Death & Disease 16 (2025): 703. 10.1038/s41419-025-08038-5 PMC12500958 41053085

[55] J. N. Wang , F. Wang , J. Ke , et al., “Inhibition of METTL3 Attenuates Renal Injury and Inflammation by Alleviating TAB3 m^6^A Modifications via IGF2BP2‐Dependent Mechanisms,” Science Translational Medicine 14 (2022): abk2709. 10.1126/scitranslmed.abk2709 35417191

[56] N. Dai , J. Rapley , M. Angel , M. F. Yanik , M. D. Blower , and J. Avruch , “mTOR Phosphorylates IMP2 to Promote IGF2 mRNA Translation by Internal Ribosomal Entry,” Genes & Development 25 (2011): 1159–1172. 10.1101/gad.2042311 PMC3110954 21576258

[57] X. Liu , S. Wei , S. Deng , et al., “Genome‐Wide Identification and Comparison of mRNA s, lnc RNA s and circ RNA s in Porcine Intramuscular, Subcutaneous, Retroperitoneal and Mesenteric Adipose Tissues,” Animal Genetics 50 (2019): 228–241. 10.1111/age.12781 30982992

[58] C. Li , X. Li , X. Hou , et al., “Comprehensive Analysis of circRNAs Expression Profiles in Different Periods of MDBK Cells Infected with Bovine Viral Diarrhea Virus,” Research in Veterinary Science 125 (2019): 52–60. 10.1016/j.rvsc.2019.05.005 31146221

[59] J. Chen , H. Wang , L. Jin , et al., “Profile Analysis of circRNAs Induced by Porcine Endemic Diarrhea Virus Infection in Porcine Intestinal Epithelial Cells,” Virology 527 (2019): 169–179. 10.1016/j.virol.2018.11.014 PMC7112103 30530223

[60] X. Ma , X. Zhao , Z. Zhang , et al., “Differentially Expressed Non‐Coding RNAs Induced by Transmissible Gastroenteritis Virus Potentially Regulate Inflammation and NF‐κB Pathway in Porcine Intestinal Epithelial Cell Line,” BMC Genomics 19 (2018): 747. 10.1186/s12864-018-5128-5 PMC6186045 30314467

[61] T. Aktas , I. Avsar Ilik , D. Maticzka , et al., “DHX9 Suppresses RNA Processing Defects Originating From the *Alu* Invasion of the Human Genome,” Nature 544 (2017): 115–119. 10.1038/nature21715 28355180

[62] Y. T. Hu , C. L. Hao , D. H. Wang , et al., “Porcine Deltacoronavirus Nucleocapsid Protein Antagonizes JAK‐STAT Signaling Pathway by Targeting STAT1 Through KPNA2 Degradation,” Journal of Virology 98 (2024): jvi00334. 10.1128/jvi.00334-24 PMC11264599 38829137

[63] S. Lee and C. Lee , “Functional Characterization and Proteomic Analysis of the Nucleocapsid Protein of Porcine Deltacoronavirus,” Virus Research 208 (2015): 136–145. 10.1016/j.virusres.2015.06.013 PMC7114568 26103099

[64] W. W. Du , L. Fang , W. Yang , et al., “Induction of Tumor Apoptosis Through a Circular RNA Enhancing Foxo3 Activity,” Cell Death & Differentiation 24 (2017): 357–370. 10.1038/cdd.2016.133 PMC5299715 27886165

[65] R. X. Chen , X. Chen , L. P. Xia , et al., “ *N* ^6^‐Methyladenosine Modification of circNSUN2 Facilitates Cytoplasmic Export and Stabilizes HMGA2 to Promote Colorectal Liver Metastasis,” Nature Communications 10 (2019): 4695. 10.1038/s41467-019-12651-2 PMC6795808 31619685

[66] Y. G. Chen , R. Chen , S. Ahmad , et al., “N(6)‐Methyladenosine Modification Controls Circular RNA Immunity,” Molecular Cell 76 (2019): 96–109. 10.1016/j.molcel.2019.07.016 PMC6778039 31474572

[67] J. S. Y. Ho , B. W. Y. Mok , L. Campisi , et al., “TOP1 Inhibition Therapy Protects Against SARS‐CoV‐2‐Induced Lethal Inflammation,” Cell 184 (2021): 2618–2632. 10.1016/j.cell.2021.03.051 PMC8008343 33836156

[68] M. Merad and J. C. Martin , “Pathological Inflammation in Patients with COVID‐19: A Key Role for Monocytes and Macrophages,” Nature Reviews Immunology 20 (2020): 355–362. 10.1038/s41577-020-0331-4 PMC7201395 32376901

[69] C. L. Huang , Y. M. Wang , X. W. Li , et al., “Clinical Features of Patients Infected with 2019 Novel Coronavirus in Wuhan, China,” Lancet 395 (2020): 497–506. 10.1016/S0140-6736(20)30183-5 PMC7159299 31986264

[70] H. L. Zhang , F. F. Han , X. L. Shu , et al., “Co‐Infection of Porcine Epidemic Diarrhoea Virus and Porcine Deltacoronavirus Enhances the Disease Severity in Piglets,” Transboundary and Emerging Diseases 69 (2022): 1715–1726. 10.1111/tbed.14144 33960702

[71] Y. Wu , Z. R. Shi , J. F. Chen , et al., “Porcine Deltacoronavirus E Protein Induces Interleukin‐8 Production via NF‐κB and AP‐1 Activation,” Veterinary Microbiology 274 (2022): 109553. 10.1016/j.vetmic.2022.109553 PMC9428115 36181744

[72] X. R. Zhou , X. N. Ge , Y. N. Zhang , et al., “Attenuation of Porcine Deltacoronavirus Disease Severity by Porcine Reproductive and Respiratory Syndrome Virus Coinfection in a Weaning Pig Model,” Virulence 12 (2021): 1011–1021. 10.1080/21505594.2021.1908742 PMC8023240 33797313

[73] J. Nielsen , M. A. Kristensen , M. Willemoës , F. C. Nielsen , and J. Christiansen , “Sequential Dimerization of Human Zipcode‐Binding Protein IMP1 on RNA: A Cooperative Mechanism Providing RNP Stability,” Nucleic Acids Research 32 (2004): 4368–4376. 10.1093/nar/gkh754 PMC514376 15314207

[74] Y. H. Chen , Z. N. Ling , X. L. Cai , Y. F. Xu , Z. Lv , and D. G. Zhang , “Activation of YAP1 by N6‐Methyladenosine–Modified circCPSF6 Drives Malignancy in Hepatocellular Carcinoma,” Cancer Research 82 (2022): 599–614. 10.1158/0008-5472.CAN-21-1628 34916222

[75] G. Di Timoteo , D. Dattilo , A. Centrón‐Broco , et al., “Modulation of circRNA Metabolism by M(6)A Modification,” Cell reports 31 (2020): 107641. 10.1016/j.celrep.2020.107641 32402287

[76] D. Shi , M. J. Lv , J. F. Chen , et al., “Molecular Characterizations of Subcellular Localization Signals in the Nucleocapsid Protein of Porcine Epidemic Diarrhea Virus,” Viruses 6 (2014): 1253–1273. 10.3390/v6031253 PMC3970149 24632575

[77] S. Zúñiga , J. L. G. Cruz , I. Sola , P. A. Mateos‐Gómez , L. Palacio , and L. Enjuanes , “Coronavirus Nucleocapsid Protein Facilitates Template Switching and Is Required for Efficient Transcription,” Journal of Virology 84 (2010): 2169–2175. 10.1128/JVI.02011-09 PMC2812394 19955314

[78] S. Zúñiga , I. Sola , J. L. Moreno , P. Sabella , J. Plana‐Durán , and L. Enjuanes , “Coronavirus Nucleocapsid Protein Is an RNA Chaperone,” Virology 357 (2007): 215–227. 10.1016/j.virol.2006.07.046 PMC7111943 16979208

[79] X. L. Jin , “Regulatory Network of Serine/Arginine‐Rich (SR) Proteins: The Molecular Mechanism and Physiological Function in Plants,” International Journal of Molecular Sciences 23 (2022): 10147. 10.3390/ijms231710147 PMC9456285 36077545

[80] J. A. Greig , T. A. Nguyen , M. Lee , et al., “Arginine‐Enriched Mixed‐Charge Domains Provide Cohesion for Nuclear Speckle Condensation,” Molecular Cell 77 (2020): 1237–1250. 10.1016/j.molcel.2020.01.025 PMC10715173 32048997

[81] T. Y. Peng , K. R. Lee , and W. Y. Tarn , “Phosphorylation of the Arginine/Serine Dipeptide‐Rich Motif of the Severe Acute Respiratory Syndrome Coronavirus Nucleocapsid Protein Modulates Its Multimerization, Translation Inhibitory Activity and Cellular Localization,” The FEBS Journal 275 (2008): 4152–4163. 10.1111/j.1742-4658.2008.06564.x PMC7164085 18631359

[82] B. A. Johnson , Y. Y. Zhou , K. G. Lokugamage , et al., “Nucleocapsid Mutations in SARS‐CoV‐2 Augment Replication and Pathogenesis,” PLOS Pathogens 18 (2022): 1010627. 10.1371/journal.ppat.1010627 PMC9275689 35728038

[83] S. Lu , Q. Z. Ye , D. Singh , et al., “The SARS‐CoV‐2 Nucleocapsid Phosphoprotein Forms Mutually Exclusive Condensates with RNA and the Membrane‐Associated M Protein,” Nature Communications 12 (2021): 502. 10.1038/s41467-020-20768-y PMC7820290 33479198

[84] C. R. Carlson , J. B. Asfaha , C. M. Ghent , et al., “Phosphoregulation of Phase Separation by the SARS‐CoV‐2 N Protein Suggests a Biophysical Basis for its Dual Functions,” Molecular Cell 80 (2020): 1092–1103. 10.1016/j.molcel.2020.11.025 PMC7677695 33248025

[85] L. H. Ngo , A. G. Bert , B. K. Dredge , et al., “Nuclear Export of Circular RNA,” Nature 627 (2024): 212–220. 10.1038/s41586-024-07060-5 38355801

