# Supplementary material for: Deltacoronavirus Modulates circRNA cGLIS3 Metabolism to Evade Host Antiviral Response
Source: Adv Sci (Weinh). 2026 Jul 27;13(59):e76822. doi: 10.1002/advs.76822 (PMC13403727; doi:10.1002/advs.76822)
Supplement: Supplementary file 1 [file ADVS-13-e76822-s002.docx]

**Supplemental figure legends**

**Figure S1.** **The molecular characterization and sequence annotation of porcine *cGLIS3***. (A) RT-qPCR analysis of *cGLIS3* abundance in the cytoplasm and nucleus of IPEC-J cells. (B) RT-qPCR analysis of *cGLIS3* abundance in the cytoplasm and nucleus of *pc-cGLIS3*-transfected HEK-293T cells. HEK-293T cells were transfected with *pc-cGLIS3* for 24 h, and the nuclear/cytoplasmic fractionation was then separated for RT-qPCR analysis. *GAPDH* and *U6* mRNAs served as cytoplasmic and nuclear controls, respectively. (C) The complete DNA sequence of *cGLIS3* showing the potential IRES^905-184^ and BSJ-spanning ORF. These marked with blue and underlined fragments are the potential IRES^905-184^. The fragments “^185^ATGXXXX····XXXXTGA^18^” are the potential *cGLIS3* BSJ-spanning ORF. (D) Schematic representation of the construction of *cGLIS3* overexpression plasmids. (E) RT-qPCR analysis of *cGLIS3* abundance in *pc-cGLIS3*-transfected HEK-293T cells. (F) The joined DNA sequence of *cGLIS3* BSJ-spanning ORF**.** These marked with yellow in bold fragments are the inserted *FLAG* tag of *pcDNA3.1-ORF1* or *pcDNA3.1-ORF2*, respectively. Data are presented as mean ± SD (n = 3; **p* < 0.05, ***p* < 0.01, ****p* < 0.001; ns, no significant; two-tailed unpaired *t*-test).

**Figure S2. *cGLIS3* could not absorb miRNA.** (A and B) RIP and RT-qPCR experiments were performed in HEK-293T cells. After co-transfection with *pc-cGLIS3* and *flag-poAGO2* (or *flag-GFP*) for 24 h, HEK-293T cells were lysed for IP and Western blot (A). IP complex was subjected to RT-qPCR analysis (B). (C) The *cGLIS3*-absorbed potential miRNAs predicted by *miRanda* and *RNAhybrid*. (D) Luciferase activity of HEK-293T cells after co-transfection with *pmirGLO-cGLIS3* (*cGLIS3-FL*) and miRNA mimics, respectively. (E and F) *cGLIS3* mutant or wild-type (WT) reporter vector construction. The predicted binding sites were mutated in *cGLIS3-FL* or *cGLIS3-878* (*cGLIS3-578*) as indicated in blue. (G-J) Luciferase activities of WT or mutant reporter plasmid in HEK-293T cells transfected with *novel_878* mimics (G and H) or *novel_578* mimics (I and J). Firefly luciferase activity in (D, G-J) was normalized to Renilla luciferase activity, respectively. Data are presented as mean ± SD (n = 3; **p* < 0.05, ***p* < 0.01, ****p* < 0.001; ns, no significant; two-tailed unpaired *t*-test).

**Figure S3. *cGLIS3* interacts with multiple RBPs.** (A) The DNA sequence of *cGLIS3* showing the potential binding sites of MBNL1, FUS, and IGF2BP2. These marked with green/blue/red fragments are MBNL1/FUS/IGF2BP2 potential binding sites, respectively. (B) The combination of *cGLIS3* with MBNL1 or FUS examined by RIP. After co-transfection with *flag-MBNL1* (or *flag-FUS*) and *pc-cGLIS3* for 24 h, HEK-293T cells were lysed for IP and Western blot. IP complex was subjected to RT-qPCR analysis. (C) A comparison of the three RBPs binding to *cGLIS3*. The compared data are from Figure 3C and Figure S3B, respectively. (D and E) The verification of *cGLIS3*-IGF2BP2 interaction during PDCoV infection. After infected by PDCoV at an MOI of 0.1 for 15 h, ST (D) or IPEC-J2 cells (E) were lysed for IP and Western blot. IP complex was subjected to RT-qPCR analysis. (F) RT-qPCR analysis of *cGLIS3* abundance in *pc-cGLIS3-* or *pc-cGLIS3*-*mut*-transfected HEK-293T cells. (G) Structural diagram of RNA binding domains of IGF2BP2 and its RNA binding domain-deficient mutants used in this study. Data are presented as mean ± SD (n = 3; **p* < 0.05, ***p* < 0.01, ****p* < 0.001; ns, no significant; two-tailed unpaired *t*-test).

**Figure S4. The effects of IGF2BP2 on *cGLIS3* biogenesis.** (A) Schematic diagram of *pc-cGLIS3-flanking*. (B) A dual-luciferase reporter assay determined the activity of *CMV* promoter in HEK-293T cells co-transfected with *pGL3-CMV* and indicated RBP plasmids. Firefly luciferase activity was normalized to Renilla luciferase activity. (C) The DNA sequence of *pc-cGLIS3-flanking* showing IGF2BP2 potential binding sites in *GLIS3* pre-mRNA. This blue labelled fragment is *cGLIS3* linear sequence. These marked with red and numbered alongside fragments as ^n^XXXX^n^ are IGF2BP2 potential binding sites. (D) Schematic diagram of *pc-circTNFAIP3-flanking*. (E) RT-qPCR analysis of *circTNFAIP3* in HEK-293T cells co-transfected with *pc-circTNFAIP3-flanking* and *flag-IGF2BP2* or control vector, separately. (F) Representative immunofluorescence co-staining of IGF2BP2 and a nuclear speckle marker SC35. Scale bars, 10 μm. (G) Schematic diagram of *pc-GLIS3-mini*. (H) The observed splicing isoforms verified by sequencing. The splicing junction was as shown by the arrow, respectively. (I and J) The *cGLIS3* abundance in normal or IGF2BP2-KD ST cells supplemented with *flag-pcaggs*, wild-type or KH-mutant IGF2BP2, respectively. After transfected with *flag-pcaggs*, *flag-IGF2BP2*, or *flag-IGF2BP2-Mut* for 24 h, ST cells (I) or IGF2BP2-KD (J) ST cells were treated with PDCoV at an MOI of 0.1 for another 15 h, respectively. Data are presented as mean ± SD (n = 3; **p* < 0.05, ***p* < 0.01, ****p* < 0.001; ns, no significant; two-tailed unpaired *t*-test).

**Figure S5. PDCoV N protein contributes to *cGLIS3* biogenesis.** (A and B) RT-qPCR measured the effects of different Pvps on abundance of *cGLIS3* (A) or *GLIS3* mRNA (B) in ST cells. (C) RT-qPCR analysis of *cGLIS3* level in HEK-293T cells transfected with *pc-cGLIS3-flanking* and indicated Pvps, respectively. (D) Schematic of different primers designed for identifying the effects of PDCoV N protein on *cGLIS3* back-splicing. (E) The combination of *GLIS3* pre-mRNA with PDCoV N protein examined by RIP. After co-transfection with *flag-PDCoV-N* (or *flag-GFP*) and *pc-cGLIS3-flanking* for 24 h, HEK-293T cells were lysed for IP and Western blot. (F) Representative immunofluorescence co-staining of PDCoV N protein and a nuclear speckle marker SC35. Scale bars, 10 μm. (G) Immunoblot analysis of HEK-293T cells transfected with flag-tagged PDCoV N protein 1-343 aa truncations. (H) Representative immunofluorescence co-staining of PDCoV N protein LKR truncations and a nuclear speckle marker SC35. Fluorescence intensity profiles of the red and green fluorescent signals along two different colored cross-section lines of an enlarged merged image are shown in the right panel. Scale bars, 10 μm. (I) Immunoblot analysis of HEK-293T cells transfected with LKR truncations of PDCoV N protein. (J and K) RT-qPCR measured the effects of PDCoV N protein LKR truncations on *cGLIS3* (J) and *GLIS3* mRNA (K) level in ST cells. (L) The combination of *GLIS3* pre-mRNA with PDCoV N protein LKR truncations by RIP. After co-transfection with *flag-PDCoV-N* LKR truncations (or *flag-GFP*) and *pc-cGLIS3-flanking* for 24 h, HEK-293T cells were lysed for IP and Western blot. (M) The combination of *GLIS3* pre-mRNA with PDCoV N protein 143-153 aa insertions by RIP. After co-transfection with *flag-PDCoV-N* (or *flag-GFP*) 143-153 aa insertions and *pc-cGLIS3-flanking* for 24 h, HEK-293T cells were lysed for IP and Western blot. Data are presented as mean ± SD (n = 3; **p* < 0.05, ***p* < 0.01, ****p* < 0.001; ns, no significant; two-tailed unpaired *t*-test).

**Figure S6. *cGLIS3* promotes ubiquitination-proteasome mediated IGF2BP2 degradation.** (A) The long exposure in the DMSO panel of Figure 7J. (B) Expression level of IGF2BP2 in IPEC-J2 cells co-transfected with *pc/pc-cGLIS3/pc-cGLIS3-mut* and *Ub-WT*/*Ub-Lys48R*, respectively. (C) Expression level of IGF2BP2 in ST cells co-transfected with *NC-cGLIS3/si-cGLIS3* and *Ub-WT*/*Ub-Lys48R*, respectively. The protein bands shown in panel B and C were quantified using ImageJ software.

**Figure S7. IGF2BP2 has no effect on TNF-*α* mRNA stability.** (A) The DNA sequence of *TNF-α* mRNA showing IGF2BP2 potential binding sites. The red fragments are IGF2BP2 potential motifs. (B) RT-qPCR analysis of the effect of IGF2BP2 or *cGLIS3* on *TNF-α* mRNA stability in porcine cells. The IGF2BP2-overexpressed or *cGLIS3*-silenced ST cells, and IGF2BP2- or *cGLIS3*-overexpressed IPEC-J2 cells were treated with ACTD (1 mg/ml) for 12 h, respectively. (C) Schematic representation of the dual-luciferase reporter constructions. (D) A dual-luciferase reporter assay determined the effect of IGF2BP2 on *TNF-α* mRNA stability in HEK-293T cells. Firefly luciferase activity was normalized to Renilla luciferase activity. (E) Schematic diagram showing the primer designs used for MeRIP. Data are presented as mean ± SD (n = 3; **p* < 0.05, ***p* < 0.01, ****p* < 0.001; ns, no significant; two-tailed unpaired *t*-test).

**Figure S8. The recombinant TNF-α restrains PDCoV replication.** (A and E) The cell viability of ST cells (A) or IPEC-J2 cells (E) treated with different concentrations of TNF-α. The values are shown as percentage of untreated cells. (B-D and F-H) PDCoV replication in TNF-α-treated ST cells (B-D) or IPEC-J2 cells (F-H). Cells were treated with increasing concentrations of TNF-α for 12 h, and then infected with PDCoV at an MOI of 0.1 for another 15 h. Cells were then subjected to absolute RT-qPCR of PDCoV *M* gene (B or F), plaque assays (C or G), and Western blot (D or H). The protein bands shown in panel D and H were quantified using ImageJ software. Data are presented as mean ± SD (n = 3; **p* < 0.05, ***p* < 0.01, ****p* < 0.001; ns, no significant; two-tailed unpaired *t*-test).
