# Supplementary material for: Deltacoronavirus Modulates circRNA cGLIS3 Metabolism to Evade Host Antiviral Response
Source: Adv Sci (Weinh). 2026 Jul 27:e76822. Online ahead of print. doi: 10.1002/advs.76822 (PMC13403727; doi:10.1002/advs.76822)
Supplement: Supplementary file 2 — Supporting File 2: advs76822‐sup‐0002‐FigureS1‐S8.pptx. [file ADVS-9999-e76822-s002.pptx]

## Slide 1
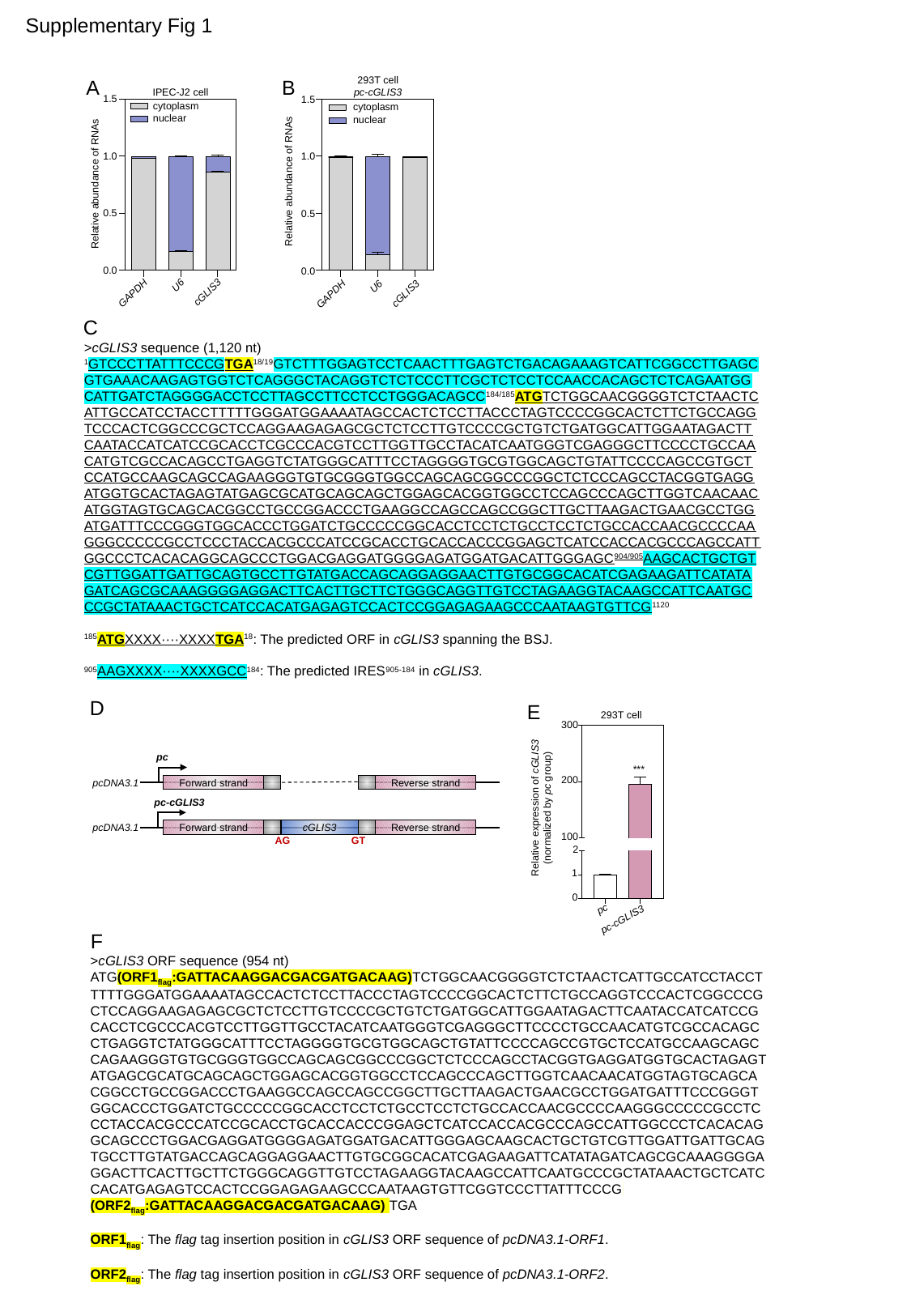

Supplementary Fig 1
293T cell
pc-cGLIS3
1.5
cytoplasm
nuclear
1.0
Relative abundance of RNAs
0.5
0.0
GAPDH
U6
cGLIS3
A
B
IPEC-J2 cell
1.5
cytoplasm
nuclear
1.0
Relative abundance of RNAs
0.5
0.0
GAPDH
U6
cGLIS3
C
>cGLIS3 sequence (1,120 nt)
1GTCCCTTATTTCCCGTGA18/19GTCTTTGGAGTCCTCAACTTTGAGTCTGACAGAAAGTCATTCGGCCTTGAGCGTGAAACAAGAGTGGTCTCAGGGCTACAGGTCTCTCCCTTCGCTCTCCTCCAACCACAGCTCTCAGAATGGCATTGATCTAGGGGACCTCCTTAGCCTTCCTCCTGGGACAGCC184/185ATGTCTGGCAACGGGGTCTCTAACTCATTGCCATCCTACCTTTTTGGGATGGAAAATAGCCACTCTCCTTACCCTAGTCCCCGGCACTCTTCTGCCAGGTCCCACTCGGCCCGCTCCAGGAAGAGAGCGCTCTCCTTGTCCCCGCTGTCTGATGGCATTGGAATAGACTTCAATACCATCATCCGCACCTCGCCCACGTCCTTGGTTGCCTACATCAATGGGTCGAGGGCTTCCCCTGCCAACATGTCGCCACAGCCTGAGGTCTATGGGCATTTCCTAGGGGTGCGTGGCAGCTGTATTCCCCAGCCGTGCTCCATGCCAAGCAGCCAGAAGGGTGTGCGGGTGGCCAGCAGCGGCCCGGCTCTCCCAGCCTACGGTGAGGATGGTGCACTAGAGTATGAGCGCATGCAGCAGCTGGAGCACGGTGGCCTCCAGCCCAGCTTGGTCAACAACATGGTAGTGCAGCACGGCCTGCCGGACCCTGAAGGCCAGCCAGCCGGCTTGCTTAAGACTGAACGCCTGGATGATTTCCCGGGTGGCACCCTGGATCTGCCCCCGGCACCTCCTCTGCCTCCTCTGCCACCAACGCCCCAAGGGCCCCCGCCTCCCTACCACGCCCATCCGCACCTGCACCACCCGGAGCTCATCCACCACGCCCAGCCATTGGCCCTCACACAGGCAGCCCTGGACGAGGATGGGGAGATGGATGACATTGGGAGC904/905AAGCACTGCTGTCGTTGGATTGATTGCAGTGCCTTGTATGACCAGCAGGAGGAACTTGTGCGGCACATCGAGAAGATTCATATAGATCAGCGCAAAGGGGAGGACTTCACTTGCTTCTGGGCAGGTTGTCCTAGAAGGTACAAGCCATTCAATGCCCGCTATAAACTGCTCATCCACATGAGAGTCCACTCCGGAGAGAAGCCCAATAAGTGTTCG1120
185ATGXXXX····XXXXTGA18: The predicted ORF in cGLIS3 spanning the BSJ.
905AAGXXXX····XXXXGCC184: The predicted IRES905-184 in cGLIS3.
D
E
293T cell
300
200
Relative expression of cGLIS3
(normalized by pc group)
100
0
pc
pc-cGLIS3
2
1
pc
pcDNA3.1
Forward strand
Reverse strand
pc-cGLIS3
pcDNA3.1
Forward strand
cGLIS3
Reverse strand
AG
GT
***
F
>cGLIS3 ORF sequence (954 nt)
ATG(ORF1flag:GATTACAAGGACGACGATGACAAG)TCTGGCAACGGGGTCTCTAACTCATTGCCATCCTACCTTTTTGGGATGGAAAATAGCCACTCTCCTTACCCTAGTCCCCGGCACTCTTCTGCCAGGTCCCACTCGGCCCGCTCCAGGAAGAGAGCGCTCTCCTTGTCCCCGCTGTCTGATGGCATTGGAATAGACTTCAATACCATCATCCGCACCTCGCCCACGTCCTTGGTTGCCTACATCAATGGGTCGAGGGCTTCCCCTGCCAACATGTCGCCACAGCCTGAGGTCTATGGGCATTTCCTAGGGGTGCGTGGCAGCTGTATTCCCCAGCCGTGCTCCATGCCAAGCAGCCAGAAGGGTGTGCGGGTGGCCAGCAGCGGCCCGGCTCTCCCAGCCTACGGTGAGGATGGTGCACTAGAGTATGAGCGCATGCAGCAGCTGGAGCACGGTGGCCTCCAGCCCAGCTTGGTCAACAACATGGTAGTGCAGCACGGCCTGCCGGACCCTGAAGGCCAGCCAGCCGGCTTGCTTAAGACTGAACGCCTGGATGATTTCCCGGGTGGCACCCTGGATCTGCCCCCGGCACCTCCTCTGCCTCCTCTGCCACCAACGCCCCAAGGGCCCCCGCCTCCCTACCACGCCCATCCGCACCTGCACCACCCGGAGCTCATCCACCACGCCCAGCCATTGGCCCTCACACAGGCAGCCCTGGACGAGGATGGGGAGATGGATGACATTGGGAGCAAGCACTGCTGTCGTTGGATTGATTGCAGTGCCTTGTATGACCAGCAGGAGGAACTTGTGCGGCACATCGAGAAGATTCATATAGATCAGCGCAAAGGGGAGGACTTCACTTGCTTCTGGGCAGGTTGTCCTAGAAGGTACAAGCCATTCAATGCCCGCTATAAACTGCTCATCCACATGAGAGTCCACTCCGGAGAGAAGCCCAATAAGTGTTCGGTCCCTTATTTCCCG (ORF2flag:GATTACAAGGACGACGATGACAAG) TGA
ORF1flag: The flag tag insertion position in cGLIS3 ORF sequence of pcDNA3.1-ORF1.
ORF2flag: The flag tag insertion position in cGLIS3 ORF sequence of pcDNA3.1-ORF2.

## Slide 2
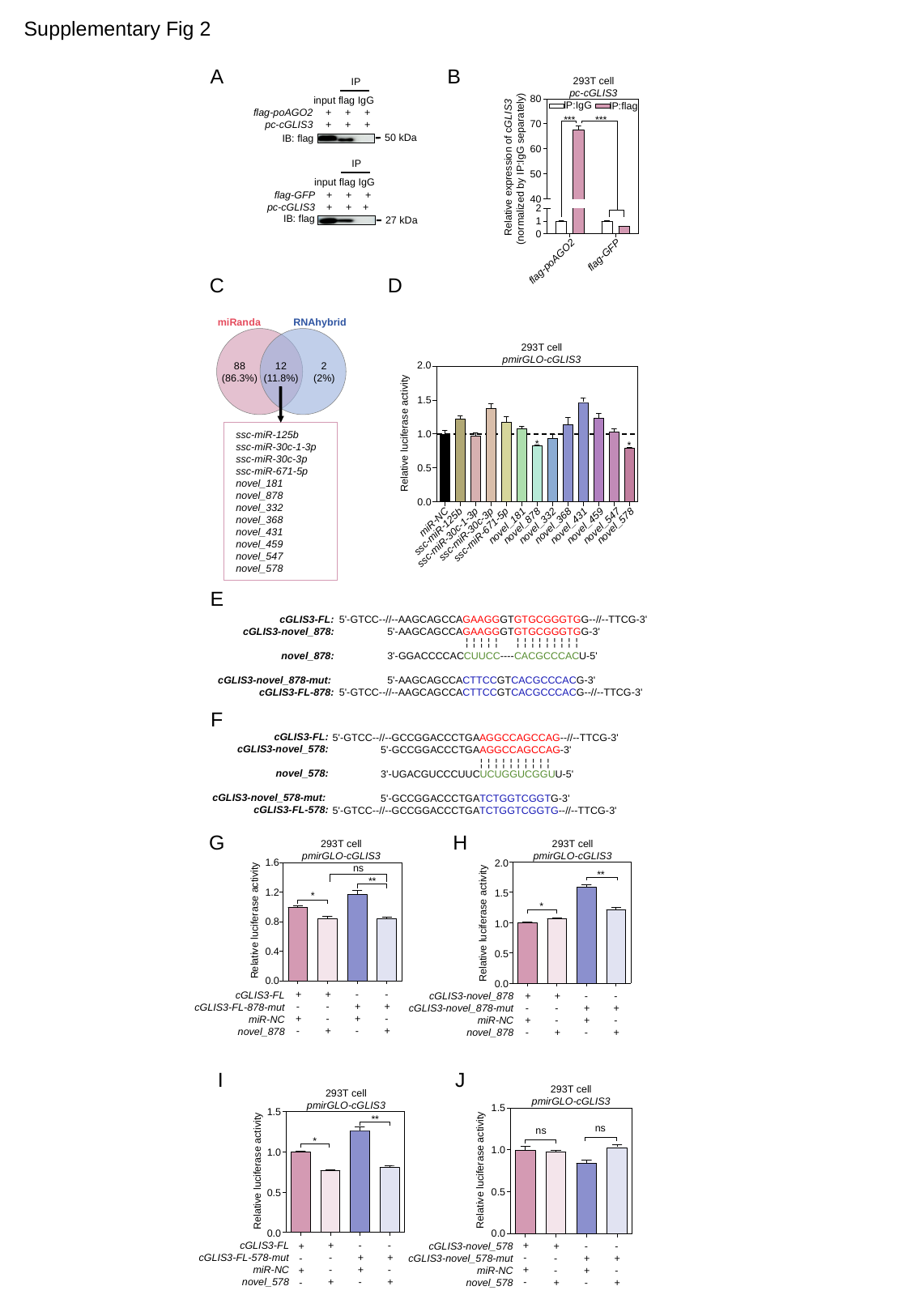

Supplementary Fig 2
A
IP
input flag IgG
+
+
+
+
+
+
flag-poAGO2
pc-cGLIS3
50 kDa
IB: flag
IP
input flag IgG
+
+
+
+
+
27 kDa
flag-GFP
pc-cGLIS3
IB: flag
+
B
293T cell
pc-cGLIS3
80
70
60
Relative expression of cGLIS3
(normalized by IP:IgG separately)
50
40
2
1
0
flag-poAGO2
flag-GFP
lP:IgG
lP:flag
***
***
C
D
miRanda
RNAhybrid
88
(86.3%)
12
(11.8%)
2
(2%)
ssc-miR-125b
ssc-miR-30c-1-3p
ssc-miR-30c-3p
ssc-miR-671-5p
novel_181
novel_878
novel_332
novel_368
novel_431
novel_459
novel_547
novel_578
293T cell
pmirGLO-cGLIS3
2.0
1.5
Relative luciferase activity
1.0
*
*
0.5
0.0
miR-NC
ssc-miR-125b
ssc-miR-30c-1-3p
ssc-miR-30c-3p
ssc-miR-671-5p
novel_181
novel_878
novel_332
novel_368
novel_431
novel_459
novel_547
novel_578
E
cGLIS3-FL:
cGLIS3-novel_878:
novel_878:
cGLIS3-novel_878-mut:
cGLIS3-FL-878:
5'-GTCC--//--AAGCAGCCAGAAGGGTGTGCGGGTGG--//--TTCG-3'
 5'-AAGCAGCCAGAAGGGTGTGCGGGTGG-3'
 3'-GGACCCCACCUUCC----CACGCCCACU-5'
 5'-AAGCAGCCACTTCCGTCACGCCCACG-3'
5'-GTCC--//--AAGCAGCCACTTCCGTCACGCCCACG--//--TTCG-3'
¦
¦
¦
¦
¦
¦
¦
¦
¦
¦
¦
¦
¦
¦
F
cGLIS3-FL:
cGLIS3-novel_578:
novel_578:
cGLIS3-novel_578-mut:
cGLIS3-FL-578:
5'-GTCC--//--GCCGGACCCTGAAGGCCAGCCAG--//--TTCG-3'
 5'-GCCGGACCCTGAAGGCCAGCCAG-3'
 3'-UGACGUCCCUUCUCUGGUCGGUU-5'
 5'-GCCGGACCCTGATCTGGTCGGTG-3'
5'-GTCC--//--GCCGGACCCTGATCTGGTCGGTG--//--TTCG-3'
¦
¦
¦
¦
¦
¦
¦
¦
¦
¦
G
H
293T cell
pmirGLO-cGLIS3
+
-
+
-
+
-
-
+
-
++
-
-+-+
cGLIS3-FL
cGLIS3-FL-878-mut
miR-NC
novel_878
1.6
ns
**
1.2
*
Relative luciferase activity
0.8
0.4
0.0
293T cell
pmirGLO-cGLIS3
2.0
**
1.5
*
1.0
Relative luciferase activity
0.5
0.0
+
-
+
-
+
-
-
+
-
++
-
-+-+
cGLIS3-novel_878
cGLIS3-novel_878-mut
miR-NC
novel_878
I
J
293T cell
pmirGLO-cGLIS3
1.5
ns
ns
1.0
Relative luciferase activity
0.5
0.0
+
-
+
-
+
-
-
+
-
++
-
-+-+
293T cell
pmirGLO-cGLIS3
1.5
**
*
1.0
Relative luciferase activity
0.5
0.0
cGLIS3-FL
cGLIS3-FL-578-mut
miR-NC
novel_578
+
-
-
+
-
++
-
-+-+
+
-
+
-
cGLIS3-novel_578
cGLIS3-novel_578-mut
miR-NC
novel_578

## Slide 3
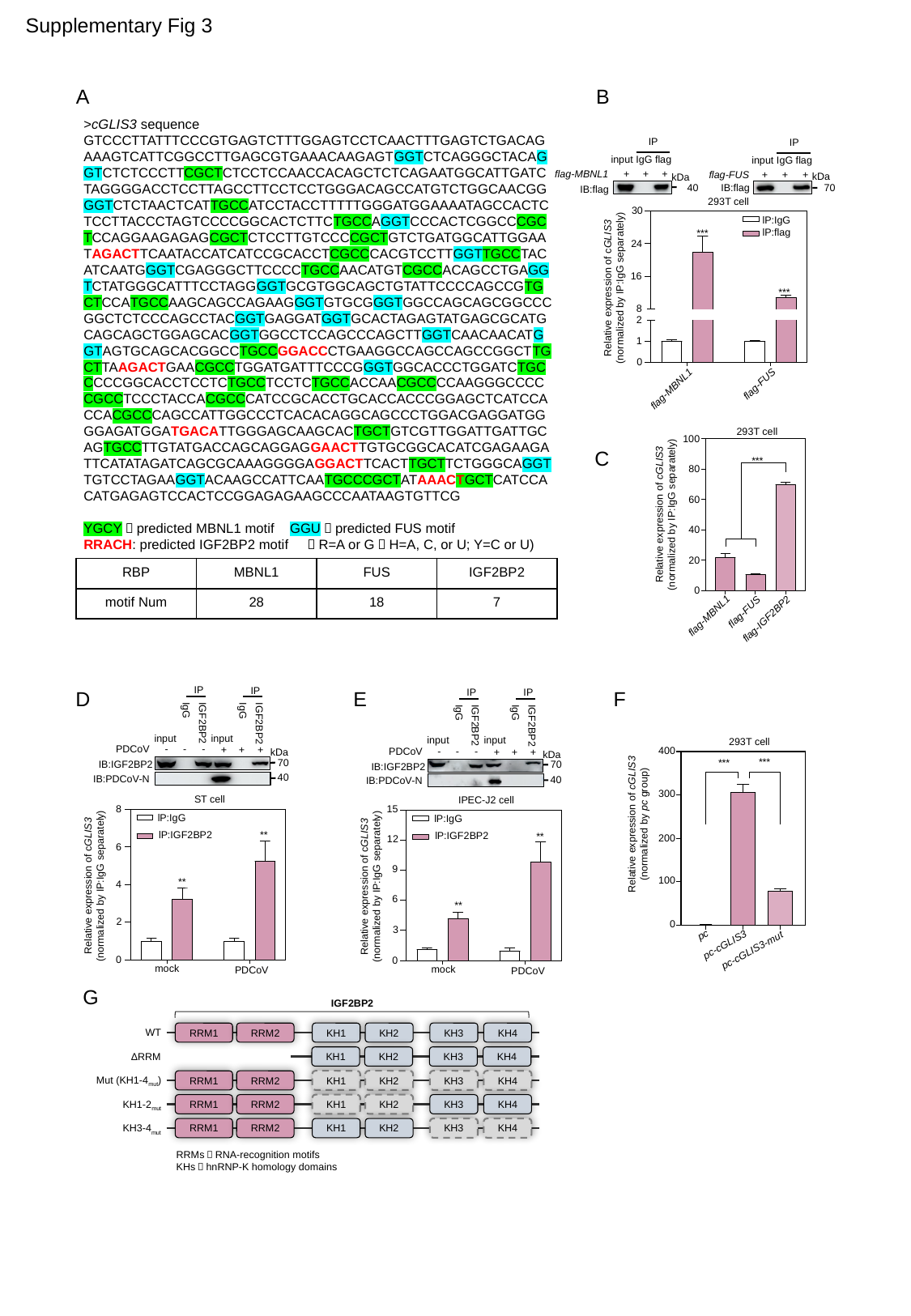

Supplementary Fig 3
A
B
>cGLIS3 sequence
GTCCCTTATTTCCCGTGAGTCTTTGGAGTCCTCAACTTTGAGTCTGACAGAAAGTCATTCGGCCTTGAGCGTGAAACAAGAGTGGTCTCAGGGCTACAGGTCTCTCCCTTCGCTCTCCTCCAACCACAGCTCTCAGAATGGCATTGATCTAGGGGACCTCCTTAGCCTTCCTCCTGGGACAGCCATGTCTGGCAACGGGGTCTCTAACTCATTGCCATCCTACCTTTTTGGGATGGAAAATAGCCACTCTCCTTACCCTAGTCCCCGGCACTCTTCTGCCAGGTCCCACTCGGCCCGCTCCAGGAAGAGAGCGCTCTCCTTGTCCCCGCTGTCTGATGGCATTGGAATAGACTTCAATACCATCATCCGCACCTCGCCCACGTCCTTGGTTGCCTACATCAATGGGTCGAGGGCTTCCCCTGCCAACATGTCGCCACAGCCTGAGGTCTATGGGCATTTCCTAGGGGTGCGTGGCAGCTGTATTCCCCAGCCGTGCTCCATGCCAAGCAGCCAGAAGGGTGTGCGGGTGGCCAGCAGCGGCCCGGCTCTCCCAGCCTACGGTGAGGATGGTGCACTAGAGTATGAGCGCATGCAGCAGCTGGAGCACGGTGGCCTCCAGCCCAGCTTGGTCAACAACATGGTAGTGCAGCACGGCCTGCCGGACCCTGAAGGCCAGCCAGCCGGCTTGCTTAAGACTGAACGCCTGGATGATTTCCCGGGTGGCACCCTGGATCTGCCCCCGGCACCTCCTCTGCCTCCTCTGCCACCAACGCCCCAAGGGCCCCCGCCTCCCTACCACGCCCATCCGCACCTGCACCACCCGGAGCTCATCCACCACGCCCAGCCATTGGCCCTCACACAGGCAGCCCTGGACGAGGATGGGGAGATGGATGACATTGGGAGCAAGCACTGCTGTCGTTGGATTGATTGCAGTGCCTTGTATGACCAGCAGGAGGAACTTGTGCGGCACATCGAGAAGATTCATATAGATCAGCGCAAAGGGGAGGACTTCACTTGCTTCTGGGCAGGTTGTCCTAGAAGGTACAAGCCATTCAATGCCCGCTATAAACTGCTCATCCACATGAGAGTCCACTCCGGAGAGAAGCCCAATAAGTGTTCG
YGCY：predicted MBNL1 motif GGU：predicted FUS motif
RRACH: predicted IGF2BP2 motif （R=A or G；H=A, C, or U; Y=C or U)
IP
input IgG flag
flag-MBNL1
+
+
+
kDa
40
IB:flag
IP
input IgG flag
flag-FUS
+
+
+
kDa
IB:flag
70
293T cell
lP:IgG
lP:flag
24
16
Relative expression of cGLIS3
(normalized by IP:IgG separately)
8
1
0
flag-MBNL1
flag-FUS
30
2
***
***
293T cell
100
80
60
Relative expression of cGLIS3
(normalized by IP:IgG separately)
40
20
0
flag-MBNL1
flag-IGF2BP2
flag-FUS
C
***
IP
IP
IgG
IgG
IGF2BP2
IGF2BP2
input
input
PDCoV
-
-
-
+
+
+
kDa
70
IB:IGF2BP2
40
IB:PDCoV-N
ST cell
8
lP:IgG
lP:IGF2BP2
6
Relative expression of cGLIS3
(normalized by IP:IgG separately)
4
2
0
PDCoV
mock
**
**
IP
IP
IgG
IgG
IGF2BP2
IGF2BP2
input
input
PDCoV
-
-
-
+
+
+
kDa
70
IB:IGF2BP2
40
IB:PDCoV-N
IPEC-J2 cell
15
lP:IgG
lP:IGF2BP2
 9
Relative expression of cGLIS3
(normalized by IP:IgG separately)
6
3
0
PDCoV
mock
**
12
**
D
E
F
293T cell
400
***
***
300
Relative expression of cGLIS3
(normalized by pc group)
200
100
0
pc
pc-cGLIS3
pc-cGLIS3-mut
G
IGF2BP2
WT
RRM1
RRM2
KH1
KH2
KH3
KH4
ΔRRM
KH1
KH2
KH3
KH4
Mut (KH1-4mut)
RRM1
RRM2
KH1
KH2
KH3
KH4
KH1-2mut
RRM1
RRM2
KH1
KH2
KH3
KH4
KH3-4mut
RRM1
RRM2
KH1
KH2
KH3
KH4
RRMs：RNA-recognition motifs
KHs：hnRNP-K homology domains
| RBP | MBNL1 | FUS | IGF2BP2 |
| --- | --- | --- | --- |
| motif Num | 28 | 18 | 7 |

## Slide 4
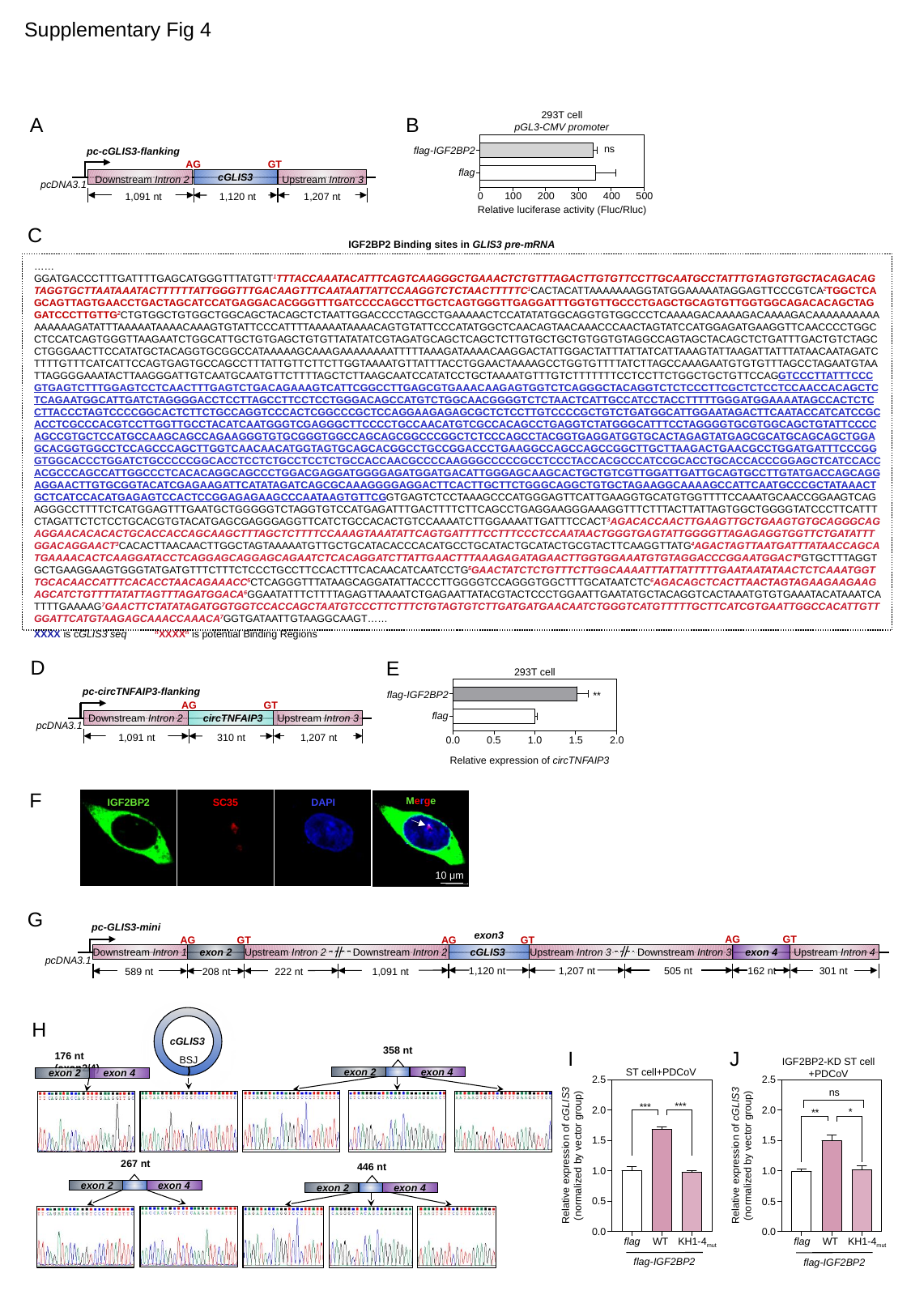

Supplementary Fig 4
293T cell
pGL3-CMV promoter
B
ns
flag-IGF2BP2
flag
0
100
200
300
400
500
Relative luciferase activity (Fluc/Rluc)
A
pc-cGLIS3-flanking
AG
 GT
cGLIS3
pcDNA3.1
Downstream Intron 2
Upstream Intron 3
1,091 nt
1,120 nt
1,207 nt
C
IGF2BP2 Binding sites in GLIS3 pre-mRNA
……GGATGACCCTTTGATTTTGAGCATGGGTTTATGTT1TTTACCAAATACATTTCAGTCAAGGGCTGAAACTCTGTTTAGACTTGTGTTCCTTGCAATGCCTATTTGTAGTGTGCTACAGACAGTAGGTGCTTAATAAATACTTTTTTATTGGGTTTGACAAGTTTCAATAATTATTCCAAGGTCTCTAACTTTTTC1CACTACATTAAAAAAAGGTATGGAAAAATAGGAGTTCCCGTCA2TGGCTCAGCAGTTAGTGAACCTGACTAGCATCCATGAGGACACGGGTTTGATCCCCAGCCTTGCTCAGTGGGTTGAGGATTTGGTGTTGCCCTGAGCTGCAGTGTTGGTGGCAGACACAGCTAGGATCCCTTGTTG2CTGTGGCTGTGGCTGGCAGCTACAGCTCTAATTGGACCCCTAGCCTGAAAAACTCCATATATGGCAGGTGTGGCCCTCAAAAGACAAAAGACAAAAGACAAAAAAAAAAAAAAAAGATATTTAAAAATAAAACAAAGTGTATTCCCATTTTAAAAATAAAACAGTGTATTCCCATATGGCTCAACAGTAACAAACCCAACTAGTATCCATGGAGATGAAGGTTCAACCCCTGGCCTCCATCAGTGGGTTAAGAATCTGGCATTGCTGTGAGCTGTGTTATATATCGTAGATGCAGCTCAGCTCTTGTGCTGCTGTGGTGTAGGCCAGTAGCTACAGCTCTGATTTGACTGTCTAGCCTGGGAACTTCCATATGCTACAGGTGCGGCCATAAAAAGCAAAGAAAAAAAATTTTTAAAGATAAAACAAGGACTATTGGACTATTTATTATCATTAAAGTATTAAGATTATTTATAACAATAGATCTTTTGTTTCATCATTCCAGTGAGTGCCAGCCTTTATTGTTCTTCTTGGTAAAATGTTATTTACCTGGAACTAAAAGCCTGGTGTTTTATCTTAGCCAAAGAATGTGTGTTTAGCCTAGAATGTAATTAGGGGAAATACTTAAGGGATTGTCAATGCAATGTTCTTTTAGCTCTTAAGCAATCCATATCCTGCTAAAATGTTTGTCTTTTTTTCCTCCTTCTGGCTGCTGTTCCAGGTCCCTTATTTCCCGTGAGTCTTTGGAGTCCTCAACTTTGAGTCTGACAGAAAGTCATTCGGCCTTGAGCGTGAAACAAGAGTGGTCTCAGGGCTACAGGTCTCTCCCTTCGCTCTCCTCCAACCACAGCTCTCAGAATGGCATTGATCTAGGGGACCTCCTTAGCCTTCCTCCTGGGACAGCCATGTCTGGCAACGGGGTCTCTAACTCATTGCCATCCTACCTTTTTGGGATGGAAAATAGCCACTCTCCTTACCCTAGTCCCCGGCACTCTTCTGCCAGGTCCCACTCGGCCCGCTCCAGGAAGAGAGCGCTCTCCTTGTCCCCGCTGTCTGATGGCATTGGAATAGACTTCAATACCATCATCCGCACCTCGCCCACGTCCTTGGTTGCCTACATCAATGGGTCGAGGGCTTCCCCTGCCAACATGTCGCCACAGCCTGAGGTCTATGGGCATTTCCTAGGGGTGCGTGGCAGCTGTATTCCCCAGCCGTGCTCCATGCCAAGCAGCCAGAAGGGTGTGCGGGTGGCCAGCAGCGGCCCGGCTCTCCCAGCCTACGGTGAGGATGGTGCACTAGAGTATGAGCGCATGCAGCAGCTGGAGCACGGTGGCCTCCAGCCCAGCTTGGTCAACAACATGGTAGTGCAGCACGGCCTGCCGGACCCTGAAGGCCAGCCAGCCGGCTTGCTTAAGACTGAACGCCTGGATGATTTCCCGGGTGGCACCCTGGATCTGCCCCCGGCACCTCCTCTGCCTCCTCTGCCACCAACGCCCCAAGGGCCCCCGCCTCCCTACCACGCCCATCCGCACCTGCACCACCCGGAGCTCATCCACCACGCCCAGCCATTGGCCCTCACACAGGCAGCCCTGGACGAGGATGGGGAGATGGATGACATTGGGAGCAAGCACTGCTGTCGTTGGATTGATTGCAGTGCCTTGTATGACCAGCAGGAGGAACTTGTGCGGTACATCGAGAAGATTCATATAGATCAGCGCAAAGGGGAGGACTTCACTTGCTTCTGGGCAGGCTGTGCTAGAAGGCAAAAGCCATTCAATGCCCGCTATAAACTGCTCATCCACATGAGAGTCCACTCCGGAGAGAAGCCCAATAAGTGTTCGGTGAGTCTCCTAAAGCCCATGGGAGTTCATTGAAGGTGCATGTGGTTTTCCAAATGCAACCGGAAGTCAGAGGGCCTTTTCTCATGGAGTTTGAATGCTGGGGGTCTAGGTGTCCATGAGATTTGACTTTTCTTCAGCCTGAGGAAGGGAAAGGTTTCTTTACTTATTAGTGGCTGGGGTATCCCTTCATTTCTAGATTCTCTCCTGCACGTGTACATGAGCGAGGGAGGTTCATCTGCCACACTGTCCAAAATCTTGGAAAATTGATTTCCACT3AGACACCAACTTGAAGTTGCTGAAGTGTGCAGGGCAGAGGAACACACACTGCACCACCAGCAAGCTTTAGCTCTTTTCCAAAGTAAATATTCAGTGATTTTCCTTTCCCTCCAATAACTGGGTGAGTATTGGGGTTAGAGAGGTGGTTCTGATATTTGGACAGGAACT3CACACTTAACAACTTGGCTAGTAAAAATGTTGCTGCATACACCCACATGCCTGCATACTGCATACTGCGTACTTCAAGGTTATG4AGACTAGTTAATGATTTATAACCAGCATGAAAACACTCAAGGATACCTCAGGAGCAGGAGCAGAATCTCACAGGATCTTATTGAACTTTAAAGAGATAGAACTTGGTGGAAATGTGTAGGACCCGGAATGGACT4GTGCTTTAGGTGCTGAAGGAAGTGGGTATGATGTTTCTTTCTCCCTGCCTTCCACTTTCACAACATCAATCCTG5GAACTATCTCTGTTTCTTGGCAAAATTTATTATTTTTGAATAATATAACTCTCAAATGGTTGCACAACCATTTCACACCTAACAGAAACC5CTCAGGGTTTATAAGCAGGATATTACCCTTGGGGTCCAGGGTGGCTTTGCATAATCTC6AGACAGCTCACTTAACTAGTAGAAGAAGAAGAGCATCTGTTTTATATTAGTTTAGATGGACA6GGAATATTTCTTTTAGAGTTAAAATCTGAGAATTATACGTACTCCCTGGAATTGAATATGCTACAGGTCACTAAATGTGTGAAATACATAAATCATTTTGAAAAG7GAACTTCTATATAGATGGTGGTCCACCAGCTAATGTCCCTTCTTTCTGTAGTGTCTTGATGATGAACAATCTGGGTCATGTTTTTGCTTCATCGTGAATTGGCCACATTGTTGGATTCATGTAAGAGCAAACCAAACA7GGTGATAATTGTAAGGCAAGT……
XXXX is cGLIS3 seq nXXXXn is potential Binding Regions
D
E
293T cell
flag-IGF2BP2
**
flag
0.0
0.5
1.0
1.5
2.0
pc-circTNFAIP3-flanking
AG
 GT
circTNFAIP3
pcDNA3.1
Upstream Intron 3
Downstream Intron 2
1,091 nt
310 nt
1,207 nt
Relative expression of circTNFAIP3
F
Merge
IGF2BP2
SC35
DAPI
10 μm
G
pc-GLIS3-mini
AG
 GT
 GT
AG
AG
 GT
Upstream Intron 2
Downstream Intron 1
Downstream Intron 2
Upstream Intron 3
Downstream Intron 3
Upstream Intron 4
exon 2
cGLIS3
exon 4
pcDNA3.1
1,120 nt
1,207 nt
505 nt
162 nt
301 nt
589 nt
208 nt
222 nt
1,091 nt
exon3
cGLIS3
BSJ
176 nt (exon2/4)
exon 2
exon 4
358 nt
exon 2
exon 4
267 nt
exon 2
exon 4
446 nt
exon 2
exon 4
H
I
J
IGF2BP2-KD ST cell
+PDCoV
2.5
2.0
*
**
1.5
Relative expression of cGLIS3
(normalized by vector group)
1.0
0.5
0.0
flag
WT
KH1-4mut
flag-IGF2BP2
ns
ST cell+PDCoV
2.5
***
***
2.0
1.5
Relative expression of cGLIS3
(normalized by vector group)
1.0
0.5
0.0
flag
WT
KH1-4mut
flag-IGF2BP2

## Slide 5
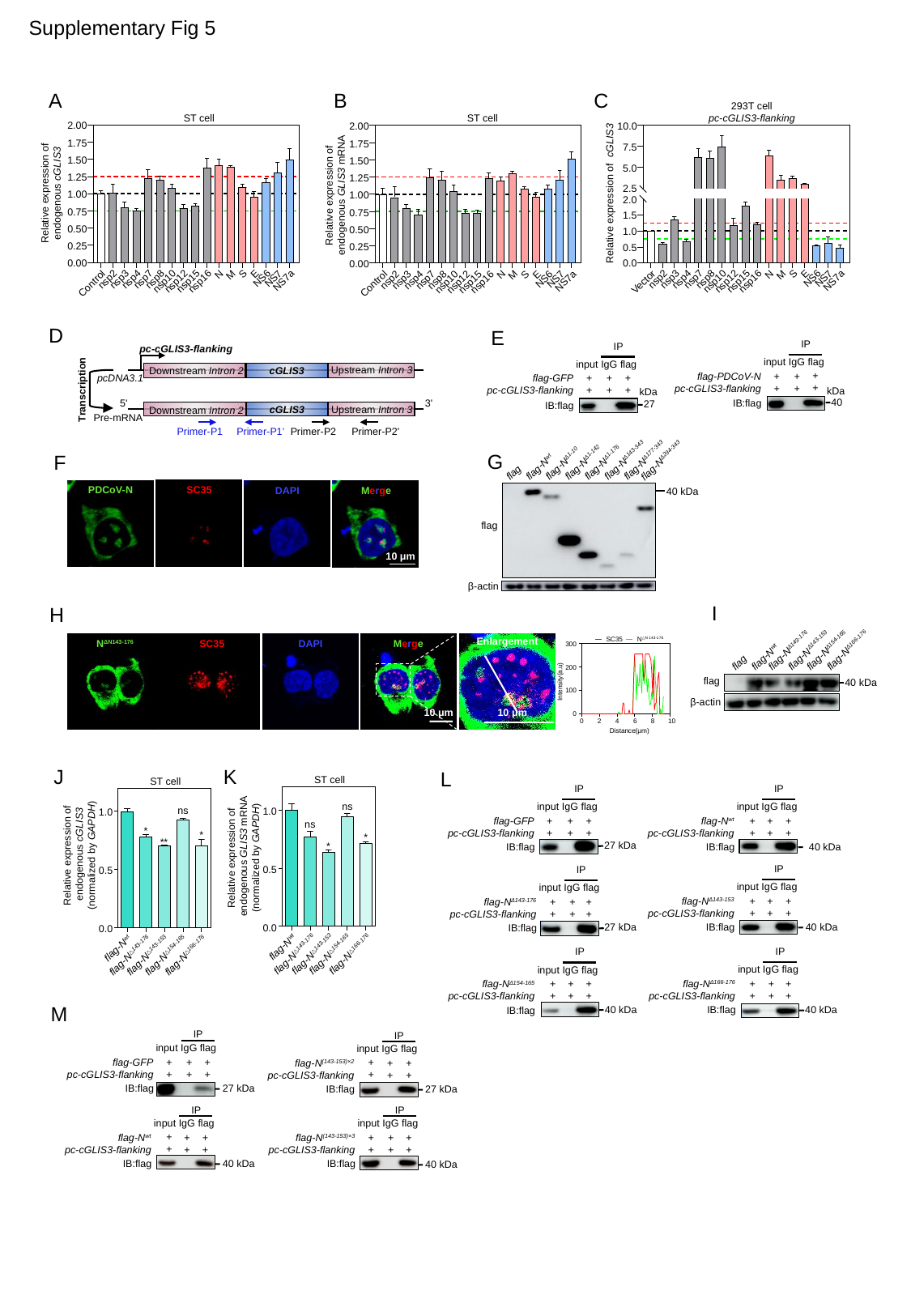

Supplementary Fig 5
A
B
C
293T cell
pc-cGLIS3-flanking
Relative expression of cGLIS3
Vector
nsp2
nsp3
nsp4
nsp7
nsp8
nsp10
nsp12
nsp15
nsp16
N
M
S
E
NS6
NS7
NS7a
10.0
7.5
5.0
2.5
2.0
1.5
1.0
0.5
0.0
ST cell
2.00
1.75
1.50
1.25
Relative expression of endogenous GLIS3 mRNA
1.00
0.75
0.50
0.25
0.00
Control
nsp2
nsp3
nsp4
nsp7
nsp8
nsp10
nsp12
nsp15
nsp16
N
M
S
E
NS6
NS7
NS7a
ST cell
2.00
1.75
1.50
1.25
Relative expression of endogenous cGLIS3
1.00
0.75
0.50
0.25
0.00
Control
nsp2
nsp3
nsp4
nsp7
nsp8
nsp10
nsp12
nsp15
nsp16
N
M
S
E
NS6
NS7
NS7a
D
E
IP
input IgG flag
flag-GFP
pc-cGLIS3-flanking
+
+
kDa
27
IB:flag
IP
input IgG flag
flag-PDCoV-N
pc-cGLIS3-flanking
+
+
kDa
40
IB:flag
+
+
+
+
+
+
+
+
pc-cGLIS3-flanking
Upstream Intron 3
Downstream Intron 2
cGLIS3
pcDNA3.1
Transcription
5'
3'
Upstream Intron 3
Downstream Intron 2
cGLIS3
Pre-mRNA
Primer-P1
Primer-P1'
Primer-P2
Primer-P2'
G
F
flag-NΔ1-176
flag-NΔ143-343
flag-NΔ177-343
flag-NΔ1-142
flag-NΔ1-10
flag-NΔ294-343
flag
flag-Nwt
40 kDa
flag
β-actin
SC35
PDCoV-N
DAPI
Merge
10 μm
I
H
Enlargement
10 μm
NΔN143-176
SC35
DAPI
Merge
10 μm
SC35
N△N 143-176
300
200
Intensity(a.u)
100
0
0
2
4
6
8
10
Distance(μm)
flag-NΔ143-153
flag-NΔ166-176
flag-NΔ154-165
flag-NΔ143-176
flag
flag-Nwt
flag
40 kDa
β-actin
J
ST cell
ns
1.0
*
*
**
Relative expression of
 endogenous cGLIS3
(normalized by GAPDH)
0.5
0.0
flag-Nwt
flag-N△143-176
flag-N△143-153
flag-N△154-165
flag-N△166-176
K
L
IP
input IgG flag
flag-GFP
pc-cGLIS3-flanking
+
+
27 kDa
IB:flag
IP
IP
input IgG flag
+
+
IB:flag
40 kDa
flag-Nwt
pc-cGLIS3-flanking
IP
input IgG flag
flag-NΔ143-153
pc-cGLIS3-flanking
+
+
IB:flag
40 kDa
input IgG flag
flag-NΔ143-176
pc-cGLIS3-flanking
+
+
27 kDa
IB:flag
IP
input IgG flag
flag-NΔ166-176
pc-cGLIS3-flanking
+
+
IB:flag
40 kDa
IP
input IgG flag
flag-NΔ154-165
pc-cGLIS3-flanking
+
+
40 kDa
IB:flag
ST cell
ns
1.0
ns
*
*
Relative expression of
 endogenous GLIS3 mRNA
(normalized by GAPDH)
0.5
0.0
flag-Nwt
flag-N△143-176
flag-N△143-153
flag-N△154-165
flag-N△166-176
M
IP
input IgG flag
flag-GFP
pc-cGLIS3-flanking
+
+
IB:flag
27 kDa
IP
input IgG flag
flag-N(143-153)×2
pc-cGLIS3-flanking
+
+
IB:flag
27 kDa
IP
IP
input IgG flag
flag-N(143-153)×3
pc-cGLIS3-flanking
+
+
IB:flag
40 kDa
input IgG flag
flag-Nwt
pc-cGLIS3-flanking
+
+
IB:flag
40 kDa
+
+
+
+
+
+
+
+
+
+
+
+
+
+
+
+
+
+
+
+
+
+
+
+
+
+
+
+
+
+
+
+
+
+
+
+
+
+
+
+

## Slide 6
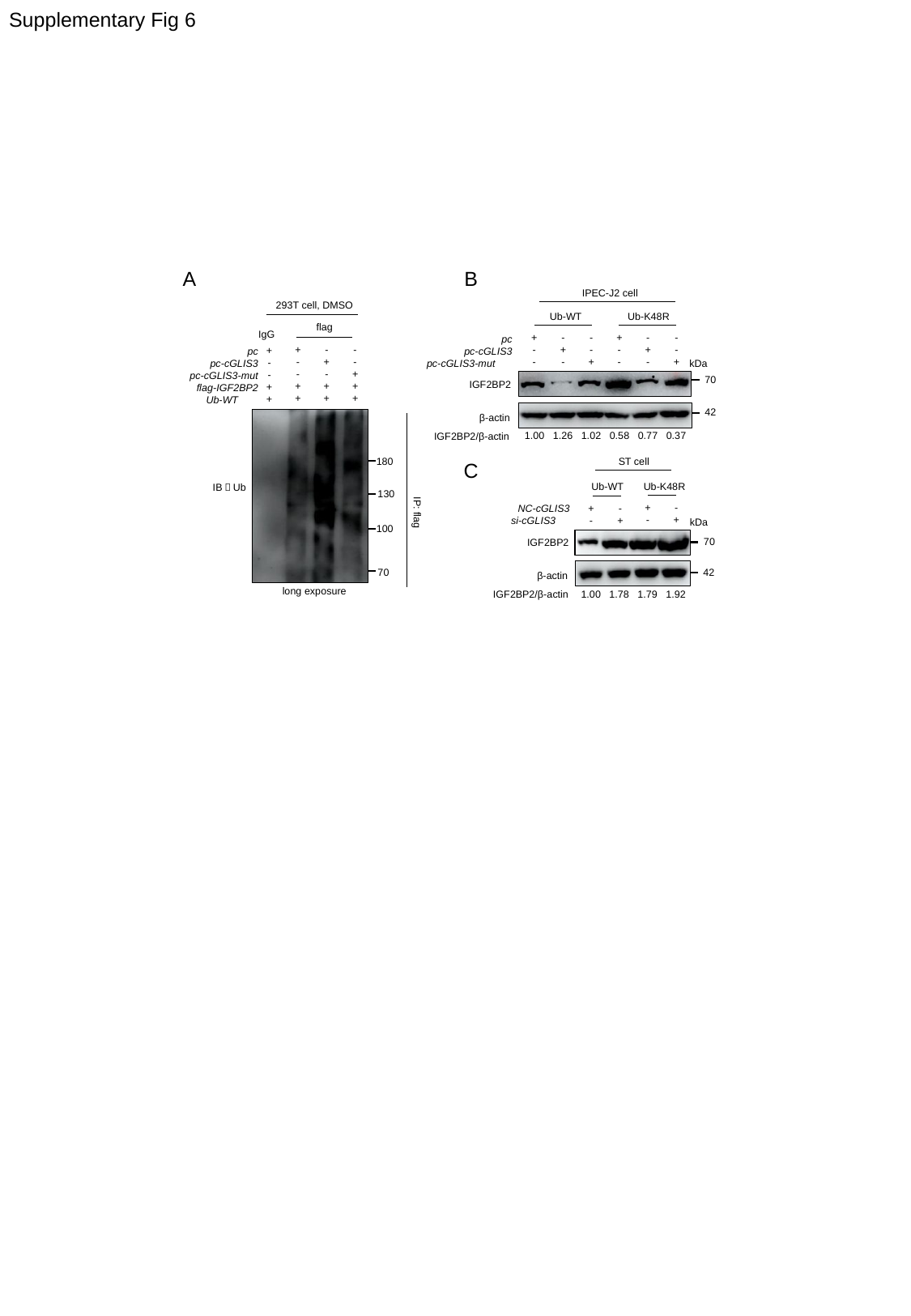

Supplementary Fig 6
A
B
IPEC-J2 cell
Ub-K48R
+
-
-
-
+
-
-
-
+
Ub-WT
+
-
-
-
+
-
-
-
+
kDa
70
IGF2BP2
42
β-actin
pc
pc-cGLIS3
pc-cGLIS3-mut
IGF2BP2/β-actin
| 1.00 1.26 1.02 0.58 0.77 0.37 |
| --- |
293T cell, DMSO
flag
IgG
+
-
-
+
+
-
+
-
+
+
-
-
+
+
+
+
-
-
+
+
pc
pc-cGLIS3
pc-cGLIS3-mut
flag-IGF2BP2
Ub-WT
IP: flag
180
130
100
70
IB：Ub
long exposure
ST cell
Ub-K48R
+
-
-
+
Ub-WT
+
-
-
+
kDa
IGF2BP2
β-actin
NC-cGLIS3
si-cGLIS3
70
42
IGF2BP2/β-actin
| 1.00 1.78 1.79 1.92 |
| --- |
C

## Slide 7
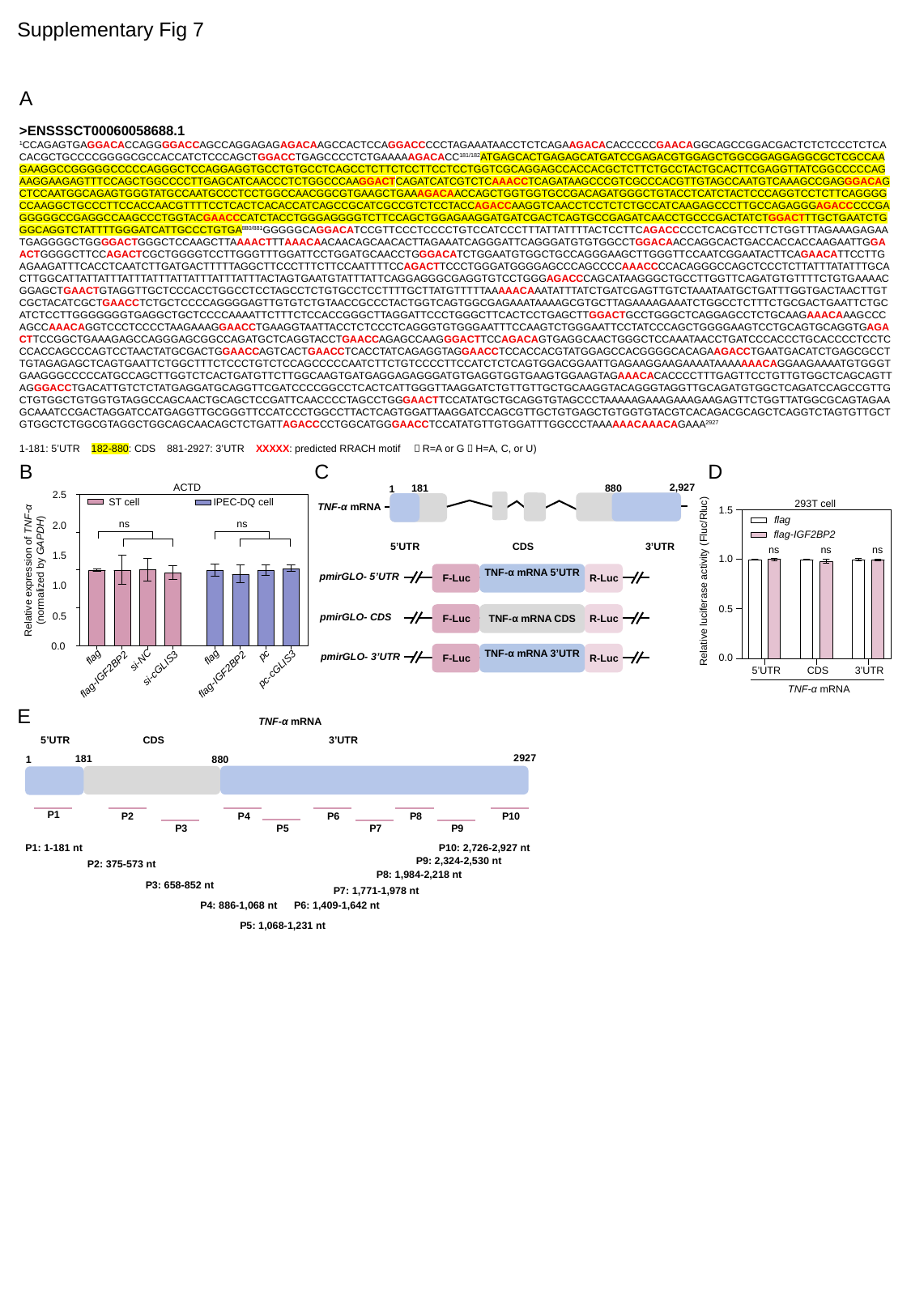

Supplementary Fig 7
A
>ENSSSCT00060058688.1
1CCAGAGTGAGGACACCAGGGGACCAGCCAGGAGAGAGACAAGCCACTCCAGGACCCCCTAGAAATAACCTCTCAGAAGACACACCCCCGAACAGGCAGCCGGACGACTCTCTCCCTCTCACACGCTGCCCCGGGGCGCCACCATCTCCCAGCTGGACCTGAGCCCCTCTGAAAAAGACACC181/182ATGAGCACTGAGAGCATGATCCGAGACGTGGAGCTGGCGGAGGAGGCGCTCGCCAAGAAGGCCGGGGGCCCCCAGGGCTCCAGGAGGTGCCTGTGCCTCAGCCTCTTCTCCTTCCTCCTGGTCGCAGGAGCCACCACGCTCTTCTGCCTACTGCACTTCGAGGTTATCGGCCCCCAGAAGGAAGAGTTTCCAGCTGGCCCCTTGAGCATCAACCCTCTGGCCCAAGGACTCAGATCATCGTCTCAAACCTCAGATAAGCCCGTCGCCCACGTTGTAGCCAATGTCAAAGCCGAGGGACAGCTCCAATGGCAGAGTGGGTATGCCAATGCCCTCCTGGCCAACGGCGTGAAGCTGAAAGACAACCAGCTGGTGGTGCCGACAGATGGGCTGTACCTCATCTACTCCCAGGTCCTCTTCAGGGGCCAAGGCTGCCCTTCCACCAACGTTTTCCTCACTCACACCATCAGCCGCATCGCCGTCTCCTACCAGACCAAGGTCAACCTCCTCTCTGCCATCAAGAGCCCTTGCCAGAGGGAGACCCCCGAGGGGGCCGAGGCCAAGCCCTGGTACGAACCCATCTACCTGGGAGGGGTCTTCCAGCTGGAGAAGGATGATCGACTCAGTGCCGAGATCAACCTGCCCGACTATCTGGACTTTGCTGAATCTGGGCAGGTCTATTTTGGGATCATTGCCCTGTGA880/881GGGGGCAGGACATCCGTTCCCTCCCCTGTCCATCCCTTTATTATTTTACTCCTTCAGACCCCCTCACGTCCTTCTGGTTTAGAAAGAGAATGAGGGGCTGGGGACTGGGCTCCAAGCTTAAAACTTTAAACAACAACAGCAACACTTAGAAATCAGGGATTCAGGGATGTGTGGCCTGGACAACCAGGCACTGACCACCACCAAGAATTGGAACTGGGGCTTCCAGACTCGCTGGGGTCCTTGGGTTTGGATTCCTGGATGCAACCTGGGACATCTGGAATGTGGCTGCCAGGGAAGCTTGGGTTCCAATCGGAATACTTCAGAACATTCCTTGAGAAGATTTCACCTCAATCTTGATGACTTTTTAGGCTTCCCTTTCTTCCAATTTTCCAGACTTCCCTGGGATGGGGAGCCCAGCCCCAAACCCCACAGGGCCAGCTCCCTCTTATTTATATTTGCACTTGGCATTATTATTTATTTATTTATTATTTATTTATTTACTAGTGAATGTATTTATTCAGGAGGGCGAGGTGTCCTGGGAGACCCAGCATAAGGGCTGCCTTGGTTCAGATGTGTTTTCTGTGAAAACGGAGCTGAACTGTAGGTTGCTCCCACCTGGCCTCCTAGCCTCTGTGCCTCCTTTTGCTTATGTTTTTAAAAACAAATATTTATCTGATCGAGTTGTCTAAATAATGCTGATTTGGTGACTAACTTGTCGCTACATCGCTGAACCTCTGCTCCCCAGGGGAGTTGTGTCTGTAACCGCCCTACTGGTCAGTGGCGAGAAATAAAAGCGTGCTTAGAAAAGAAATCTGGCCTCTTTCTGCGACTGAATTCTGCATCTCCTTGGGGGGGTGAGGCTGCTCCCCAAAATTCTTTCTCCACCGGGCTTAGGATTCCCTGGGCTTCACTCCTGAGCTTGGACTGCCTGGGCTCAGGAGCCTCTGCAAGAAACAAAGCCCAGCCAAACAGGTCCCTCCCCTAAGAAAGGAACCTGAAGGTAATTACCTCTCCCTCAGGGTGTGGGAATTTCCAAGTCTGGGAATTCCTATCCCAGCTGGGGAAGTCCTGCAGTGCAGGTGAGACTTCCGGCTGAAAGAGCCAGGGAGCGGCCAGATGCTCAGGTACCTGAACCAGAGCCAAGGGACTTCCAGACAGTGAGGCAACTGGGCTCCAAATAACCTGATCCCACCCTGCACCCCTCCTCCCACCAGCCCAGTCCTAACTATGCGACTGGAACCAGTCACTGAACCTCACCTATCAGAGGTAGGAACCTCCACCACGTATGGAGCCACGGGGCACAGAAGACCTGAATGACATCTGAGCGCCTTGTAGAGAGCTCAGTGAATTCTGGCTTTCTCCCTGTCTCCAGCCCCCAATCTTCTGTCCCCTTCCATCTCTCAGTGGACGGAATTGAGAAGGAAGAAAATAAAAAAACAGGAAGAAAATGTGGGTGAAGGGCCCCCATGCCAGCTTGGTCTCACTGATGTTCTTGGCAAGTGATGAGGAGAGGGATGTGAGGTGGTGAAGTGGAAGTAGAAACACACCCCTTTGAGTTCCTGTTGTGGCTCAGCAGTTAGGGACCTGACATTGTCTCTATGAGGATGCAGGTTCGATCCCCGGCCTCACTCATTGGGTTAAGGATCTGTTGTTGCTGCAAGGTACAGGGTAGGTTGCAGATGTGGCTCAGATCCAGCCGTTGCTGTGGCTGTGGTGTAGGCCAGCAACTGCAGCTCCGATTCAACCCCTAGCCTGGGAACTTCCATATGCTGCAGGTGTAGCCCTAAAAAGAAAGAAAGAAGAGTTCTGGTTATGGCGCAGTAGAAGCAAATCCGACTAGGATCCATGAGGTTGCGGGTTCCATCCCTGGCCTTACTCAGTGGATTAAGGATCCAGCGTTGCTGTGAGCTGTGGTGTACGTCACAGACGCAGCTCAGGTCTAGTGTTGCTGTGGCTCTGGCGTAGGCTGGCAGCAACAGCTCTGATTAGACCCCTGGCATGGGAACCTCCATATGTTGTGGATTTGGCCCTAAAAAACAAACAGAAA2927
1-181: 5’UTR 182-880: CDS 881-2927: 3’UTR XXXXX: predicted RRACH motif （R=A or G；H=A, C, or U)
B
C
D
1.5
flag
flag-IGF2BP2
ns
ns
ns
1.0
Relative luciferase activity (Fluc/Rluc)
0.5
0.0
5’UTR
CDS
3’UTR
TNF-α mRNA
293T cell
2.5
ST cell
IPEC-DQ cell
2.0
1.5
Relative expression of TNF-α
(normalized by GAPDH)
1.0
0.5
0.0
flag
si-NC
si-cGLIS3
flag-IGF2BP2
ns
ns
ACTD
flag
pc
pc-cGLIS3
flag-IGF2BP2
2,927
880
181
1
TNF-α mRNA
5’UTR
CDS
3’UTR
TNF-α mRNA 5’UTR
F-Luc
R-Luc
pmirGLO- 5’UTR
F-Luc
TNF-α mRNA CDS
R-Luc
pmirGLO- CDS
TNF-α mRNA 3’UTR
F-Luc
R-Luc
pmirGLO- 3’UTR
E
TNF-α mRNA
5’UTR
CDS
3’UTR
2927
181
1
880
P1
P10
P2
P4
P6
P8
P3
P5
P7
P9
P1: 1-181 nt
P10: 2,726-2,927 nt
P9: 2,324-2,530 nt
P2: 375-573 nt
P8: 1,984-2,218 nt
P3: 658-852 nt
P7: 1,771-1,978 nt
P4: 886-1,068 nt
P6: 1,409-1,642 nt
P5: 1,068-1,231 nt

## Slide 8
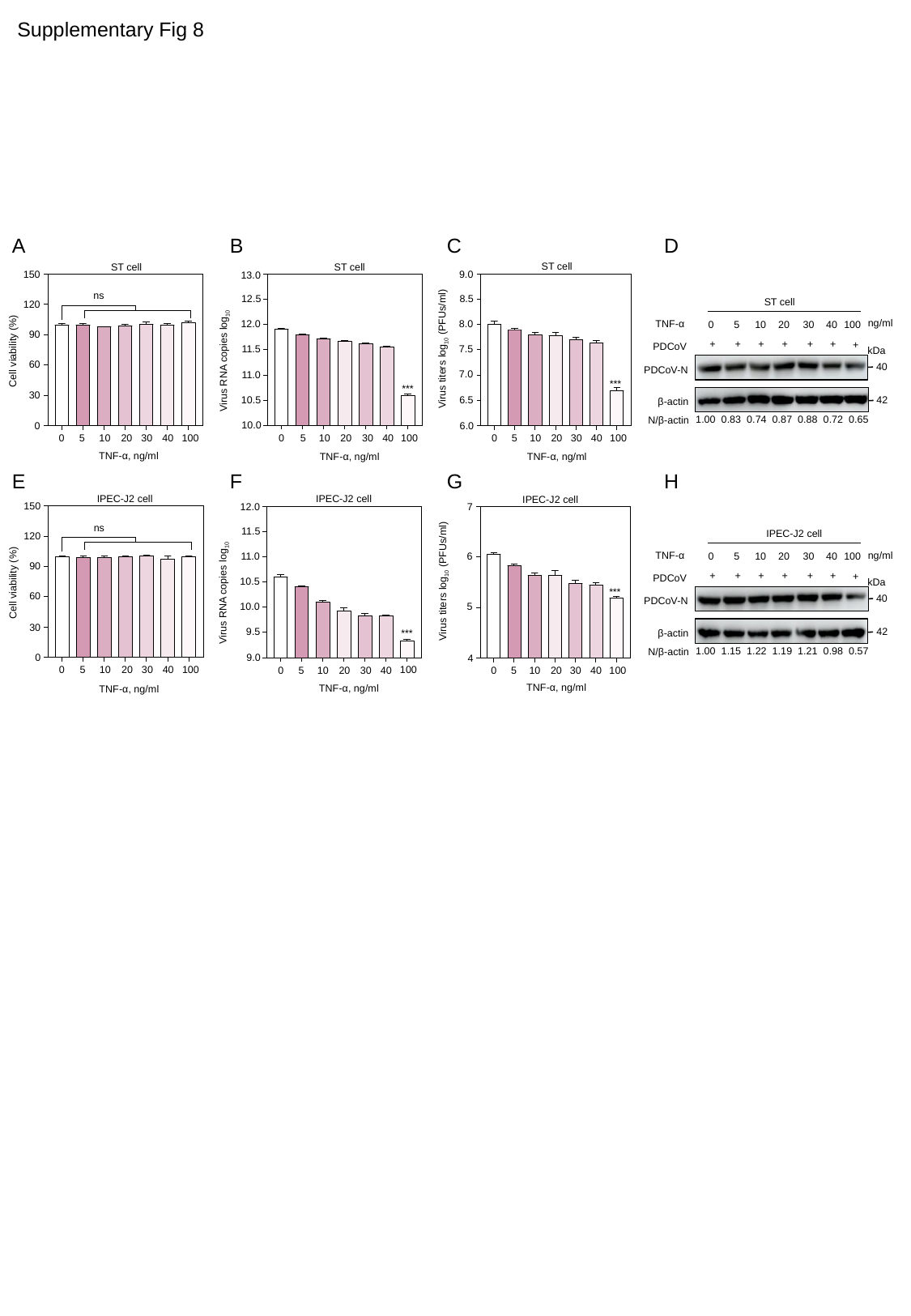

Supplementary Fig 8
A
B
C
D
ST cell
8.5
8.0
7.5
7.0
6.5
6.0
0
5
10
20
30
40
100
TNF-α, ng/ml
9.0
***
Virus titers log10 (PFUs/ml)
ST cell
150
120
90
Cell viability (%)
60
30
0
0
5
10
20
30
40
100
ns
TNF-α, ng/ml
ST cell
12.5
12.0
11.5
11.0
10.5
10.0
0
30
5
10
20
40
100
Virus RNA copies log10
TNF-α, ng/ml
13.0
***
ST cell
ng/ml
TNF-α
30
0
5
10
20
40
100
+
+
+
+
+
+
+
PDCoV
kDa
40
PDCoV-N
42
β-actin
N/β-actin
| 1.00 0.83 0.74 0.87 0.88 0.72 0.65 |
| --- |
E
F
G
H
IPEC-J2 cell
150
120
90
Cell viability (%)
60
30
0
0
5
10
20
30
40
100
ns
TNF-α, ng/ml
IPEC-J2 cell
11.5
11.0
10.5
10.0
9.5
9.0
0
100
40
5
10
20
30
Virus RNA copies log10
TNF-α, ng/ml
12.0
***
IPEC-J2 cell
6
5
4
0
5
10
20
30
40
100
TNF-α, ng/ml
7
***
Virus titers log10 (PFUs/ml)
IPEC-J2 cell
ng/ml
TNF-α
30
0
5
10
20
40
100
+
+
+
+
+
+
+
PDCoV
kDa
40
PDCoV-N
42
β-actin
N/β-actin
| 1.00 1.15 1.22 1.19 1.21 0.98 0.57 |
| --- |
