# Supplementary material for: Deltacoronavirus Modulates circRNA cGLIS3 Metabolism to Evade Host Antiviral Response
Source: Adv Sci (Weinh). 2026 Jul 27:e76822. Online ahead of print. doi: 10.1002/advs.76822 (PMC13403727; doi:10.1002/advs.76822)
Supplement: Supplementary file 3 — Supporting File 3: advs76822‐sup‐0001‐TableS1‐S3.pptx. [file ADVS-9999-e76822-s003.pptx]

## Slide 1
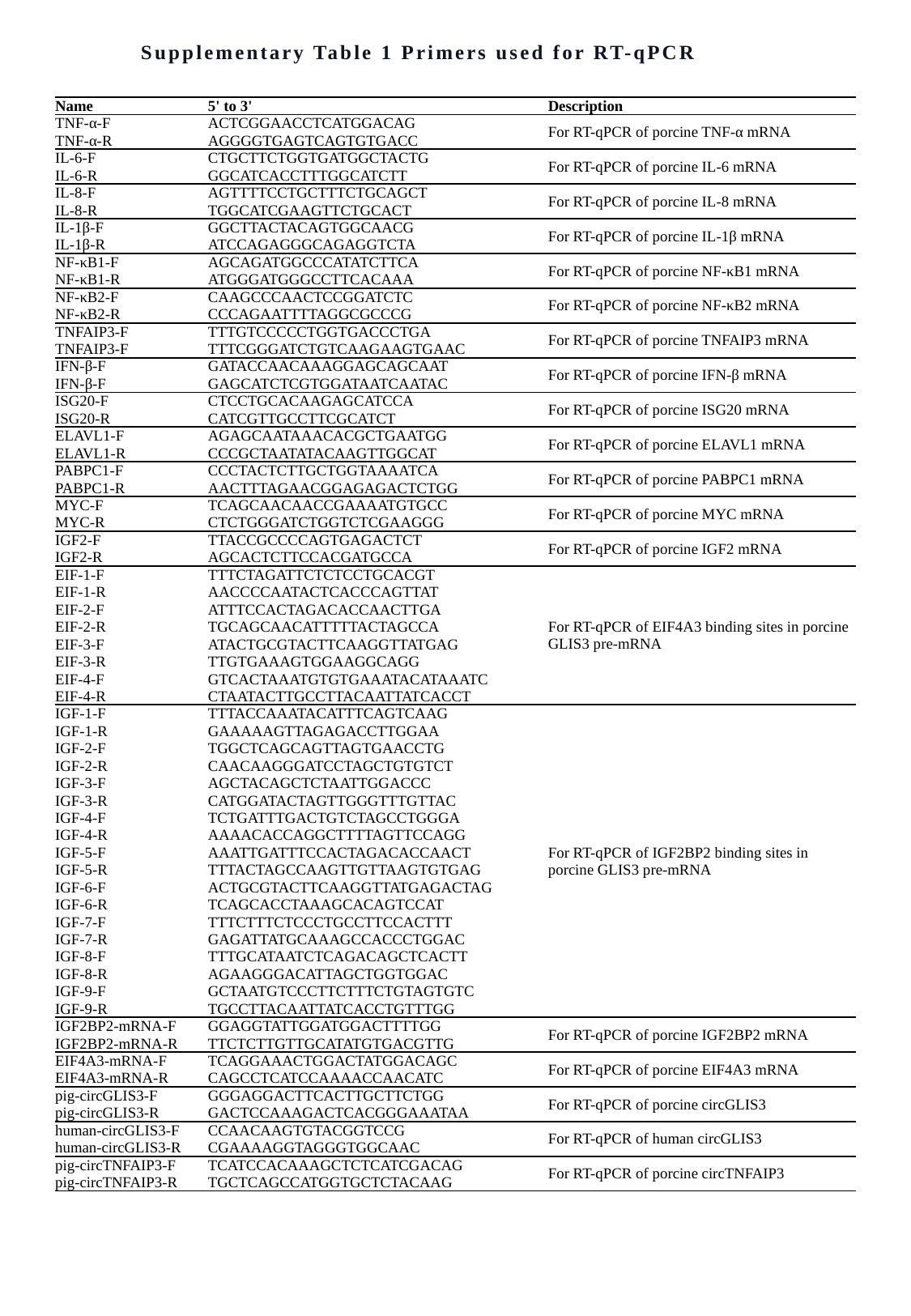

Supplementary Table 1 Primers used for RT-qPCR
| Name | 5' to 3' | Description |
| --- | --- | --- |
| TNF-α-F | ACTCGGAACCTCATGGACAG | For RT-qPCR of porcine TNF-α mRNA |
| TNF-α-R | AGGGGTGAGTCAGTGTGACC | |
| IL-6-F | CTGCTTCTGGTGATGGCTACTG | For RT-qPCR of porcine IL-6 mRNA |
| IL-6-R | GGCATCACCTTTGGCATCTT | |
| IL-8-F | AGTTTTCCTGCTTTCTGCAGCT | For RT-qPCR of porcine IL-8 mRNA |
| IL-8-R | TGGCATCGAAGTTCTGCACT | |
| IL-1β-F | GGCTTACTACAGTGGCAACG | For RT-qPCR of porcine IL-1β mRNA |
| IL-1β-R | ATCCAGAGGGCAGAGGTCTA | |
| NF-κB1-F | AGCAGATGGCCCATATCTTCA | For RT-qPCR of porcine NF-κB1 mRNA |
| NF-κB1-R | ATGGGATGGGCCTTCACAAA | |
| NF-κB2-F | CAAGCCCAACTCCGGATCTC | For RT-qPCR of porcine NF-κB2 mRNA |
| NF-κB2-R | CCCAGAATTTTAGGCGCCCG | |
| TNFAIP3-F | TTTGTCCCCCTGGTGACCCTGA | For RT-qPCR of porcine TNFAIP3 mRNA |
| TNFAIP3-F | TTTCGGGATCTGTCAAGAAGTGAAC | |
| IFN-β-F | GATACCAACAAAGGAGCAGCAAT | For RT-qPCR of porcine IFN-β mRNA |
| IFN-β-F | GAGCATCTCGTGGATAATCAATAC | |
| ISG20-F | CTCCTGCACAAGAGCATCCA | For RT-qPCR of porcine ISG20 mRNA |
| ISG20-R | CATCGTTGCCTTCGCATCT | |
| ELAVL1-F | AGAGCAATAAACACGCTGAATGG | For RT-qPCR of porcine ELAVL1 mRNA |
| ELAVL1-R | CCCGCTAATATACAAGTTGGCAT | |
| PABPC1-F | CCCTACTCTTGCTGGTAAAATCA | For RT-qPCR of porcine PABPC1 mRNA |
| PABPC1-R | AACTTTAGAACGGAGAGACTCTGG | |
| MYC-F | TCAGCAACAACCGAAAATGTGCC | For RT-qPCR of porcine MYC mRNA |
| MYC-R | CTCTGGGATCTGGTCTCGAAGGG | |
| IGF2-F | TTACCGCCCCAGTGAGACTCT | For RT-qPCR of porcine IGF2 mRNA |
| IGF2-R | AGCACTCTTCCACGATGCCA | |
| EIF-1-F | TTTCTAGATTCTCTCCTGCACGT | For RT-qPCR of EIF4A3 binding sites in porcine GLIS3 pre-mRNA |
| EIF-1-R | AACCCCAATACTCACCCAGTTAT | |
| EIF-2-F | ATTTCCACTAGACACCAACTTGA | |
| EIF-2-R | TGCAGCAACATTTTTACTAGCCA | |
| EIF-3-F | ATACTGCGTACTTCAAGGTTATGAG | |
| EIF-3-R | TTGTGAAAGTGGAAGGCAGG | |
| EIF-4-F | GTCACTAAATGTGTGAAATACATAAATC | |
| EIF-4-R | CTAATACTTGCCTTACAATTATCACCT | |
| IGF-1-F | TTTACCAAATACATTTCAGTCAAG | For RT-qPCR of IGF2BP2 binding sites in porcine GLIS3 pre-mRNA |
| IGF-1-R | GAAAAAGTTAGAGACCTTGGAA | |
| IGF-2-F | TGGCTCAGCAGTTAGTGAACCTG | |
| IGF-2-R | CAACAAGGGATCCTAGCTGTGTCT | |
| IGF-3-F | AGCTACAGCTCTAATTGGACCC | |
| IGF-3-R | CATGGATACTAGTTGGGTTTGTTAC | |
| IGF-4-F | TCTGATTTGACTGTCTAGCCTGGGA | |
| IGF-4-R | AAAACACCAGGCTTTTAGTTCCAGG | |
| IGF-5-F | AAATTGATTTCCACTAGACACCAACT | |
| IGF-5-R | TTTACTAGCCAAGTTGTTAAGTGTGAG | |
| IGF-6-F | ACTGCGTACTTCAAGGTTATGAGACTAG | |
| IGF-6-R | TCAGCACCTAAAGCACAGTCCAT | |
| IGF-7-F | TTTCTTTCTCCCTGCCTTCCACTTT | |
| IGF-7-R | GAGATTATGCAAAGCCACCCTGGAC | |
| IGF-8-F | TTTGCATAATCTCAGACAGCTCACTT | |
| IGF-8-R | AGAAGGGACATTAGCTGGTGGAC | |
| IGF-9-F | GCTAATGTCCCTTCTTTCTGTAGTGTC | |
| IGF-9-R | TGCCTTACAATTATCACCTGTTTGG | |
| IGF2BP2-mRNA-F | GGAGGTATTGGATGGACTTTTGG | For RT-qPCR of porcine IGF2BP2 mRNA |
| IGF2BP2-mRNA-R | TTCTCTTGTTGCATATGTGACGTTG | |
| EIF4A3-mRNA-F | TCAGGAAACTGGACTATGGACAGC | For RT-qPCR of porcine EIF4A3 mRNA |
| EIF4A3-mRNA-R | CAGCCTCATCCAAAACCAACATC | |
| pig-circGLIS3-F | GGGAGGACTTCACTTGCTTCTGG | For RT-qPCR of porcine circGLIS3 |
| pig-circGLIS3-R | GACTCCAAAGACTCACGGGAAATAA | |
| human-circGLIS3-F | CCAACAAGTGTACGGTCCG | For RT-qPCR of human circGLIS3 |
| human-circGLIS3-R | CGAAAAGGTAGGGTGGCAAC | |
| pig-circTNFAIP3-F | TCATCCACAAAGCTCTCATCGACAG | For RT-qPCR of porcine circTNFAIP3 |
| pig-circTNFAIP3-R | TGCTCAGCCATGGTGCTCTACAAG | |

## Slide 2
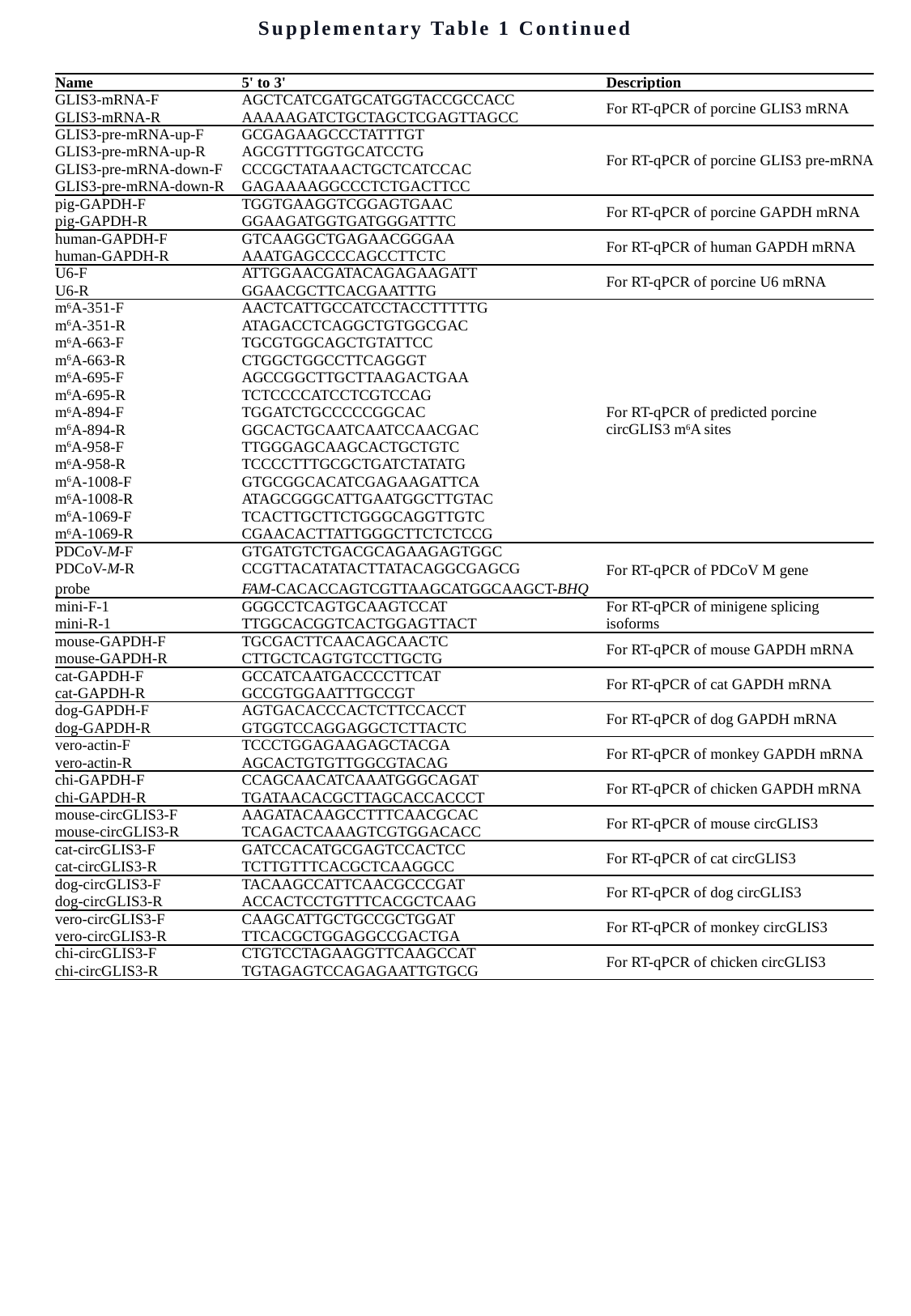

Supplementary Table 1 Continued
| Name | 5' to 3' | Description |
| --- | --- | --- |
| GLIS3-mRNA-F | AGCTCATCGATGCATGGTACCGCCACC | For RT-qPCR of porcine GLIS3 mRNA |
| GLIS3-mRNA-R | AAAAAGATCTGCTAGCTCGAGTTAGCC | |
| GLIS3-pre-mRNA-up-F | GCGAGAAGCCCTATTTGT | For RT-qPCR of porcine GLIS3 pre-mRNA |
| GLIS3-pre-mRNA-up-R | AGCGTTTGGTGCATCCTG | |
| GLIS3-pre-mRNA-down-F | CCCGCTATAAACTGCTCATCCAC | |
| GLIS3-pre-mRNA-down-R | GAGAAAAGGCCCTCTGACTTCC | |
| pig-GAPDH-F | TGGTGAAGGTCGGAGTGAAC | For RT-qPCR of porcine GAPDH mRNA |
| pig-GAPDH-R | GGAAGATGGTGATGGGATTTC | |
| human-GAPDH-F | GTCAAGGCTGAGAACGGGAA | For RT-qPCR of human GAPDH mRNA |
| human-GAPDH-R | AAATGAGCCCCAGCCTTCTC | |
| U6-F | ATTGGAACGATACAGAGAAGATT | For RT-qPCR of porcine U6 mRNA |
| U6-R | GGAACGCTTCACGAATTTG | |
| m6A-351-F | AACTCATTGCCATCCTACCTTTTTG | For RT-qPCR of predicted porcine circGLIS3 m6A sites |
| m6A-351-R | ATAGACCTCAGGCTGTGGCGAC | |
| m6A-663-F | TGCGTGGCAGCTGTATTCC | |
| m6A-663-R | CTGGCTGGCCTTCAGGGT | |
| m6A-695-F | AGCCGGCTTGCTTAAGACTGAA | |
| m6A-695-R | TCTCCCCATCCTCGTCCAG | |
| m6A-894-F | TGGATCTGCCCCCGGCAC | |
| m6A-894-R | GGCACTGCAATCAATCCAACGAC | |
| m6A-958-F | TTGGGAGCAAGCACTGCTGTC | |
| m6A-958-R | TCCCCTTTGCGCTGATCTATATG | |
| m6A-1008-F | GTGCGGCACATCGAGAAGATTCA | |
| m6A-1008-R | ATAGCGGGCATTGAATGGCTTGTAC | |
| m6A-1069-F | TCACTTGCTTCTGGGCAGGTTGTC | |
| m6A-1069-R | CGAACACTTATTGGGCTTCTCTCCG | |
| PDCoV-M-F | GTGATGTCTGACGCAGAAGAGTGGC | For RT-qPCR of PDCoV M gene |
| PDCoV-M-R | CCGTTACATATACTTATACAGGCGAGCG | |
| probe | FAM-CACACCAGTCGTTAAGCATGGCAAGCT-BHQ | |
| mini-F-1 | GGGCCTCAGTGCAAGTCCAT | For RT-qPCR of minigene splicing isoforms |
| mini-R-1 | TTGGCACGGTCACTGGAGTTACT | |
| mouse-GAPDH-F | TGCGACTTCAACAGCAACTC | For RT-qPCR of mouse GAPDH mRNA |
| mouse-GAPDH-R | CTTGCTCAGTGTCCTTGCTG | |
| cat-GAPDH-F | GCCATCAATGACCCCTTCAT | For RT-qPCR of cat GAPDH mRNA |
| cat-GAPDH-R | GCCGTGGAATTTGCCGT | |
| dog-GAPDH-F | AGTGACACCCACTCTTCCACCT | For RT-qPCR of dog GAPDH mRNA |
| dog-GAPDH-R | GTGGTCCAGGAGGCTCTTACTC | |
| vero-actin-F | TCCCTGGAGAAGAGCTACGA | For RT-qPCR of monkey GAPDH mRNA |
| vero-actin-R | AGCACTGTGTTGGCGTACAG | |
| chi-GAPDH-F | CCAGCAACATCAAATGGGCAGAT | For RT-qPCR of chicken GAPDH mRNA |
| chi-GAPDH-R | TGATAACACGCTTAGCACCACCCT | |
| mouse-circGLIS3-F | AAGATACAAGCCTTTCAACGCAC | For RT-qPCR of mouse circGLIS3 |
| mouse-circGLIS3-R | TCAGACTCAAAGTCGTGGACACC | |
| cat-circGLIS3-F | GATCCACATGCGAGTCCACTCC | For RT-qPCR of cat circGLIS3 |
| cat-circGLIS3-R | TCTTGTTTCACGCTCAAGGCC | |
| dog-circGLIS3-F | TACAAGCCATTCAACGCCCGAT | For RT-qPCR of dog circGLIS3 |
| dog-circGLIS3-R | ACCACTCCTGTTTCACGCTCAAG | |
| vero-circGLIS3-F | CAAGCATTGCTGCCGCTGGAT | For RT-qPCR of monkey circGLIS3 |
| vero-circGLIS3-R | TTCACGCTGGAGGCCGACTGA | |
| chi-circGLIS3-F | CTGTCCTAGAAGGTTCAAGCCAT | For RT-qPCR of chicken circGLIS3 |
| chi-circGLIS3-R | TGTAGAGTCCAGAGAATTGTGCG | |

## Slide 3
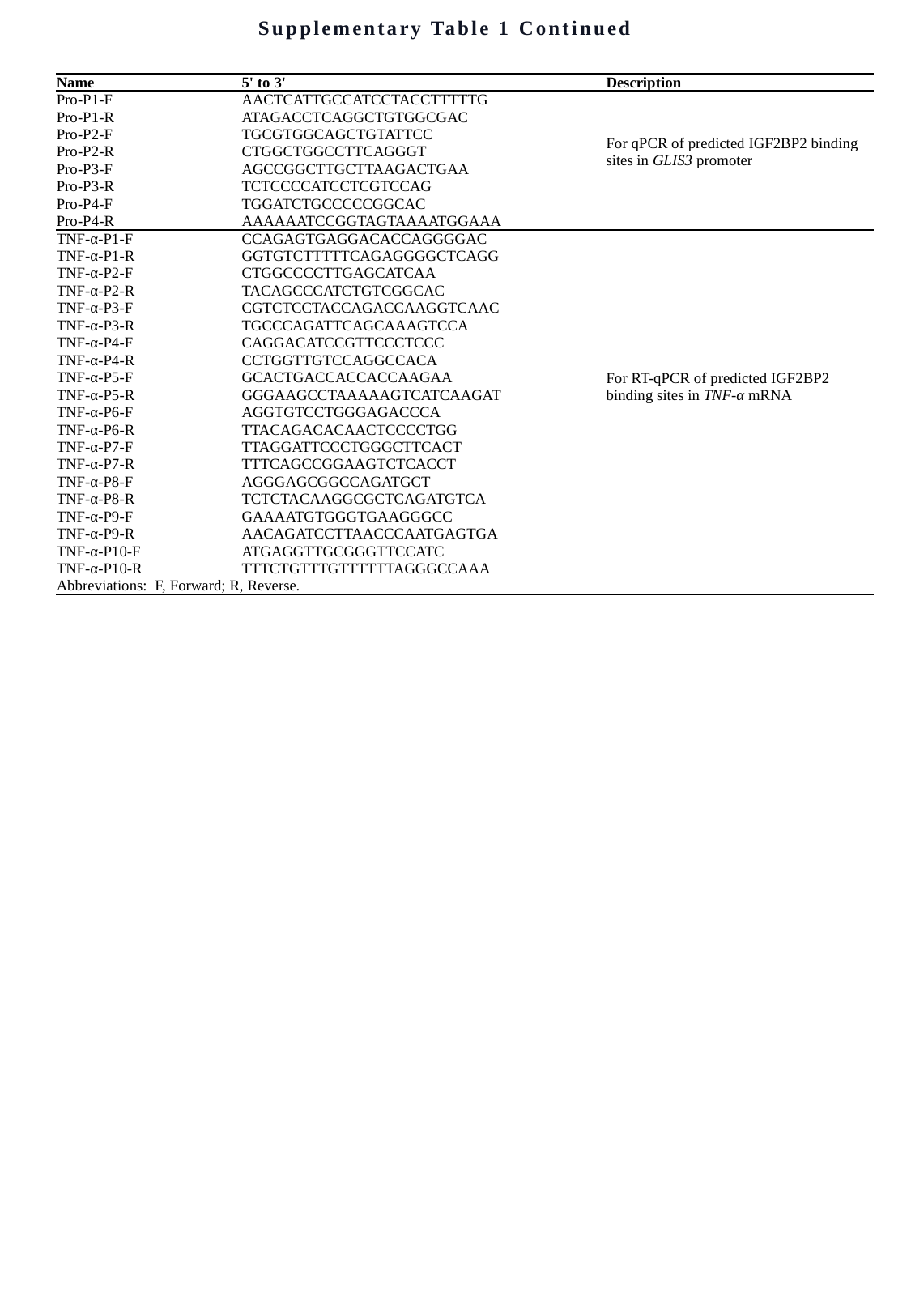

Supplementary Table 1 Continued
| Name | 5' to 3' | Description |
| --- | --- | --- |
| Pro-P1-F | AACTCATTGCCATCCTACCTTTTTG | For qPCR of predicted IGF2BP2 binding sites in GLIS3 promoter |
| Pro-P1-R | ATAGACCTCAGGCTGTGGCGAC | |
| Pro-P2-F | TGCGTGGCAGCTGTATTCC | |
| Pro-P2-R | CTGGCTGGCCTTCAGGGT | |
| Pro-P3-F | AGCCGGCTTGCTTAAGACTGAA | |
| Pro-P3-R | TCTCCCCATCCTCGTCCAG | |
| Pro-P4-F | TGGATCTGCCCCCGGCAC | |
| Pro-P4-R | AAAAAATCCGGTAGTAAAATGGAAA | |
| TNF-α-P1-F | CCAGAGTGAGGACACCAGGGGAC | |
| TNF-α-P1-R | GGTGTCTTTTTCAGAGGGGCTCAGG | |
| TNF-α-P2-F | CTGGCCCCTTGAGCATCAA | |
| TNF-α-P2-R | TACAGCCCATCTGTCGGCAC | |
| TNF-α-P3-F | CGTCTCCTACCAGACCAAGGTCAAC | |
| TNF-α-P3-R | TGCCCAGATTCAGCAAAGTCCA | |
| TNF-α-P4-F | CAGGACATCCGTTCCCTCCC | |
| TNF-α-P4-R | CCTGGTTGTCCAGGCCACA | |
| TNF-α-P5-F | GCACTGACCACCACCAAGAA | For RT-qPCR of predicted IGF2BP2 binding sites in TNF-α mRNA |
| TNF-α-P5-R | GGGAAGCCTAAAAAGTCATCAAGAT | |
| TNF-α-P6-F | AGGTGTCCTGGGAGACCCA | |
| TNF-α-P6-R | TTACAGACACAACTCCCCTGG | |
| TNF-α-P7-F | TTAGGATTCCCTGGGCTTCACT | |
| TNF-α-P7-R | TTTCAGCCGGAAGTCTCACCT | |
| TNF-α-P8-F | AGGGAGCGGCCAGATGCT | |
| TNF-α-P8-R | TCTCTACAAGGCGCTCAGATGTCA | |
| TNF-α-P9-F | GAAAATGTGGGTGAAGGGCC | |
| TNF-α-P9-R | AACAGATCCTTAACCCAATGAGTGA | |
| TNF-α-P10-F | ATGAGGTTGCGGGTTCCATC | |
| TNF-α-P10-R | TTTCTGTTTGTTTTTTAGGGCCAAA | |
| Abbreviations: F, Forward; R, Reverse. | | |

## Slide 4
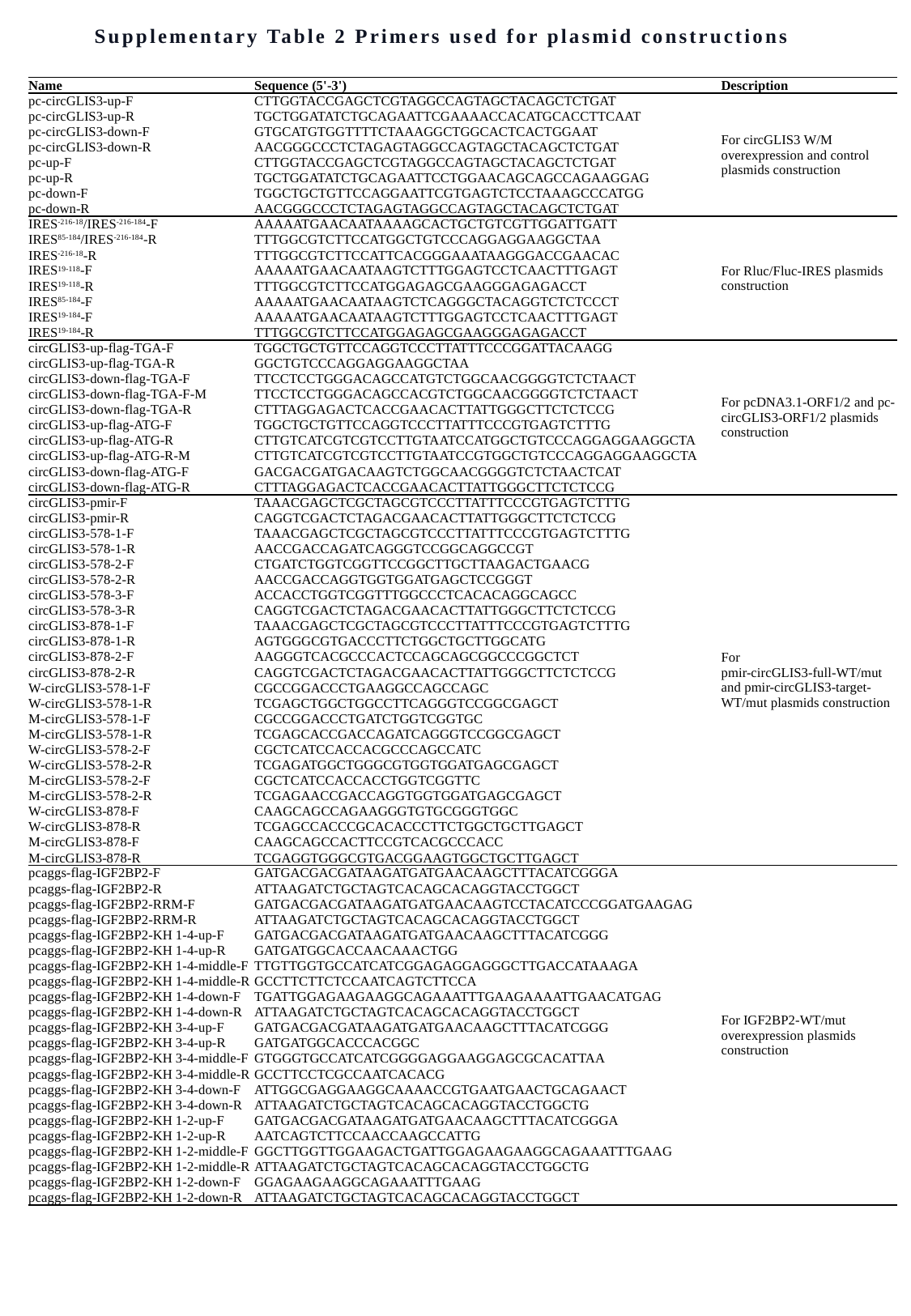

Supplementary Table 2 Primers used for plasmid constructions
| Name | Sequence (5'-3') | Description |
| --- | --- | --- |
| pc-circGLIS3-up-F | CTTGGTACCGAGCTCGTAGGCCAGTAGCTACAGCTCTGAT | For circGLIS3 W/M overexpression and control plasmids construction |
| pc-circGLIS3-up-R | TGCTGGATATCTGCAGAATTCGAAAACCACATGCACCTTCAAT | |
| pc-circGLIS3-down-F | GTGCATGTGGTTTTCTAAAGGCTGGCACTCACTGGAAT | |
| pc-circGLIS3-down-R | AACGGGCCCTCTAGAGTAGGCCAGTAGCTACAGCTCTGAT | |
| pc-up-F | CTTGGTACCGAGCTCGTAGGCCAGTAGCTACAGCTCTGAT | |
| pc-up-R | TGCTGGATATCTGCAGAATTCCTGGAACAGCAGCCAGAAGGAG | |
| pc-down-F | TGGCTGCTGTTCCAGGAATTCGTGAGTCTCCTAAAGCCCATGG | |
| pc-down-R | AACGGGCCCTCTAGAGTAGGCCAGTAGCTACAGCTCTGAT | |
| IRES-216-18/IRES-216-184-F | AAAAATGAACAATAAAAGCACTGCTGTCGTTGGATTGATT | For Rluc/Fluc-IRES plasmids construction |
| IRES85-184/IRES-216-184-R | TTTGGCGTCTTCCATGGCTGTCCCAGGAGGAAGGCTAA | |
| IRES-216-18-R | TTTGGCGTCTTCCATTCACGGGAAATAAGGGACCGAACAC | |
| IRES19-118-F | AAAAATGAACAATAAGTCTTTGGAGTCCTCAACTTTGAGT | |
| IRES19-118-R | TTTGGCGTCTTCCATGGAGAGCGAAGGGAGAGACCT | |
| IRES85-184-F | AAAAATGAACAATAAGTCTCAGGGCTACAGGTCTCTCCCT | |
| IRES19-184-F | AAAAATGAACAATAAGTCTTTGGAGTCCTCAACTTTGAGT | |
| IRES19-184-R | TTTGGCGTCTTCCATGGAGAGCGAAGGGAGAGACCT | |
| circGLIS3-up-flag-TGA-F | TGGCTGCTGTTCCAGGTCCCTTATTTCCCGGATTACAAGG | For pcDNA3.1-ORF1/2 and pc-circGLIS3-ORF1/2 plasmids construction |
| circGLIS3-up-flag-TGA-R | GGCTGTCCCAGGAGGAAGGCTAA | |
| circGLIS3-down-flag-TGA-F | TTCCTCCTGGGACAGCCATGTCTGGCAACGGGGTCTCTAACT | |
| circGLIS3-down-flag-TGA-F-M | TTCCTCCTGGGACAGCCACGTCTGGCAACGGGGTCTCTAACT | |
| circGLIS3-down-flag-TGA-R | CTTTAGGAGACTCACCGAACACTTATTGGGCTTCTCTCCG | |
| circGLIS3-up-flag-ATG-F | TGGCTGCTGTTCCAGGTCCCTTATTTCCCGTGAGTCTTTG | |
| circGLIS3-up-flag-ATG-R | CTTGTCATCGTCGTCCTTGTAATCCATGGCTGTCCCAGGAGGAAGGCTA | |
| circGLIS3-up-flag-ATG-R-M | CTTGTCATCGTCGTCCTTGTAATCCGTGGCTGTCCCAGGAGGAAGGCTA | |
| circGLIS3-down-flag-ATG-F | GACGACGATGACAAGTCTGGCAACGGGGTCTCTAACTCAT | |
| circGLIS3-down-flag-ATG-R | CTTTAGGAGACTCACCGAACACTTATTGGGCTTCTCTCCG | |
| circGLIS3-pmir-F | TAAACGAGCTCGCTAGCGTCCCTTATTTCCCGTGAGTCTTTG | For pmir-circGLIS3-full-WT/mut and pmir-circGLIS3-target-WT/mut plasmids construction |
| circGLIS3-pmir-R | CAGGTCGACTCTAGACGAACACTTATTGGGCTTCTCTCCG | |
| circGLIS3-578-1-F | TAAACGAGCTCGCTAGCGTCCCTTATTTCCCGTGAGTCTTTG | |
| circGLIS3-578-1-R | AACCGACCAGATCAGGGTCCGGCAGGCCGT | |
| circGLIS3-578-2-F | CTGATCTGGTCGGTTCCGGCTTGCTTAAGACTGAACG | |
| circGLIS3-578-2-R | AACCGACCAGGTGGTGGATGAGCTCCGGGT | |
| circGLIS3-578-3-F | ACCACCTGGTCGGTTTGGCCCTCACACAGGCAGCC | |
| circGLIS3-578-3-R | CAGGTCGACTCTAGACGAACACTTATTGGGCTTCTCTCCG | |
| circGLIS3-878-1-F | TAAACGAGCTCGCTAGCGTCCCTTATTTCCCGTGAGTCTTTG | |
| circGLIS3-878-1-R | AGTGGGCGTGACCCTTCTGGCTGCTTGGCATG | |
| circGLIS3-878-2-F | AAGGGTCACGCCCACTCCAGCAGCGGCCCGGCTCT | |
| circGLIS3-878-2-R | CAGGTCGACTCTAGACGAACACTTATTGGGCTTCTCTCCG | |
| W-circGLIS3-578-1-F | CGCCGGACCCTGAAGGCCAGCCAGC | |
| W-circGLIS3-578-1-R | TCGAGCTGGCTGGCCTTCAGGGTCCGGCGAGCT | |
| M-circGLIS3-578-1-F | CGCCGGACCCTGATCTGGTCGGTGC | |
| M-circGLIS3-578-1-R | TCGAGCACCGACCAGATCAGGGTCCGGCGAGCT | |
| W-circGLIS3-578-2-F | CGCTCATCCACCACGCCCAGCCATC | |
| W-circGLIS3-578-2-R | TCGAGATGGCTGGGCGTGGTGGATGAGCGAGCT | |
| M-circGLIS3-578-2-F | CGCTCATCCACCACCTGGTCGGTTC | |
| M-circGLIS3-578-2-R | TCGAGAACCGACCAGGTGGTGGATGAGCGAGCT | |
| W-circGLIS3-878-F | CAAGCAGCCAGAAGGGTGTGCGGGTGGC | |
| W-circGLIS3-878-R | TCGAGCCACCCGCACACCCTTCTGGCTGCTTGAGCT | |
| M-circGLIS3-878-F | CAAGCAGCCACTTCCGTCACGCCCACC | |
| M-circGLIS3-878-R | TCGAGGTGGGCGTGACGGAAGTGGCTGCTTGAGCT | |
| pcaggs-flag-IGF2BP2-F | GATGACGACGATAAGATGATGAACAAGCTTTACATCGGGA | For IGF2BP2-WT/mut overexpression plasmids construction |
| pcaggs-flag-IGF2BP2-R | ATTAAGATCTGCTAGTCACAGCACAGGTACCTGGCT | |
| pcaggs-flag-IGF2BP2-RRM-F | GATGACGACGATAAGATGATGAACAAGTCCTACATCCCGGATGAAGAG | |
| pcaggs-flag-IGF2BP2-RRM-R | ATTAAGATCTGCTAGTCACAGCACAGGTACCTGGCT | |
| pcaggs-flag-IGF2BP2-KH 1-4-up-F | GATGACGACGATAAGATGATGAACAAGCTTTACATCGGG | |
| pcaggs-flag-IGF2BP2-KH 1-4-up-R | GATGATGGCACCAACAAACTGG | |
| pcaggs-flag-IGF2BP2-KH 1-4-middle-F | TTGTTGGTGCCATCATCGGAGAGGAGGGCTTGACCATAAAGA | |
| pcaggs-flag-IGF2BP2-KH 1-4-middle-R | GCCTTCTTCTCCAATCAGTCTTCCA | |
| pcaggs-flag-IGF2BP2-KH 1-4-down-F | TGATTGGAGAAGAAGGCAGAAATTTGAAGAAAATTGAACATGAG | |
| pcaggs-flag-IGF2BP2-KH 1-4-down-R | ATTAAGATCTGCTAGTCACAGCACAGGTACCTGGCT | |
| pcaggs-flag-IGF2BP2-KH 3-4-up-F | GATGACGACGATAAGATGATGAACAAGCTTTACATCGGG | |
| pcaggs-flag-IGF2BP2-KH 3-4-up-R | GATGATGGCACCCACGGC | |
| pcaggs-flag-IGF2BP2-KH 3-4-middle-F | GTGGGTGCCATCATCGGGGAGGAAGGAGCGCACATTAA | |
| pcaggs-flag-IGF2BP2-KH 3-4-middle-R | GCCTTCCTCGCCAATCACACG | |
| pcaggs-flag-IGF2BP2-KH 3-4-down-F | ATTGGCGAGGAAGGCAAAACCGTGAATGAACTGCAGAACT | |
| pcaggs-flag-IGF2BP2-KH 3-4-down-R | ATTAAGATCTGCTAGTCACAGCACAGGTACCTGGCTG | |
| pcaggs-flag-IGF2BP2-KH 1-2-up-F | GATGACGACGATAAGATGATGAACAAGCTTTACATCGGGA | |
| pcaggs-flag-IGF2BP2-KH 1-2-up-R | AATCAGTCTTCCAACCAAGCCATTG | |
| pcaggs-flag-IGF2BP2-KH 1-2-middle-F | GGCTTGGTTGGAAGACTGATTGGAGAAGAAGGCAGAAATTTGAAG | |
| pcaggs-flag-IGF2BP2-KH 1-2-middle-R | ATTAAGATCTGCTAGTCACAGCACAGGTACCTGGCTG | |
| pcaggs-flag-IGF2BP2-KH 1-2-down-F | GGAGAAGAAGGCAGAAATTTGAAG | |
| pcaggs-flag-IGF2BP2-KH 1-2-down-R | ATTAAGATCTGCTAGTCACAGCACAGGTACCTGGCT | |

## Slide 5
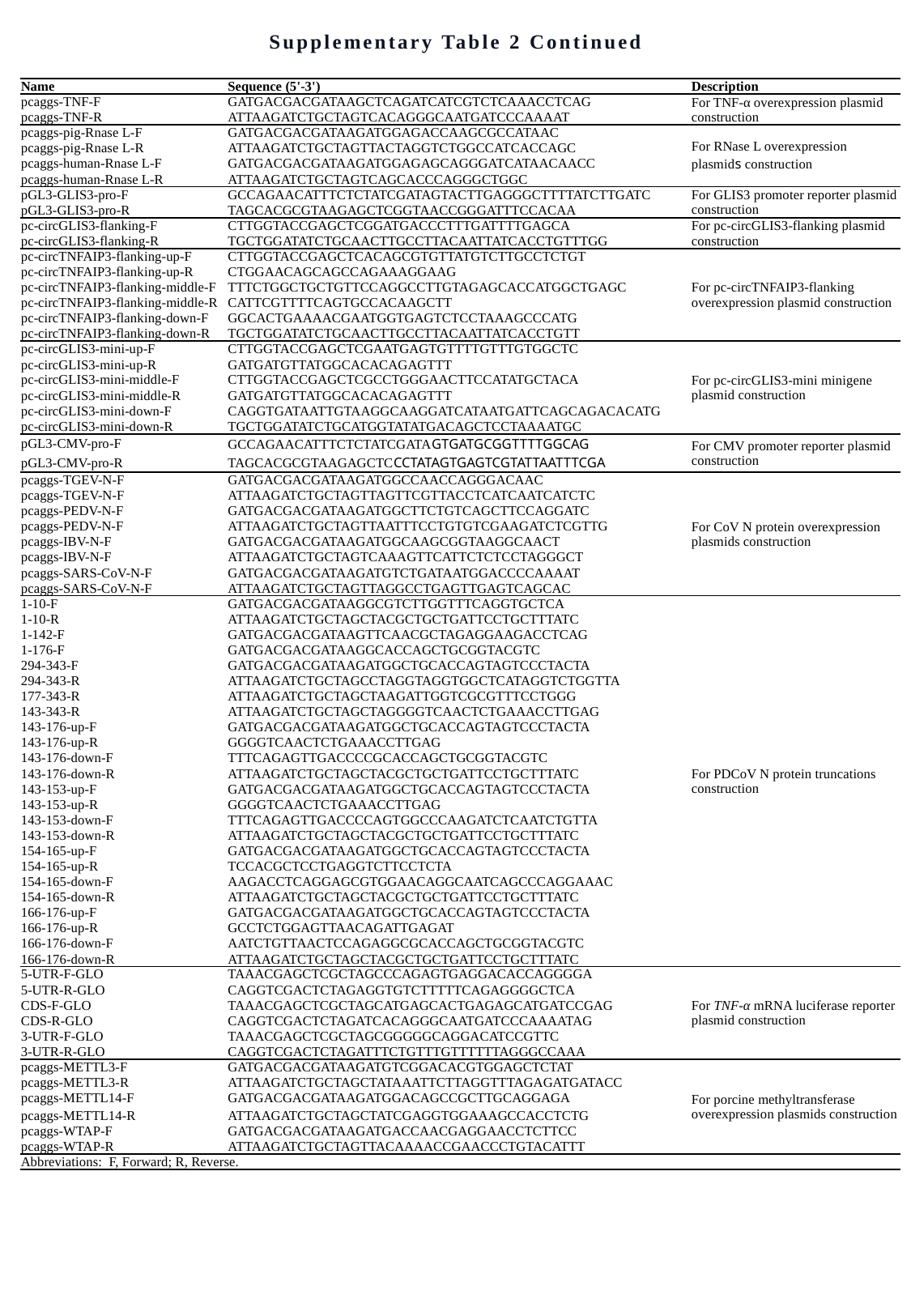

Supplementary Table 2 Continued
| Name | Sequence (5'-3') | Description |
| --- | --- | --- |
| pcaggs-TNF-F | GATGACGACGATAAGCTCAGATCATCGTCTCAAACCTCAG | For TNF-α overexpression plasmid construction |
| pcaggs-TNF-R | ATTAAGATCTGCTAGTCACAGGGCAATGATCCCAAAAT | |
| pcaggs-pig-Rnase L-F | GATGACGACGATAAGATGGAGACCAAGCGCCATAAC | For RNase L overexpression plasmids construction |
| pcaggs-pig-Rnase L-R | ATTAAGATCTGCTAGTTACTAGGTCTGGCCATCACCAGC | |
| pcaggs-human-Rnase L-F | GATGACGACGATAAGATGGAGAGCAGGGATCATAACAACC | |
| pcaggs-human-Rnase L-R | ATTAAGATCTGCTAGTCAGCACCCAGGGCTGGC | |
| pGL3-GLIS3-pro-F | GCCAGAACATTTCTCTATCGATAGTACTTGAGGGCTTTTATCTTGATC | For GLIS3 promoter reporter plasmid construction |
| pGL3-GLIS3-pro-R | TAGCACGCGTAAGAGCTCGGTAACCGGGATTTCCACAA | |
| pc-circGLIS3-flanking-F | CTTGGTACCGAGCTCGGATGACCCTTTGATTTTGAGCA | For pc-circGLIS3-flanking plasmid construction |
| pc-circGLIS3-flanking-R | TGCTGGATATCTGCAACTTGCCTTACAATTATCACCTGTTTGG | |
| pc-circTNFAIP3-flanking-up-F | CTTGGTACCGAGCTCACAGCGTGTTATGTCTTGCCTCTGT | For pc-circTNFAIP3-flanking overexpression plasmid construction |
| pc-circTNFAIP3-flanking-up-R | CTGGAACAGCAGCCAGAAAGGAAG | |
| pc-circTNFAIP3-flanking-middle-F | TTTCTGGCTGCTGTTCCAGGCCTTGTAGAGCACCATGGCTGAGC | |
| pc-circTNFAIP3-flanking-middle-R | CATTCGTTTTCAGTGCCACAAGCTT | |
| pc-circTNFAIP3-flanking-down-F | GGCACTGAAAACGAATGGTGAGTCTCCTAAAGCCCATG | |
| pc-circTNFAIP3-flanking-down-R | TGCTGGATATCTGCAACTTGCCTTACAATTATCACCTGTT | |
| pc-circGLIS3-mini-up-F | CTTGGTACCGAGCTCGAATGAGTGTTTTGTTTGTGGCTC | For pc-circGLIS3-mini minigene plasmid construction |
| pc-circGLIS3-mini-up-R | GATGATGTTATGGCACACAGAGTTT | |
| pc-circGLIS3-mini-middle-F | CTTGGTACCGAGCTCGCCTGGGAACTTCCATATGCTACA | |
| pc-circGLIS3-mini-middle-R | GATGATGTTATGGCACACAGAGTTT | |
| pc-circGLIS3-mini-down-F | CAGGTGATAATTGTAAGGCAAGGATCATAATGATTCAGCAGACACATG | |
| pc-circGLIS3-mini-down-R | TGCTGGATATCTGCATGGTATATGACAGCTCCTAAAATGC | |
| pGL3-CMV-pro-F | GCCAGAACATTTCTCTATCGATAGTGATGCGGTTTTGGCAG | For CMV promoter reporter plasmid construction |
| pGL3-CMV-pro-R | TAGCACGCGTAAGAGCTCCCTATAGTGAGTCGTATTAATTTCGA | |
| pcaggs-TGEV-N-F | GATGACGACGATAAGATGGCCAACCAGGGACAAC | For CoV N protein overexpression plasmids construction |
| pcaggs-TGEV-N-F | ATTAAGATCTGCTAGTTAGTTCGTTACCTCATCAATCATCTC | |
| pcaggs-PEDV-N-F | GATGACGACGATAAGATGGCTTCTGTCAGCTTCCAGGATC | |
| pcaggs-PEDV-N-F | ATTAAGATCTGCTAGTTAATTTCCTGTGTCGAAGATCTCGTTG | |
| pcaggs-IBV-N-F | GATGACGACGATAAGATGGCAAGCGGTAAGGCAACT | |
| pcaggs-IBV-N-F | ATTAAGATCTGCTAGTCAAAGTTCATTCTCTCCTAGGGCT | |
| pcaggs-SARS-CoV-N-F | GATGACGACGATAAGATGTCTGATAATGGACCCCAAAAT | |
| pcaggs-SARS-CoV-N-F | ATTAAGATCTGCTAGTTAGGCCTGAGTTGAGTCAGCAC | |
| 1-10-F | GATGACGACGATAAGGCGTCTTGGTTTCAGGTGCTCA | For PDCoV N protein truncations construction |
| 1-10-R | ATTAAGATCTGCTAGCTACGCTGCTGATTCCTGCTTTATC | |
| 1-142-F | GATGACGACGATAAGTTCAACGCTAGAGGAAGACCTCAG | |
| 1-176-F | GATGACGACGATAAGGCACCAGCTGCGGTACGTC | |
| 294-343-F | GATGACGACGATAAGATGGCTGCACCAGTAGTCCCTACTA | |
| 294-343-R | ATTAAGATCTGCTAGCCTAGGTAGGTGGCTCATAGGTCTGGTTA | |
| 177-343-R | ATTAAGATCTGCTAGCTAAGATTGGTCGCGTTTCCTGGG | |
| 143-343-R | ATTAAGATCTGCTAGCTAGGGGTCAACTCTGAAACCTTGAG | |
| 143-176-up-F | GATGACGACGATAAGATGGCTGCACCAGTAGTCCCTACTA | |
| 143-176-up-R | GGGGTCAACTCTGAAACCTTGAG | |
| 143-176-down-F | TTTCAGAGTTGACCCCGCACCAGCTGCGGTACGTC | |
| 143-176-down-R | ATTAAGATCTGCTAGCTACGCTGCTGATTCCTGCTTTATC | |
| 143-153-up-F | GATGACGACGATAAGATGGCTGCACCAGTAGTCCCTACTA | |
| 143-153-up-R | GGGGTCAACTCTGAAACCTTGAG | |
| 143-153-down-F | TTTCAGAGTTGACCCCAGTGGCCCAAGATCTCAATCTGTTA | |
| 143-153-down-R | ATTAAGATCTGCTAGCTACGCTGCTGATTCCTGCTTTATC | |
| 154-165-up-F | GATGACGACGATAAGATGGCTGCACCAGTAGTCCCTACTA | |
| 154-165-up-R | TCCACGCTCCTGAGGTCTTCCTCTA | |
| 154-165-down-F | AAGACCTCAGGAGCGTGGAACAGGCAATCAGCCCAGGAAAC | |
| 154-165-down-R | ATTAAGATCTGCTAGCTACGCTGCTGATTCCTGCTTTATC | |
| 166-176-up-F | GATGACGACGATAAGATGGCTGCACCAGTAGTCCCTACTA | |
| 166-176-up-R | GCCTCTGGAGTTAACAGATTGAGAT | |
| 166-176-down-F | AATCTGTTAACTCCAGAGGCGCACCAGCTGCGGTACGTC | |
| 166-176-down-R | ATTAAGATCTGCTAGCTACGCTGCTGATTCCTGCTTTATC | |
| 5-UTR-F-GLO | TAAACGAGCTCGCTAGCCCAGAGTGAGGACACCAGGGGA | |
| 5-UTR-R-GLO | CAGGTCGACTCTAGAGGTGTCTTTTTCAGAGGGGCTCA | |
| CDS-F-GLO | TAAACGAGCTCGCTAGCATGAGCACTGAGAGCATGATCCGAG | For TNF-α mRNA luciferase reporter plasmid construction |
| CDS-R-GLO | CAGGTCGACTCTAGATCACAGGGCAATGATCCCAAAATAG | |
| 3-UTR-F-GLO | TAAACGAGCTCGCTAGCGGGGGCAGGACATCCGTTC | |
| 3-UTR-R-GLO | CAGGTCGACTCTAGATTTCTGTTTGTTTTTTAGGGCCAAA | |
| pcaggs-METTL3-F | GATGACGACGATAAGATGTCGGACACGTGGAGCTCTAT | |
| pcaggs-METTL3-R | ATTAAGATCTGCTAGCTATAAATTCTTAGGTTTAGAGATGATACC | |
| pcaggs-METTL14-F | GATGACGACGATAAGATGGACAGCCGCTTGCAGGAGA | For porcine methyltransferase overexpression plasmids construction |
| pcaggs-METTL14-R | ATTAAGATCTGCTAGCTATCGAGGTGGAAAGCCACCTCTG | |
| pcaggs-WTAP-F | GATGACGACGATAAGATGACCAACGAGGAACCTCTTCC | |
| pcaggs-WTAP-R | ATTAAGATCTGCTAGTTACAAAACCGAACCCTGTACATTT | |
| Abbreviations: F, Forward; R, Reverse. | | |

## Slide 6
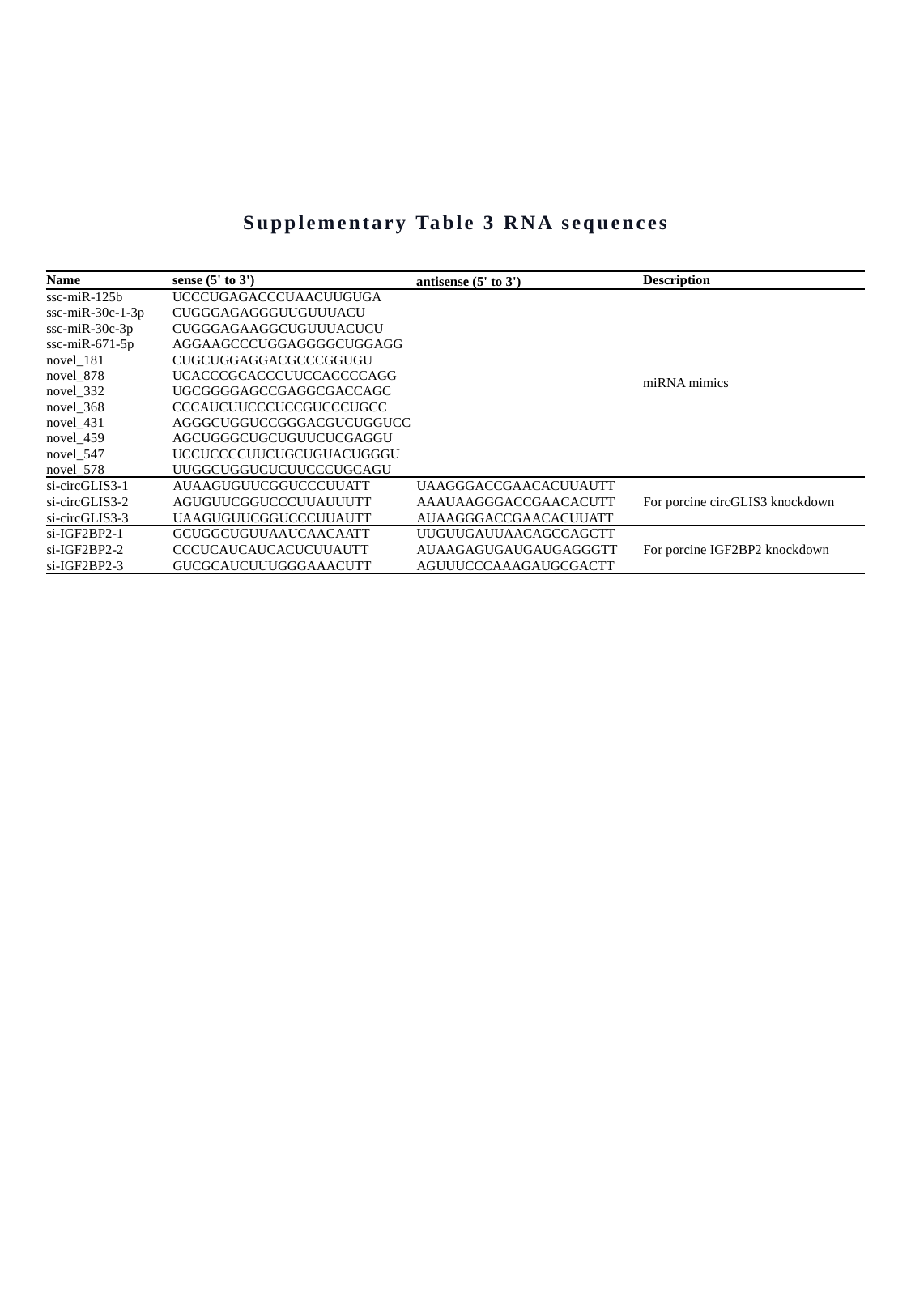

Supplementary Table 3 RNA sequences
| Name | sense (5' to 3') | antisense (5' to 3') | Description |
| --- | --- | --- | --- |
| ssc-miR-125b | UCCCUGAGACCCUAACUUGUGA | | miRNA mimics |
| ssc-miR-30c-1-3p | CUGGGAGAGGGUUGUUUACU | | |
| ssc-miR-30c-3p | CUGGGAGAAGGCUGUUUACUCU | | |
| ssc-miR-671-5p | AGGAAGCCCUGGAGGGGCUGGAGG | | |
| novel\_181 | CUGCUGGAGGACGCCCGGUGU | | |
| novel\_878 | UCACCCGCACCCUUCCACCCCAGG | | |
| novel\_332 | UGCGGGGAGCCGAGGCGACCAGC | | |
| novel\_368 | CCCAUCUUCCCUCCGUCCCUGCC | | |
| novel\_431 | AGGGCUGGUCCGGGACGUCUGGUCC | | |
| novel\_459 | AGCUGGGCUGCUGUUCUCGAGGU | | |
| novel\_547 | UCCUCCCCUUCUGCUGUACUGGGU | | |
| novel\_578 | UUGGCUGGUCUCUUCCCUGCAGU | | |
| si-circGLIS3-1 | AUAAGUGUUCGGUCCCUUATT | UAAGGGACCGAACACUUAUTT | For porcine circGLIS3 knockdown |
| si-circGLIS3-2 | AGUGUUCGGUCCCUUAUUUTT | AAAUAAGGGACCGAACACUTT | |
| si-circGLIS3-3 | UAAGUGUUCGGUCCCUUAUTT | AUAAGGGACCGAACACUUATT | |
| si-IGF2BP2-1 | GCUGGCUGUUAAUCAACAATT | UUGUUGAUUAACAGCCAGCTT | For porcine IGF2BP2 knockdown |
| si-IGF2BP2-2 | CCCUCAUCAUCACUCUUAUTT | AUAAGAGUGAUGAUGAGGGTT | |
| si-IGF2BP2-3 | GUCGCAUCUUUGGGAAACUTT | AGUUUCCCAAAGAUGCGACTT | |
